# Rapid Access to 2,2-Disubstituted Indolines via Dearomative Indolic-Claisen Rearrangement: Concise, Enantioselective Total Synthesis of (+)-Hinckdentine A

**DOI:** 10.1021/jacs.3c03611

**Published:** 2023-06-26

**Authors:** Daler Baidilov, Pavel K. Elkin, Sudhakar Athe, Viresh H. Rawal

**Affiliations:** Department of Chemistry, University of Chicago, 5735 South Ellis Avenue, Chicago, Illinois 60637, United States

## Abstract

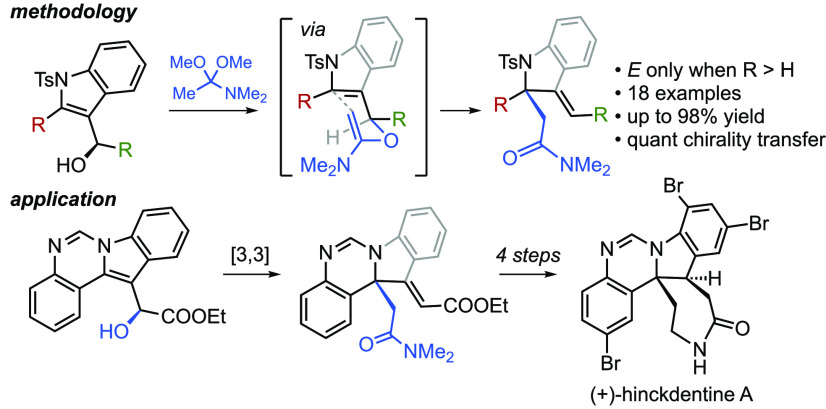

The construction of 2,2-disubstituted indolines has long presented a synthetic challenge without any general solutions. Herein, we report a robust protocol for the dearomative Meerwein–Eschenmoser–Claisen rearrangement of 3-indolyl alcohols that provides efficient access to 2-substituted and 2,2-disubstituted indolines. These versatile subunits are useful for natural product synthesis and medicinal chemistry. The title [3,3] sigmatropic rearrangement proceeds in generally excellent yield and transfers the C3-indolic alcohol chirality to the C2 position with high fidelity, thus providing a reliable method for the construction of enantioenriched 2,2-disubstituted indolines. The power of this methodology is demonstrated through the concise and strategically unique total synthesis of the marine natural product hinckdentine A, which features a dearomative Claisen rearrangement, a diastereocontrolled hydrogenation of the alkene product, a one-pot amide-to-oxime conversion using Vaska’s complex, and a regioselective late-stage tribromination.

## Introduction

**Scheme 1:**
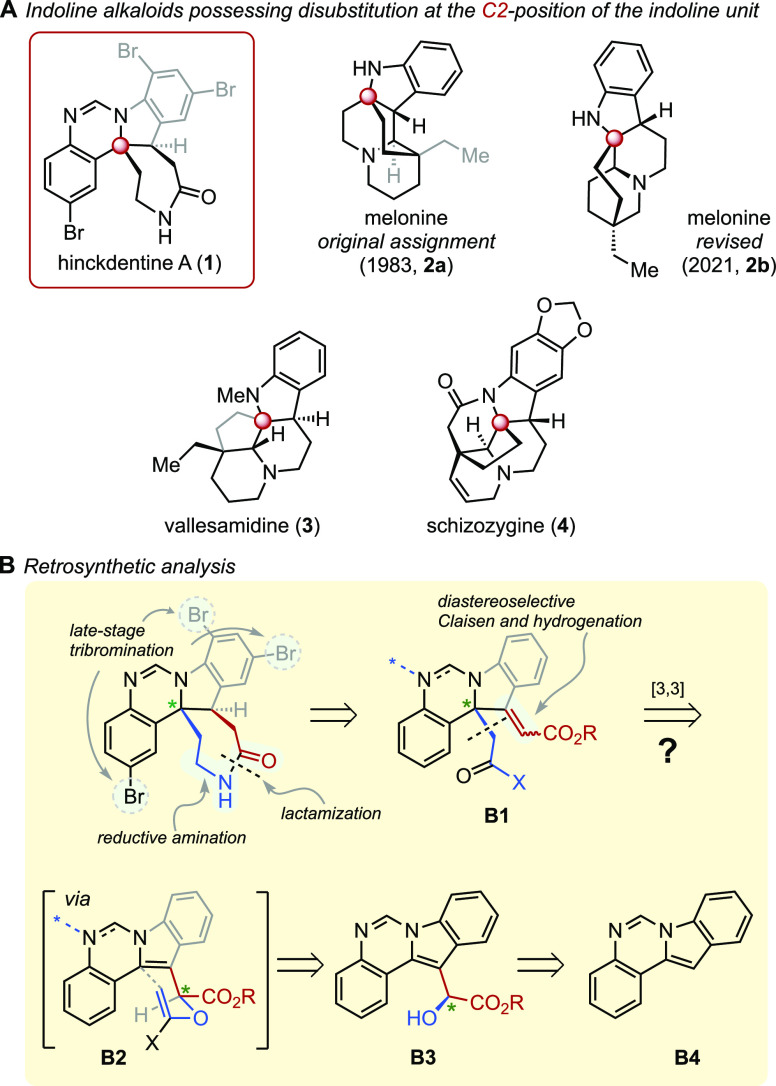
Presence of 2,2-Disubstituted Indolines in Natural Products and a Potential Solution to Hinckdentine A

The potent biological activities of indole alkaloids and the synthetic challenges posed by their intricate architectures have inspired numerous investigators and spurred a plethora of advances in organic synthesis. Our long-standing interest in alkaloids brought to our attention a subset of natural products that possess partial substitution at C3 and disubstitution at C2 of the indoline scaffold—for example, hinckdentine A (**1**),^1^ melonine (**2a** and **2b**),^2^ vallesamidine (**3**), and schizozygine (**4**, Scheme 1A).^3^ Whereas enormous effort has been expended on the synthesis of alkaloids bearing disubstitution at C3 or having fully substituted indoline skeletons,^4^ much less progress has been made toward a generic synthetic protocol to access the indoline motif of natural products such as those shown.^5^ A robust route to 2,2-disubstituted indolines,^6^ we envisioned, would offer rapid entry to these and other natural products. For example, the key structural challenge of hinckdentine A (**1**) could be solved if a dearomative [3,3] sigmatropic rearrangement was developed, as encapsulated through the retrosynthesis shown in Scheme 1B. The target compound would be obtained via selective and efficient elaboration of intermediate **B1**, the product of a Claisen-type rearrangement of alcohol **B3**. The key rearrangement would not only forge the congested C–C bond to the substituted C2 position of the indole unit but it would do so while transferring the benzylic alcohol chirality. We report here the first successful employment of a Meerwein–Eschenmoser–Claisen rearrangement to generate indolines that are mono- or disubstituted at C2. Our results also demonstrate that the stereochemical information embedded in 3-indolyl alcohols is reliably transferred to the newly formed stereogenic center. The power of this transformation is demonstrated through a concise, stereocontrolled enantioselective total synthesis of the alkaloid (+)-hinckdentine A (**1**).

**Scheme 2:**
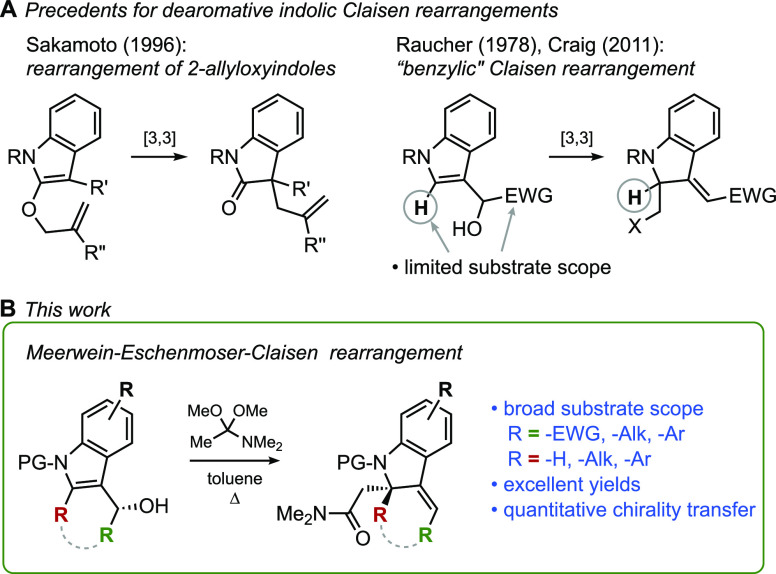
Dearomative Claisen-Type Rearrangements of Indole Substrates

Compared to the classical aliphatic Claisen rearrangement, the related “aromatic Claisen” has received much less attention.^7^ The process is energetically highly demanding due to the disruption of aromatic stabilization upon [3,3] sigmatropic shift, as evidenced by the harsh reaction conditions required to rearrange allyl phenyl ether. Even more challenging is the benzyl version of the aromatic Claisen,^8^ which suffers from a competing [1,3] sigmatropic rearrangement.^9^ Indeed, an earlier study found that benzyl vinyl ether is resistant to undergo rearrangement at temperatures below 260 °C.^10^ Despite its clear usefulness for building complex molecules, the dearomative Claisen rearrangement of indole substrates has seen limited study, with most effort being devoted to the rearrangement of 2-allyloxy indoles, wherein the formation of the carbonyl group provides a strong driving force for the reaction (Scheme 2A).^11,12^ On the other hand, the Claisen rearrangement of 3-indolyl alcohols, which can provide a general route to indolines with a fully substituted C2 position, remains underexplored.^13,14^ The paucity of such dearomative Claisen rearrangements, coupled with the presence of natural products possessing C2-disubstituted indolines as a key structural component, inspired us to investigate the [3,3] sigmatropic rearrangements of 3-indolyl alcohols as a general route to this challenging archi+tectural motif.^15^

## Results and Discussion

### Identification of Claisen Rearrangement of 3-Indolyl Alcohols

**Scheme 3:**
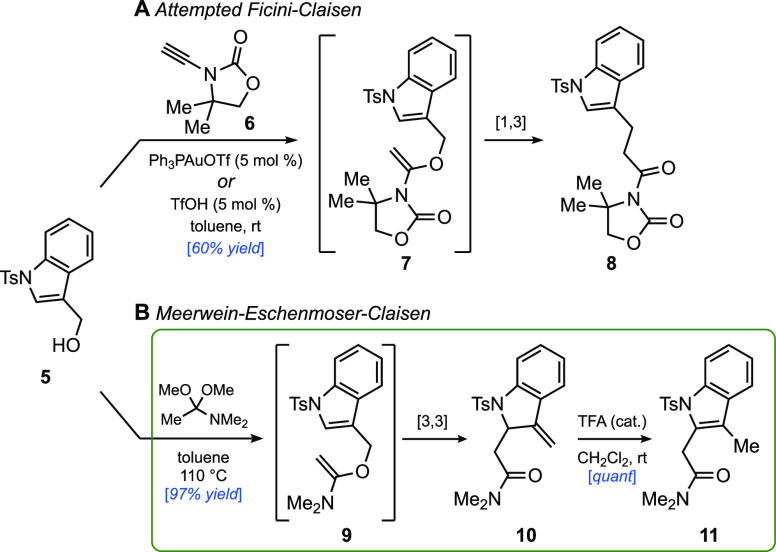
Initial Studies of Claisen Rearrangements of Indolyl Alcohol **5**

In our initial survey, we explored several established protocols to realize the [3,3] Claisen rearrangement of primary alcohol **5**, including the Ireland,^16^ Johnson,^17^ and Bellus (using the gramine derivative of **5**)^18^ variations. Unfortunately, these experiments failed to give the desired [3,3] product; instead, formal methoxy-displacement (Johnson–Claisen) and chloride-displacement (Bellus–Claisen) products were isolated amid a complex mixture of products. Interestingly, the application of a Ficini-type Claisen,^19^ using alkyne **6** and either catalytic Au^+^ or TfOH, led to the exclusive formation of the apparent [1,3] shift product **8** (Scheme 3A).^9d^ We were delighted to discover that heating **5** with *N*,*N*-dimethylacetamide dimethylacetal (DMAA)^20^ accomplished the desired Meerwein–Eschenmoser–Claisen rearrangement, producing the tertiary amide **10** cleanly and with remarkable efficiency. Through judicious screening of reaction parameters, we were able to determine conditions that yielded the [3,3] rearrangement product in a 97% yield. The striking success of this variation can be understood by considering the high reactivity of the putative intermediate, ketene *N*,*O*-acetal **9**, which is formed under relatively mild reaction conditions, requiring neither acidic nor basic reagents. The strongly electron-donating dimethylamino group in **9** is thought to increase the highest occupied molecular orbital (HOMO) energy of the alkene, thereby lowering the activation barrier for the [3,3] rearrangement, as observed with related aliphatic rearrangements.^21^ To confirm the assigned constitution of the [3,3] product, **10** was treated with a catalytic amount of trifluoroacetic acid (TFA), which readily isomerized the double bond to give indole **11** in a quantitative yield. The formation of **9** from alcohol **5** is an equilibrium process; thus, to facilitate the conversion to **9** (and ultimately **10**), the methanol byproduct was removed from the reaction vessel via a steady flow of nitrogen or by leaving the system open to the atmosphere. Unfortunately, attempts to lower the reaction temperature by catalyzing it using Lewis or Brønsted acids proved unsuccessful, with a significant number of side products emerging. Best results were obtained when the reaction was performed in toluene at 110 °C with 3 equiv of DMAA (Scheme 3B).

### Examination of Reaction Scope

**Scheme 4:**
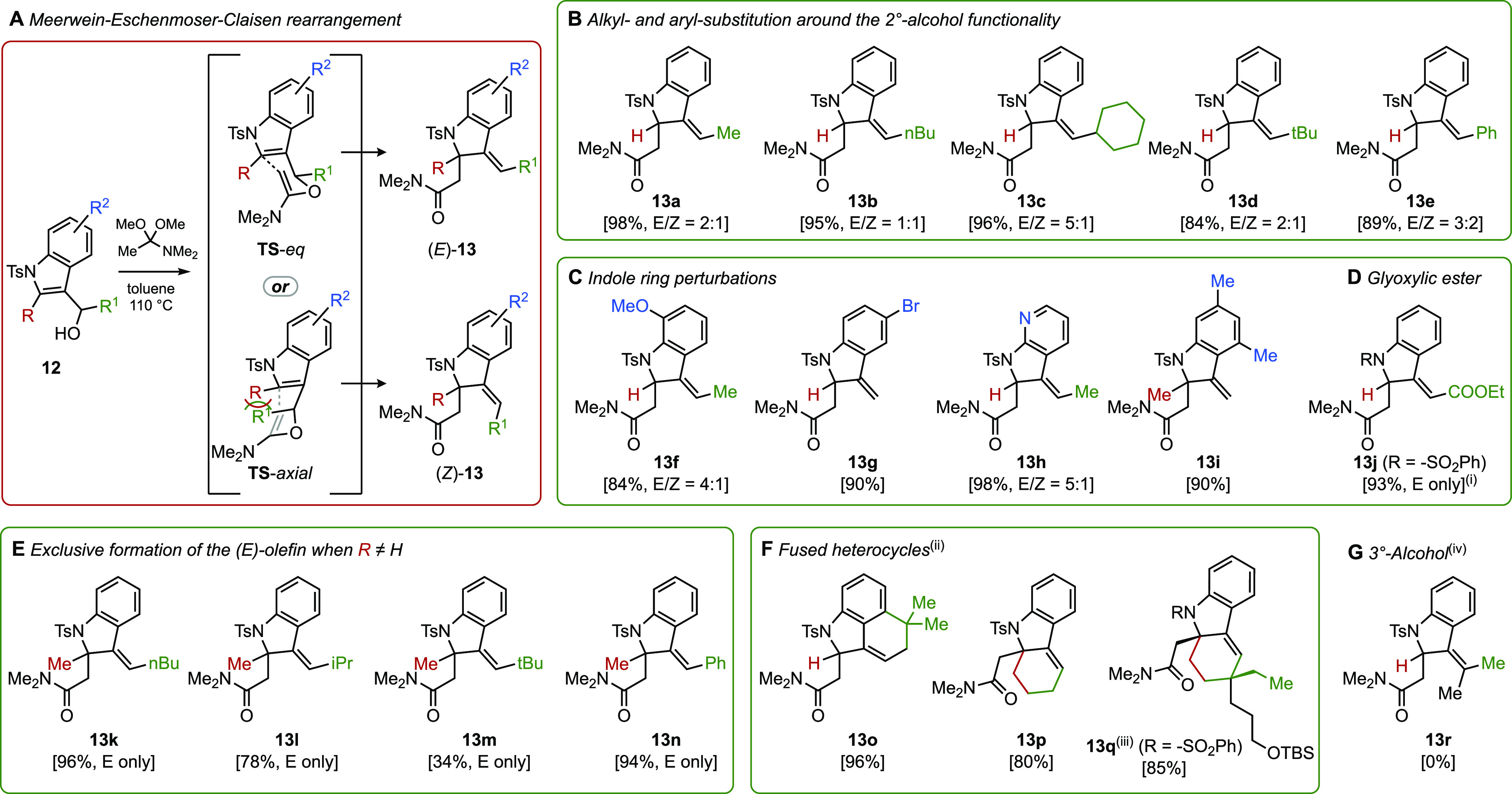
Meerwein–Eschenmoser–Claisen Rearrangement of Structurally Varied 3-Indolyl Alcohols (i) Contains ∼10% of the aromatized product; (ii) μW, 130 °C; (iii) gram-scale; absolute stereochemistry is shown, required 180 °C; and (iv) only dehydrated product was isolated.

With optimal reaction conditions in hand, we turned our attention to exploring the scope of the dearomative rearrangement (Scheme 4). The transformation was found to have a broad substrate scope and to be tolerant of a range of substituents at the R, R^1^, and R^2^ positions of **12**. Substrates that were unsubstituted at C2 (R = H) could possess various alkyl and aryl substituents at the secondary alcohol carbon (R^1^), including bulky groups such as cyclohexyl (**12c**) and *tert*-butyl (**12d**). Perturbations of the benzene ring (**12f–i)**, including the use of 7-azaindole-derived substrate (**12h**), had no untoward effect and afforded the corresponding indoline products (**13f–i**) in good to excellent yields (84–98%), as mixtures of *E*/*Z* isomers, with a preference for *E*-isomers. Interestingly, 3-indoleglyoxylic ester derivative **12j**, a substrate previously utilized by Raucher, rearranged efficiently by employing our protocol to provide amide **13j** in a 93% yield. Of special interest, vis-à-vis using this chemistry for total synthesis objectives such as those mentioned above, are indole substrates bearing substitution at C2, which had proven to be beyond the scope of the Johnson–Claisen method reported by Raucher. We were pleased to find that such substrates underwent the Meerwein–Eschenmoser–Claisen rearrangement nicely to give indolines **13k–n**, having a fully substituted C2 position, in generally high yields (34–96%). Only the substrate in which R^1^ is a *t*-Bu group gave the rearrangement product in a low yield (**13m**). It is noteworthy that substrates possessing a substituent at C2 furnished the rearrangement products exclusively as their *E*-isomers. The high diastereoselectivity observed for these substrates can be understood by considering the chair transition states expected for these rearrangements, wherein the lower-energy conformation (**TS**-eq, Scheme 3A) positions the benzylic substituent in a pseudo-equatorial arrangement, thereby avoiding A^1,3^-strain with the C2 alkyl group. Finally, substrates having an additional ring fused to the indole unit (**12o–q**) also engaged in the title rearrangement to give the corresponding tricycles **13o–q**, though they require harsher reaction conditions. Fused indoles **12o** and **12p** were slow to react under the standard reaction conditions (toluene, 110 °C), and after 8 h, they yielded mainly the elimination product (60%), with the desired product accounting for only 30% of the mass balance. However, the use of microwave irradiation as the heat source (130 °C) effected the rearrangement successfully to provide the desired amides in up to 96% yield. Notably, heating 1.05 g (2 mmol) of the more sophisticated substrate **12q** with 3 equiv of DMAA at 180 °C in mesitylene for 24 h furnished the desired amide **13q** cleanly and in excellent yield, thus demonstrating that the title methodology can be successfully applied in a complex setting. One limitation that has been observed for this rearrangement is with tertiary alcohol substrates. Thus, **12r** yielded none of the desired amide **13r**, giving instead only the dehydration product (not shown).

### Efficacy of Chirality Transfer

**Scheme 5:**
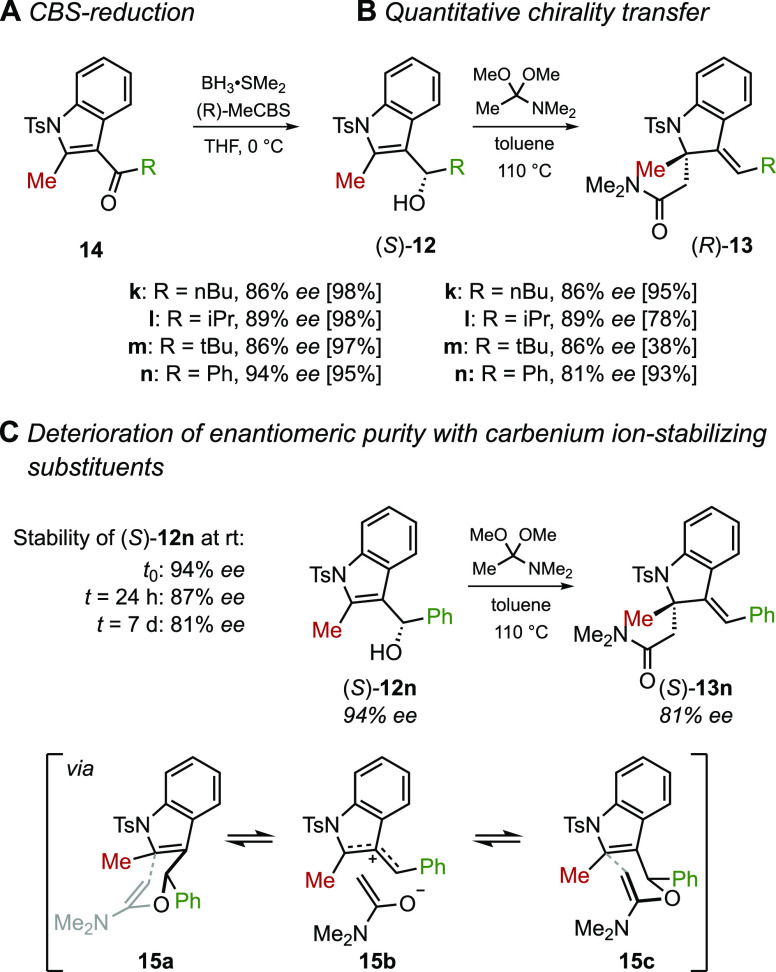
Chirality Transfer Experiments

Having confirmed the broad effectiveness of this dearomative Claisen rearrangement, we next turned our attention to the possibility of using this process to set chirality at the fully substituted C2-carbon of indoline (Scheme 5).^22^ The ordered transition state observed with [3,3] sigmatropic rearrangements provided the potential for high chirality transfer from the benzylic position to the newly formed quaternary center. The required enantiomerically enriched 3-indolyl alcohols (*S*)-**12k-n** were prepared from the respective ketones via a CBS reduction, which proceeded in nearly quantitative yields and gave the needed alcohols in high enantioselectivities (up to 94% ee, Scheme 5A).^23^ When a toluene solution of enantioenriched alcohol (*S*)-**12k** and DMAA was heated, the rearrangement took place in excellent yield as before and transferred the benzylic alcohol chirality to C2 with complete fidelity (Scheme 5B). Comparable results were obtained with the *i-*Pr- and *t-*Bu-substituted 3-indoyl alcohols, although the latter, as with the racemic substrate, proceeded with a lower yield. Curiously, when the phenyl-substituted alcohol **12n** was subjected to the standard reaction conditions, (*R*)-**13n** was obtained with diminished enantiopurity.^24^ Upon closer inspection, it was observed that (*S*)-**12n** racemized gradually just on standing at room temperature on the benchtop. This slow racemization of alcohol 12*n* may be attributed to adventitious moisture or acid, which could catalyze the dissociation of the hydroxyl group to yield a doubly benzylic secondary carbenium ion. Reassociation with water from the opposite face would explain the observed erosion in *ee*. This line of reasoning suggests that at the temperature used for the rearrangement the *N*,*O*-acetal intermediate **15a** may suffer a similar fate and furnish the rearranged tertiary amide with diminished enantiomeric excess (Scheme 5C).

The title dearomative Claisen rearrangement offers a reliable path to both enantiomers of indolines bearing a fully substituted C2 since either enantiomer of **12** can be prepared simply by choosing the appropriate enantiomer of the oxazaborolidine catalyst. Additionally, enantioenriched 3-indolyl alcohols are also readily prepared by other methods, such as through asymmetric Friedel–Crafts reactions,^25^ thereby further augmenting the utility of the Claisen methodology.

### Application of Indolic Claisen to Enantioselective Total Synthesis of (+)-Hinckdentine A

The true capability of a new synthetic method is best appraised by assessing its performance in a complex setting that could also help define its limitations. The promising results of our extensive studies on the scope of this [3,3] rearrangement—giving generally high yields, broad substrate scope, and reliable transfer of stereochemical information—portended well for its capacity to solve the key structural challenges of the intricate natural products listed in Scheme 1. With that in mind, we set out to tackle the enantioselective total synthesis of the pentacyclic alkaloid hinckdentine A (**1**), utilizing the dearomative Claisen rearrangement as a pivotal step.

**Scheme 6:**
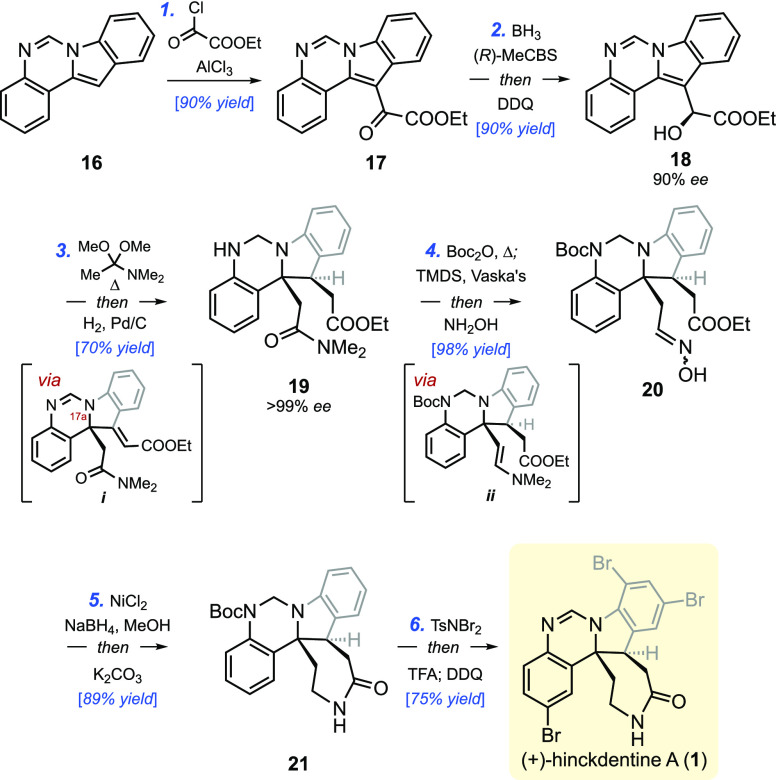
Concise, Enantioselective Total Synthesis of (+)-Hinckdentine A Reagents and conditions: (a) AlCl_3_ (3 equiv), ethyl chlorooxoacetate (3 equiv), CH_2_Cl_2_, 0 °C, 2 h; (b) (*R*)-MeCBS (50 mol%), BH_3_·SMe_2_ (5 equiv), CH_2_Cl_2_/ether = 1:9, 0 °C, 20 min; then DDQ (1.1 equiv), MeOH, rt, 10 min; (c) DMAA (3 equiv), toluene, 120 °C, 16 h; then H_2_ (balloon), Pd/C (20 mol%), 60 °C, 15 h; (d) Boc_2_O (2 equiv), toluene, 120 °C, 12 h; then IrCl(CO)(PPh_3_)_2_ (1 mol%), TMDS (3 equiv), rt, 5 min; then NH_2_OH·HCl (1.5 equiv), MeOH, rt, 20 min; (e) NiCl_2_·DME (2 equiv), NaBH_4_ (10 equiv), MeOH, rt, 1 h; then K_2_CO_3_ (20 equiv), rt, 20 h; (f) TsNBr_2_ (3 equiv), CH_2_Cl_2_, −30 °C, 2 h; then anisole (10 equiv), −30 to 0 °C, 15 min; then TFA, 0 °C to rt, 1 h; then DDQ (1.2 equiv), 10 min.

Isolated in 1987 from marine bryozoan *Hincksinoflustra denticulate*,^1^ hinckdentine A possesses a highly brominated indolo[1,2-*c*]quinazoline core and a 7-membered lactam unit fused to the indoline moiety through two contiguous stereogenic centers.^26,27^ Among the challenges presented by hinckdentine A is the stereocontrolled construction of the fully substituted indoline C2 position and the selective tribromination of the two benzene rings. Our aim in undertaking the synthesis was to resolve the fundamental structural issue through the indolic Meerwein–Eschenmoser–Claisen rearrangement and to do so via a succinct pathway that was distinct from the documented routes.^26^ With the objective of achieving an asymmetric synthesis of hinckdentine A, indolic alcohol **18**, required for the pivotal Claisen rearrangement, was prepared from the known indoloquinazoline **16**, which was synthesized on decagram scale from 2-aminoacetophenone (Scheme 6).^27a,28,29^ Friedel–Crafts glyoxylation of **16** followed by CBS reduction of the ketone and DDQ addition to reform the amidine afforded indolic alcohol **18** in an 81% yield and 90% ee. Despite its structural and steric complexity, and the presence of an unexplored functional group attached to the indole nitrogen, alcohol **18** underwent a clean Claisen rearrangement upon heating with 3 equiv of DMAA in toluene to afford the desired product, thereby installing the required two-carbon unit at C17a (hinckdentine A numbering). Hydrogenation of the alkene intermediate (***i***), which was formed exclusively as its *E* diastereomer, was accomplished in the same reaction vessel by simply adding Pd/C catalyst and attaching a hydrogen balloon to the flask. This single operation installed the two stereogenic centers of the natural product with complete selectivity; moreover, crystallization of the product from acetone/hexane mixture afforded (+)-**19** as a single enantiomer in a 70% yield from alcohol **18**.

The major challenge that followed was the chemoselective reduction of the amide carbonyl and replacement of the dimethylamine unit with −NH_2_ to form the desired δ-lactam. This task posed a difficulty, as the use of several common reducing agents opened up undedsired side-reactions, including the reduction of more than one carbonyl functionality, the reductive removal of the Boc group, the retro-Mannich reaction resulting in the loss of the two-carbon unit introduced via Claisen rearrangement, and the degradation of the aminal functionality. In the end, Vaska’s catalyst provided the ideal solution to the problem, as evidenced by the successful synthesis of the desired oxime **20** in a 98% yield. In preparation for its use, the secondary amine of **19** was blocked with a Boc group, and the reaction mixture was then treated directly with IrCl(CO)(PPh_3_)_2_/TMDS,^30^ which cleanly furnished an enamine intermediate (***ii***) that upon treatment with methanolic NH_2_OH·HCl produced the desired oxime **20**. Chemoselective reduction of the oxime functionality to the primary amine followed by the addition of K_2_CO_3_ furnished caprolactam **21**, previously attained by Kawasaki.^26a^ Tribromination of advanced intermediates such as **21** had been a major impediment in prior syntheses of hinckdentine A. An extensive screening of brominating agents and reaction conditions using caprolactam **21** as a substrate revealed *N*,*N*-dibromo-*p*-toluenesulfonamide (TsNBr_2_)^31^ to be superior to all other brominating agents examined.^32^ This reagent efficiently returned the desired tribromo product, which upon treatment in the same flask with TFA followed by DDQ afforded (+)-hinckdentine A in a 75% yield.

## Conclusions

To summarize, we have developed a powerful synthetic protocol for introducing a carbon fragment to the C2 position of indolines via the dearomative Meerwein–Eschenmoser–Claisen rearrangement of 3-indolyl alcohols. The rearrangement proceeds in good to excellent yields and offers a general method for the installation of a fully substituted carbon at the C2 position. High chirality transfer is observed with enantiomerically enriched alcohol precursors, which are readily obtained through asymmetric reduction of the corresponding ketones. The power of this [3,3] rearrangement is demonstrated through a concise, enantioselective total synthesis of (+)-hinckdentine A, wherein the rearrangement installs the requisite two-carbon fragment at the C2 position of the indoline. Other noteworthy steps in the synthesis include (1) diastereocontrolled hydrogenation of the alkene in the rearrangement product, (2) chemoselective amide-to-oxime conversion using Vaska’s complex, and (3) regioselective tribromination of caprolactam **21**. The study of related dearomative Claisen rearrangements is expected to expand access to intricate frameworks found in natural products and complex molecules of biomedical interest.

## Supporting Information Available

Experimental procedures and spectroscopic data and HPLC analyses for all new compounds (PDF)
