# Supplementary material for: Rapid Access to 2,2-Disubstituted Indolines via Dearomative Indolic-Claisen Rearrangement: Concise, Enantioselective Total Synthesis of (+)-Hinckdentine A
Source: J Am Chem Soc. 2023 Jun 26;145(27):14831–8. doi: 10.1021/jacs.3c03611 (PMC10347551; doi:10.1021/jacs.3c03611)
Supplement: Supplementary file 1 — ja3c03611_si_001.pdf [file ja3c03611_si_001.pdf]

JACS
JOURNAL OF THE AMERICAN CHEMICAL SOCIETY

Rapid Access to 2,2–Disubstituted Indolines via Dearomative Indolic-Claisen Rearrangement: Concise, Enantioselective Total Synthesis of (+)-Hinckdentine A

Daler Baidilov, Pavel K. Elkin, Sudhakar Athe, and Viresh H. Rawal*

Department of Chemistry
The University of Chicago
5735 South Ellis Avenue, Chicago, Illinois 60637
E-mail: vrawal@uchicago.edu

Table of Contents

General Remarks 3

Section 1: Indole-Claisen Rearrangement 4

Preliminary Studies of [3,3]-Rearrangement 4

General Experimental Procedure: Substrate Scope 5

Gram-Scale Application 8

Synthesis of Enatioenriched Alcohols 9

Chirality Transfer Experiments 11

Section 2: Hinckdentine A 13

Synthesis of Starting Material 13

Total Synthesis of (+)-Hinckdentine A 14

Bromination 17

Section 3: NMR Spectra 18

Appendix 78

Kawasaki's synthesis of *rac*-hinckdentine A (2009) 78

Fukuyama's synthesis of (+)-hinckdentine A (2016) 78

Zhu's synthesis of (+)-hinckdentine A (2018) 78

Xu's synthesis of *rac*-hinckdentine A (2018) 79

Cheon's synthesis of *rac*-hinckdentine A (2021) 79

Hong's synthesis of (+)-hinckdentine A (2022) 79

Summary 80

General Remarks

All reactions were performed in oven-dried (>12 h at 120 °C) and/or flame-dried glassware equipped with a Teflon-coated magnetic stir bar under an atmosphere of nitrogen which had been pre-dried by passage through a Drierite® column (CaSO4 ≥ 98% + $CoCl_2$ <2%) unless otherwise specified. Reaction solvents dichloromethane (CH_2Cl_2; unstabilized HPLC grade), tetrahydrofuran (THF; HPLC grade), toluene (PhMe; ACS grade), and diethyl ether (Et_2O; ACS grade, stabilized with BHT) were dried by passage through an activated alumina column purification system (Innovative Technology Inc. Pure-SolvTM). Anhydrous methanol (MeOH), ethanol (EtOH), 2-propanol (i-PrOH), and benzene (PhH) were purchased from Sigma-Aldrich and used as received. Anhydrous acetonitrile (MeCN) and N,N-dimethylformamide (DMF) were purchased from Sigma Aldrich and used as received. Commercially obtained reagents were used as received, unless stated otherwise.

Room temperature refers to 22 °C. Higher temperatures were maintained using pre-heated oil baths; oil bath temperatures are reported. Lower temperatures were maintained using a cooling bath of acetone/dry ice (-78 °C), water/ice (0 °C), or NESLAB CB-80 Cryobath for all other temperatures in between. Similarly, reported temperature values are those of cooling baths.

Thin-layer chromatography (TLC) was performed using EMD Millipore silica gel 60 Å plates and visualization was achieved with either UV fluorescence quenching (254 nm), Hanessian's Stain (Cerium Ammonium Molybdate) with heat, or Seebach's stain ($Ce(SO_4)_2$ in phosphomolybdic acid) with heat. Flash column chromatography was performed on SiliCycle SiliaFlash P60 (40-63 µm particle size) using ACS grade solvents purchased from Fisher Scientific. Hexanes were freshly distilled prior to use to minimize *H*-grease content.

Nuclear magnetic resonance (NMR) data were acquired on 500 MHz Bruker Avance-IIIHD spectrometer equipped with a BBFO SmartProbe, using Topspin 3.6.2. ^{1}H NMR spectra were calibrated from solvent residual peak (e.g., residual $CHCl_3$ in chloroform-*d*: 7.26 ppm) resonances and ^{13}C NMR spectra from solvent (e.g., chloroform-*d*: 77.16 ppm). Chemical shifts (δ) are reported in parts per million (ppm) relative to the residual solvent resonance and coupling constants (*J*) are reported in hertz (Hz). NMR peak pattern abbreviations are as follows: s = singlet, d = doublet, t = triplet, q = quartet, pent = pentet, sept = septet, dd = doublet of doublets, dt = doublet of triplets, td = triplet of doublets, tt = triplet of triplets, qd = quarter of doublets, ddd = doublet of doublet of doublets, ddt = doublet of doublet of triplets, tdd = triplet of doublet of doublets, m = multiplet, br = broad (i.e., signal is broadened), app = apparent (i.e., signal appears as). All non-trivial ^{1}H- and ^{13}C-spectra are corroborated by 2-D experiments (e.g., COSY, HSQC, HMBC, NOESY).

High-resolution mass spectral (HRMS) analyses were performed on Agilent Technologies 6224 TOF LC/MS using electrospray ionization (ESI) at the University of Chicago Mass Spectroscopy Core Facility. Optical rotations were measured on a Jasco DIP-1000 polarimeter using a 100 mm-path-length cell, c = g/100 mL. Infrared (IR) spectra were recorded on a Thermo Scientific Nicolet iS50 FT-IR spectrometer and are reported as a frequency of absorption (cm^{-1}). Chiral high-performance liquid chromatography (HPLC) analyses were performed using an Agilent analytical chromatography system with commercial Chiralcel® columns equipped with a guard column.

Section 1: Indole-Claisen Rearrangement

Preliminary Studies of [3,3]-Rearrangement

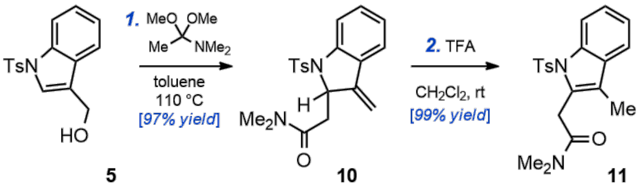

5 10 11

[1] Indole-Claisen

To a stirred mixture of indolyl alcohol **5** (150 mg, 0.5 mmol) and toluene (4 mL) was added *N*,*N*-dimethylacetamide dimethylacetal (DMMA, 220 µl, 1.5 mmol, 3 equiv) at room temperature. The mixture was degassed by bubbling Ar for 10 min, then placed in an oil bath pre-heated to 110 °C. After heating for 1 h, the mixture was concentrated under reduced pressure, providing the crude product, which was purified using silica gel column chromatography (EtOAc/hexanes = 1:9 to 1:1) affording indoline **10** (179 mg, 97% yield) as a pale-yellow oil.

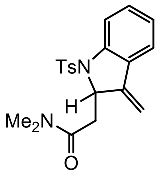

10

10: ^{1}H NMR (500 MHz, $CDCl_3$) δ 7.78 (d, *J* = 8.2 Hz, 1H), 7.60 (d, *J* = 8.3 Hz, 2H), 7.33 – 7.25 (m, 2H), 7.15 (d, *J* = 8.3 Hz, 2H), 7.05 (d, *J* = 7.5 Hz, 1H), 5.33 (d, *J* = 2.5 Hz, 1H), 5.23 – 5.17 (m, 1H), 5.14 (d, *J* = 2.5 Hz, 1H), 3.24 (dd, *J* = 15.9, 3.3 Hz, 1H), 3.01 (s, 3H), 2.99 (s, 3H), 2.77 (dd, J = 15.9, 8.9 Hz, 1H), 2.32 (s, 3H); ^{13}C NMR (125 MHz, $CDCl_3$) δ 169.6, 145.2, 144.1, 143.2, 129.9, 129.6, 129.5, 127.3, 124.4, 120.9, 116.5, 104.4, 62.9, 41.9, 37.2, 35.4, 21.4; IR (neat, cm^{-1}): 2927 (br), 1645, 1598, 1461, 1355, 1168, 757, 664, 581; HRMS (ES) calc'd for $C_{20}H_{23}N_2O_3S$ $[M+H]^+$: 371.1429, found 371.1415.

[2] Olefin Isomerization

To a stirred solution of indoline **10** (80 mg, 0.2 mmol) in CH_2Cl_2 (3 mL) was added a drop of TFA at room temperature. After stirring for 1 h, the mixture was concentrated under reduced pressure to provide the crude product, which was purified using silica gel column chromatography (EtOAc/hexanes = 1:19 to 3:7) to furnish indole **11** (79 mg, 99% yield) as a pale-yellow oil.

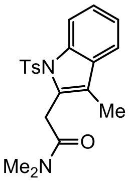

11

11: ^{1}H NMR (500 MHz, CDCl3) δ 7.93 – 7.85 (m, 1H), 7.73 (d, *J* = 8.1 Hz, 2H), 7.43 – 7.36 (m, 1H), 7.23 – 7.17 (m, 2H), 7.15 (d, *J* = 8.1 Hz, 2H), 4.11 (s, 2H), 3.18 (s, 3H), 3.00 (s, 3H), 2.30 (s, 3H), 2.18 (s, 3H); ^{13}C NMR (125 MHz, $CDCl_3$) 169.1, 144.4, 135.9, 131.0, 130.8, 129.5, 127.0, 124.1, 123.1, 119.3, 118.6, 114.4, 37.5, 35.8, 31.3, 21.5, 9.0; IR (neat, cm^{-1}): 2923 (br), 1652, 1454, 1397,1310,1170,1152, 1126, 666, 585; HRMS (ES) calc'd for $C_{20}H_{23}N_2O_3S$ $[M+H]^+$: 371.1429, found 371.1429.

JACS JOURNAL OF THE AMERICAN CHEMICAL SOCIETY

General Experimental Procedure: Substrate Scope

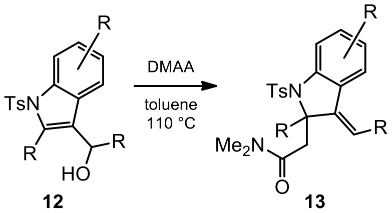

12 **13**

To a stirred mixture of indolyl alcohol **12** (0.5 mmol) and toluene (4 mL) was added DMMA (0.22 mL, 1.5 mmol, 3 equiv) at room temperature. The mixture was degassed by bubbling Ar for 10 min, then placed in oil bath pre-heated to 110 °C. When TLC indicated a complete consumption of the starting material (1-4 h), the mixture was concentrated under reduced pressure to provide a crude material, which was purified using silica gel column chromatography (mobile phase: EtOAc/hexanes) to afford the [3,3]-product **13**.

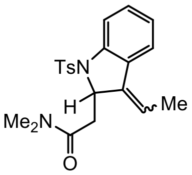

13a
[E/Z = 2:1]

13a: yellow foam (188 mg, 98% yield); ^{1}H NMR (500 MHz, $CDCl_3$) *major* δ 7.78 (d, *J* = 8.2 Hz, 1H), 7.57 (d, *J* = 8.3 Hz, 2H), 7.42 (d, *J* = 7.6 Hz, 1H), 7.25 – 7.20 (m, 1H), 7.15 (d, *J* = 8.3 Hz, 2H), 7.07 (td, *J* = 7.8, 1.5 Hz, 2H), 5.72 (qd, *J* = 7.4, 1.7 Hz, 1H), 5.13 (ddt, *J* = 8.4, 3.5, 1.7 Hz, 1H), 3.13 (dd, *J* = 15.7, 3.6 Hz, 1H), 3.00 (s, 3H), 2.98 (s, 3H), 2.71 (dd, *J* = 15.7, 8.5 Hz, 1H), 2.32 (s, 3H), 1.84 (dd, *J* = 7.4, 1.7 Hz, 3H), *minor* δ 7.72 (dd, *J* = 8.4, 1.1 Hz, 1H), 7.53 (d, *J* = 8.4 Hz, 2H), 7.26 – 7.20 (m, 2H), 7.12 (d, *J* = 8.4 Hz, 2H), 7.04 (td, *J* = 7.6, 1.0 Hz, 1H), 5.79 (qd, *J* = 7.3, 2.2 Hz, 1H), 5.28 – 5.23 (m, 1H), 3.09 (s, 3H), 3.00 (dd, *J* = 14.5, 6.8 Hz, 1H), 2.98 (s, 3H), 2.64 (dd, *J* = 14.5, 4.5 Hz, 1H), 2.31 (s, 3H), 1.75 (dd, *J* = 7.3, 1.3 Hz, 3H); ^{13}C NMR (125 MHz, $CDCl_3$) *mixture* δ 169.8, 169.6, 143.9, 143.8, 143.6, 142.0, 137.5, 136.9, 134.0, 131.1, 130.0, 129.8, 129.5, 129.4, 128.7, 128.6, 127.2, 127.1, 126.8, 124.9, 124.8, 124.3, 119.9, 119.7, 117.3, 116.6, 116.4, 63.5, 62.2, 42.5, 40.1, 38.0, 37.3, 35.6, 35.4, 21.4, 14.4, 14.2; IR (neat, cm^{-1}): 2925 (br), 1644, 1597, 1459, 1400, 1355, 1160, 1091, 753, 733, 665; HRMS (ES) calc'd for $C_{21}H_{25}N_2O_3S$ $[M+H]^+$: 385.1586, found 385.1572.

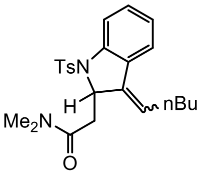

13b
[E/Z = 1:1]

13b: yellow foam (202 mg, 95% yield); ^{1}H NMR (500 MHz, $CDCl_3$) *mixture* δ 7.77 (d, *J* = 8.1 Hz, 0.5H), 7.73 (d, *J* = 7.9 Hz, 0.5H), 7.55 (d, *J* = 8.4 Hz, 1H), 7.50 (d, *J* = 8.3 Hz, 1H), 7.41 (d, *J* = 7.7 Hz, 0.5H), 7.27 – 7.20 (m, 1.5H), 7.16 – 7.10 (m, 2H), 7.09 – 7.04 (m, 1H), 5.67 (ddd, *J* = 9.0, 7.2, 2.1 Hz, 0.5H), 5.57 (ddd, *J* = 7.3, 7.3, 1.7 Hz, 0.5H), 5.20 – 5.15 (m, 1H), 5.14 – 5.09 (m, 1H), 3.09 (s, 1.5H), 3.00 – 2.96 (m, 0.5 H), 3.08 – 3.03 (m, 1H)2.99 (s, 1.5H), 2.98 (s, 3H), 2.69 (dd, *J* = 15.3, 8.3 Hz, 0.5H), 2.58 (dd, *J* = 14.4, 4.4 Hz, 0.5H), 2.32 (s, 3H), 2.28 – 2.20 (m, 1H), 2.15 – 2.07 (m, 1H), 1.41 – 1.11 (m, 4H), 0.85 (q, *J* = 7.1 Hz, 3H); ^{13}C NMR (125 MHz, $CDCl_3$) *mixture* δ 169.8, 169.5, 143.8, 143.8, 143.7, 142.1, 136.6, 135.6, 134.2, 131.3, 130.0, 129.5, 129.4, 128.7, 128.6, 127.4, 127.3, 127.2, 127.1, 126.7, 126.5, 125.0, 124.8, 124.6, 122.4, 120.0, 117.6, 117.1, 64.0, 62.5, 42.2, 40.5, 38.1, 37.5, 35.6, 35.4, 31.5, 31.4, 28.5, 27.9, 22.2, 22.1, 21.4, 13.9, 13.8; IR (neat, cm^{-1}): 2927 (br), 1646, 1459, 1399, 1306, 1212, 1168, 1108, 1091, 1042, 964, 814, 755, 665; HRMS (ES) calc'd for $C_{24}H_{21}N_2O_3S$ $[M+H]^+$: 427.2055, found 427.2054.

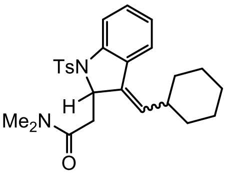

13c
[E/Z = 5:1]

13c: yellow foam (216 mg, 96% yield); ^{1}H NMR (500 MHz, $CDCl_3$) *major* δ 7.76 (d, *J* = 8.2 Hz, 1H), 7.53 (d, *J* = 8.3 Hz, 2H), 7.36 (d, *J* = 7.4 Hz, 1H), 7.27 – 7.23 (m, 1H), 7.12 (d, *J* = 8.3 Hz, 2H), 7.08 (td, *J* = 7.6, 1.1 Hz, 1H), 5.38 (dd, *J* = 9.3, 1.6 Hz, 1H), 5.06 (ddd, *J* = 8.3, 4.6, 1.7 Hz, 1H), 3.04 (dd, *J* = 14.9, 4.6 Hz, 1H), 2.98 (s, 6H), 2.66 (dd, *J* = 14.9, 8.3 Hz, 1H), 2.53 – 2.42 (m, 1H), 2.31 (s, 3H), 1.78 – 0.84 (m, 10H), *minor* δ 7.70 (dd, *J* = 8.6, 1.1 Hz, 1H), 7.49 (d, *J* = 8.3 Hz, 2H), 7.27 – 7.20 (m, 2H), 7.12 (d, *J* = 8.3 Hz, 2H), 7.06 (td, *J* = 7.7, 1.0 Hz, 1H), 5.51 (dd, *J* = 10.5, 2.0 Hz, 1H), 5.20 (ddd, *J* = 7.3, 4.4, 2.0 Hz, 1H), 3.09 (s, 3H), 2.99 (s, 3H), 2.98 (dd, *J* = 14.3, 7.4 Hz, 1H), 2.55 (dd, *J* = 14.3, 4.4 Hz, 1H), 2.55 (dd, *J* = 14.9, 4.6 Hz, 1H), 2.53 – 2.42 (m, 1H), 2.30 (s, 3H), 1.78 – 0.84 (m, 10H); ^{13}C NMR (125 MHz, $CDCl_3$) *mixture* δ 169.6, 143.8, 133.9, 131.9, 129.6, 129.4, 129.3, 128.6, 127.2, 127.1, 124.7, 124.5, 117.4, 64.2, 41.9, 37.6, 36.6, 35.4, 32.3, 25.8, 25.6, 25.5, 21.3; IR (neat, cm^{-1}): 2924 (br), 1653, 1596, 1505, 1456, 1356, 1168, 763, 665; HRMS (ES) calc'd for $C_{26}H_{33}N_2O_3S$ $[M+H]^+$: 453.2212, found 453.2206.

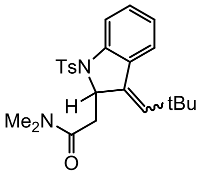

13d
[E/Z = 2:1]

13d: yellow foam (179 mg, 84%); ^{1}H NMR (500 MHz, $CDCl_3$) *mixture* δ 7.74 (d, *J* = 8.4 Hz, 0.5H), 7.72 (d, *J* = 8.4 Hz, 1H), 7.52 (d, *J* = 7.8 Hz, 0.5H), 7.47 (d, *J* = 8.3 Hz, 1H), 7.40 (d, *J* = 8.3 Hz, 2H), 7.28 – 7.21 (m, 3H), 7.15 – 7.07 (m, 4H), 5.59 (d, *J* = 1.8 Hz, 1H), 5.54 (d, *J* = 1.5 Hz, 0.5H), 5.39 (ddd, *J* = 9.6, 2.2, 2.2 Hz, 1H), 4.94 (ddd, *J* = 7.3, 5.7, 1.5 Hz, 0.5H), 3.09 (s, 3H), 3.00 (s, 3H), 2.98 (s, 1.5H), 2.97 (s, 1.5H), 2.96 (dd, *J* = 14.3, 5.9 Hz, 0.5H), 2.89 (dd, *J* = 13.7, 9.7 Hz, 1H), 2.59 (dd, *J* = 14.3, 7.6 Hz, 0.5H), 2.46 (dd, *J* = 13.8, 2.5 Hz, 1H), 2.32 (s, 1.5H), 2.31 (s, 3H), 1.08 (s, 9H), 1.04 (s, 4.5H); ^{13}C NMR (125 MHz, $CDCl_3$) *mixture* δ 169.6, 143.8, 140.7, 137.4, 133.9, 133.1, 132.7, 129.3, 128.7, 128.7, 127.4, 127.3, 127.3, 125.6, 124.7, 120.0, 119.0, 118.9, 66.8, 62.7, 41.6, 40.5, 38.4, 37.9, 35.6, 35.5, 33.9, 31.7, 30.4, 29.8, 21.4; IR (neat, cm^{-1}): 2954 (br), 1717, 1645, 1357, 1169, 667, 578; HRMS (ES) calc'd for $C_{24}H_{31}N_2O_3S$ $[M+H]^+$: 427.2055, found 427.2048.

JACS
JOURNAL OF THE AMERICAN CHEMICAL SOCIETY

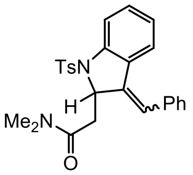

13e
[E/Z = 3:2]

13e: yellow foam (198 mg, 89% yield); ^{1}H NMR (500 MHz, $CDCl_3$) *mixture* δ 7.83 – 7.74 (d, *J* = 8.2 Hz, 1H), 7.64 – 7.52 (d, *J* = 8.3, 2H), 7.48 (d, *J* = 7.7 Hz, 1H), 7.44 – 6.76 (m, 9H), 6.69 (d, *J* = 2.3 Hz, 0.4H), 6.63 (s, 0.6H), 5.68 (ddd, *J* = 8.2, 2.6, 2.6 Hz, 0.4H), 5.33 – 5.25 (m, 0.6H), 3.22 (dd, *J* = 15.6, 3.9 Hz, 0.6H), 3.03 (s, 1.8H), 3.02 (s, 1.2H), 3.00 (s, 1.8H), 2.95 (s, 1.2H), 2.90 (dd, *J* = 14.6, 8.1 Hz, 0.4H), 2.84 (dd, *J* = 15.6, 8.3 Hz, 0.6H), 2.60 (dd, J = 14.6, 2.8 Hz, 0.4H), 2.33 (s, 1.8H), 2.26 (s, 1.2H); ^{13}C NMR (125 MHz, $CDCl_3$) *mixture* δ 169.7, 169.6, 144.3, 144.1, 144.0, 142.2, 138.2, 137.4, 136.6, 135.1, 133.7, 132.2, 129.6, 129.5, 128.8, 128.7, 128.6, 128.5, 128.3, 128.2, 127.5, 127.4, 127.3, 127.2, 126.7, 125.1, 124.2, 124.1, 123.7, 120.1, 119.8, 117.7, 117.2, 64.1, 63.4, 42.3, 37.9, 37.8, 37.4, 35.6, 35.5, 21.4, 21.4; IR (neat,cm^{-1}): 3053 (br), 2926 (br), 1644, 1596, 1493, 1400, 1357, 1169, 1107, 763, 733, 663; HRMS (ES) calc'd for $C_{26}H_{27}N_2O_3S$ $[M+H]^+$: 447.1742, found 447.1739.

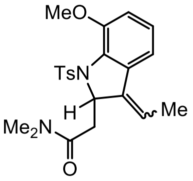

13f
[E/Z = 4:1]

13f: yellow foam (173 mg, 84% yield); ^{1}H NMR (500 MHz, $CDCl_3$) *mixture* δ 7.50 – 7.44 (m, 2.5H), 7.18 – 7.11 (m, 3.25H), 7.10 (d, *J* = 8.2 Hz, 0.25H) 7.02 (d, *J* = 7.7 Hz, 1H), 6.89 (d, *J* = 8.3 Hz, 1H), 6.84 (d, *J* = 8.2 Hz, 0.25H), 6.83 (dd, *J* = 7.6, 1.0 Hz, 0.25H), 5.65 (qd, *J* = 7.2, 1.7 Hz, 0.25H), 5.56 (qd, *J* = 7.3, 1.2 Hz, 1H), 5.43 – 5.38 (m, 0.25H), 5.21 – 5.16 (m, 1H), 3.89 (s, 3H), 3.87 (s, 0.75H), 2.98 (s, 0.75H), 2.90 (s, 0.75H), 2.89 (s, 3H), 2.88 (s, 3H), 2.81 (dd, *J* = 15.0, 5.8 Hz, 1H), 2.76 (dd, *J* = 13.9, 8.2 Hz, 0.25H), 2.47 (dd, *J* = 15.0, 8.2 Hz, 1H), 2.37 (dd, *J* = 13.9, 5.2 Hz, 0.25H), 2.36 (s, 0.75H), 2.35 (s, 3H), 1.67 (d, *J* = 7.3 Hz, 3H), 1.65 (d, *J* = 7.2 Hz, 0.75H); ^{13}C NMR (125 MHz, $CDCl_3$) 169.5, 152.9, 143.4, 137.0, 135.2, 134.8, 132.6, 128.9, 128.1, 127.9, 127.7, 127.6, 121.4, 117.4, 112.9, 112.7, 66.9, 56.1, 40.3, 37.5, 35.4, 21.4, 13.9; IR (neat, cm^{-1}): 2937 (br), 1644,1598, 1488, 1438,1399, 1357, 1276, 1160, 1066; HRMS (ES) calc'd for $C_{22}H_{27}N_2O_4S$ $[M+H]^+$: 415.1692, found 415.1689.

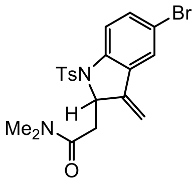

13g

13g: white foam (201 mg, 90 % yield); ^{1}H NMR (500 MHz, $CDCl_3$) δ 7.66 (d, *J* = 8.6 Hz, 1H), 7.59 (d, *J* = 8.3 Hz, 2H), 7.40 (d, *J* = 2.0 Hz, 1H), 7.37 (dd, *J* = 8.6, 2.0 Hz, 2H), 7.20 (d, *J* = 8.0 Hz, 2H), 5.33 (dd, *J* = 3.2, 1.8 Hz, 1H), 5.18 (d, *J* = 1.8 Hz, 1H), 5.21 – 5.14 (m, 1H), 3.21 (dd, *J* = 15.9, 2.9 Hz, 1H), 3.01 (s, 3H), 2.99 (s, 3H), 2.79 (dd, *J* = 16.2, 9.2 Hz, 1H), 2.35 (s, 3H); ^{13}C NMR (125 MHz, $CDCl_3$) δ 169.5, 144.5, 144.0, 142.4, 132.6, 131.7, 129.8, 127.3, 123.9, 117.9, 117.6, 105.9, 63.2, 41.8, 37.3, 35.5, 21.5; IR (neat, cm^{-1}): 2925 (br), 1645, 1459, 1357, 1166, 666; HRMS (ES) calc'd for $C_{20}H_{22}BrN_2O_3S$ $[M+H]^+$: 449.0535, found 449.0525.

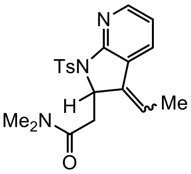

13h
[E/Z = 5:1]

13h: white foam (189 mg, 98% yield); ^{1}H NMR (500 MHz, $CDCl_3$) *major* δ 8.19 (d, *J* = 5.1 Hz, 1H), 7.89 (d, *J* = 8.3 Hz, 2H), 7.67 (d, *J* = 7.0 Hz, 1H), 7.21 (d, *J* = 8.1 Hz, 2H), 6.90 (dd, *J* = 7.7, 5.1 Hz, 1H), 5.95 (dd, *J* = 7.1, 6.3 Hz, 1H), 5.38 (d, J = 8.4 Hz, 1H), 3.27 (dd, J = 15.9, 2.9 Hz, 1H), 3.02 (s, 3H), 2.98 (s, 3H), 2.81 (dd, *J* = 15.9, 8.6 Hz, 1H), 2.35 (s, 3H), 1.90 (dd, *J* = 7.4, 1.9 Hz, 3H), *minor* δ 7.98 (d, *J* = 7.9 Hz, 1H), 7.86 (d, *J* = 8.4 Hz, 2H), 7.49 (d, *J* = 7.7 Hz, 1H), 7.21 (d, *J* = 8.4 Hz, 2H), 6.88 (dd, *J* = 7.9, 7.7 Hz, 1H), 5.95 (m, 1H), 5.49 (m, 1H), 3.09 (dd, *J* = 15.7, 5.1 Hz, 1H), 2.94 (s, 3H), 2.91 (s, 3H), 2.87 (dd, *J* = 15.7, 5.2 Hz, 2H), 2.35 (s, 3H), 1.84 (dd, J = 7.4, 1.5 Hz, 3H); ^{13}C NMR (125 MHz, $CDCl_3$) *mixture* δ 169.6, 156.9, 147.8, 147.4, 144.1, 135.7, 134.5, 132.4, 129.4, 127.9, 127.6, 122.7, 119.0, 119.0, 118.6, 62.1, 42.6, 37.3, 35.5, 21.5, 14.5; IR (neat, cm^{-1}): 2928 (br), 1647, 1596, 1407, 1361, 1172, 1090, 664; HRMS (ES) calc'd for $C_{20}H_{24}N_3O_3S$ $[M+H]^+$: 386.1538, found 386.1526.

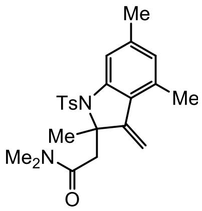

13i

13i: white foam (185 mg, 90% yield); ^{1}H NMR (500 MHz, $CDCl_3$) δ 7.78 (d, *J* = 8.1 Hz, 2H), 7.24 (s, 1H), 7.19 (d, *J* = 8.1 Hz, 2H), 6.61 (s, 1H), 5.35 (d, *J* = 1.2 Hz, 1H), 4.85 (d, *J* = 1.2 Hz, 1H), 3.56 (d, *J* = 15.9 Hz, 1H), 2.94 (s, 3H), 2.83 (d, *J* = 15.9 Hz, 1H), 2.72 (s, 3H), 2.39 (s, 3H), 2.34 (s, 3H), 2.26 (s, 3H), 1.72 (s, 3H); ^{13}C NMR (125 MHz, $CDCl_3$) δ 168.5, 152.0, 143.9, 143.4, 139.3, 134.9, 129.5, 126.7, 122.9, 112.2, 102.7, 72.5, 43.6, 37.6, 35.3, 29.0, 21.9, 21.5, 21.4; IR (neat, cm^{-1}): 2924 (br), 1653, 1458, 1397, 1347, 1158, 1094, 1009, 814, 664; HRMS (ES) calc'd for $C_{23}H_{29}N_2O_3S$ $[M+H]^+$: 413.1899, found 413.1901.

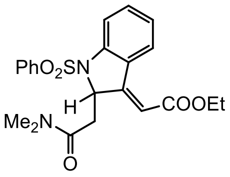

13j

13j: white foam (199 mg, 93% yield, co-eluted with ~10% of aromatized material); ^{1}H NMR (500 MHz, $CDCl_3$) δ 7.80 (dd, *J* = 8.6, 0.9 Hz, 1H), 7.70 – 7.63 (m, 2H), 7.52 – 7.45 (m, 1H), 7.46 – 7.32 (m, 4H), 7.11 (td, *J* = 7.7, 1.0 Hz, 1H), 6.05 (d, *J* = 2.3 Hz, 1H), 5.63 (ddd, *J* = 5.0, 4.9, 2.4 Hz, 1H), 4.20 (qd, *J* = 7.2, 4.3 Hz, 2H), 3.37 (dd, *J* = 15.3, 5.0 Hz, 1H), 3.26 (dd, *J* = 15.3, 4.9 Hz, 1H), 3.16 (s, 3H), 2.83 (s, 3H), 1.32 (t, J = 7.2 Hz, 3H); ^{13}C NMR (125 MHz, $CDCl_3$) δ 168.8, 133.3, 132.4, 130.2, 129.0, 127.1, 125.0, 121.6, 116.9, 107.7, 63.4, 60.2, 38.5, 37.9, 35.5, 14.3; HRMS (ES) calc'd for $C_{22}H_{25}N_2O_5S$ $[M+H]^+$: 429.1484, found 429.1491.

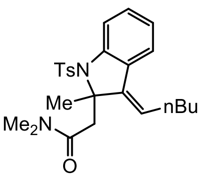

13k

13k: pale-yellow foam (196 mg, 96%); ^{1}H NMR (500 MHz, $CDCl_3$) δ 7.80 (d, *J* = 8.4 Hz, 2H), 7.55 (d, *J* = 8.2 Hz, 2H), 7.21 (d, *J* = 8.4 Hz, 0H), 7.16 (ddd, *J* = 7.9, 7.5, 1.2 Hz, 1H), 7.00 (ddd, *J* = 7.6, 7.5, 1.0 Hz, 1H), 5.31 (t, *J* = 7.0 Hz, 1H), 3.43 (d, *J* = 15.3 Hz, 1H), 2.92 (s, 3H), 2.89 (d, *J* = 15.3 Hz, 1H), 2.76 (s, 3H), 2.43 (q, J = 7.2 Hz, 2H), 2.35 (s, 3H), 1.77 (s, 3H), 1.52 – 1.43 (m, 2H), 1.37 (h, *J* = 7.2 Hz, 2H), 0.91 (t, *J* = 7.2 Hz, 3H); ^{13}C NMR (125 MHz, $CDCl_3$) δ 169.6, 156.9, 147.8, 147.4, 144.1, 135.7, 134.5, 132.4, 129.4, 127.9, 127.6, 122.7, 119.0, 118.9, 118.6, 62.1, 42.6, 37.3, 35.5, 21.5, 14.5; IR (neat, cm^{-1}): 2928, 2870, 1661, 1558, 1349, 1159, 1103, 705, 659; HRMS (ES) calc'd for $C_{25}H_{33}N_2O_3S$ $[M+H]^+$: 441.2212, found 441.2209.

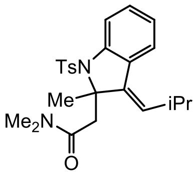

13l

13l: pale-yellow foam (167 mg, 78% yield); ^{1}H NMR (500 MHz, $CDCl_3$) δ 7.81 (d, *J* = 8.3 Hz, 2H), 7.54 (d, *J* = 7.9 Hz, 2H), 7.22 (d, *J* = 8.3 Hz, 2H), 7.16 (ddd, *J* = 8.4, 7.4, 1.0 Hz, 1H), 6.99 (ddd, *J* = 7.5, 7.4, 1.1 Hz, 1H); 5.16 (d, *J* = 9.2 Hz, 1H), 3.38 (d, *J* = 14.9 Hz, 1H), 3.11 – 3.00 (m, 1H), 2.92 (s, 3H), 2.79 (s, 3H), 2.36 (s, 3H), 1.80 (s, 3H), 1.08 (d, *J* = 6.7 Hz, 3H), 1.05 (d, *J* = 6.6 Hz, 3H); ^{13}C NMR (100 MHz, $CDCl_3$) δ 168.7, 143.5, 143.1, 138.9, 138.7, 129.8, 129.6, 128.7, 127.5, 126.8, 124.6, 122.8, 114.0, 72.9, 43.7, 38.1, 35.4, 27.9, 27.1, 22.9, 22.5, 21.5.; IR (neat, cm^{-1}): 2927 (br), 1650, 1583, 1407, 1346, 1184, 1090, 665; HRMS (ES) calc'd for $C_{24}H_{31}N_2O_3S$ $[M+H]^+$: 427.2050, found 427.2056.

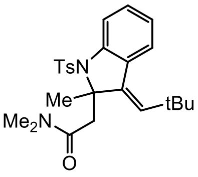

13m

13m: pale-yellow foam (75 mg, 34% yield); ^{1}H NMR (500 MHz, $CDCl_3$) δ 7.82 (d, *J* = 8.4 Hz, 2H), 7.71 (dd, *J* = 8.0, 1.3 Hz, 1H), 7.49 (d, *J* = 8.4 Hz, 1H), 7.22 (d, *J* = 8.4 Hz, 2H), 7.14 (ddd, *J* = 8.4, 7.3, 1.3 Hz, 1H), 7.00 (ddd, *J* = 8.0, 7.3, 1.1 Hz, 1H), 5.34 (s, 1H), 3.45 (d, *J* = 15.2 Hz, 1H), 2.93 (s, 3H), 2.88 (d, *J* = 15.2 Hz, 1H), 2.79 (s, 3H), 2.37 (s, 3H), 1.79 (s, 3H), 1.26 (s, 9H); ^{13}C NMR (125 MHz, $CDCl_3$) δ 168.7, 143.8, 143.5, 132.6, 129.5, 128.6, 127.5, 126.9, 122.2, 114.0, 73.9, 43.8, 37.9, 35.4, 31.5, 30.0, 21.5; IR (neat, cm^{-1}): 2930 (br), 2798, 1657, 1540, 1356, 1206, 745; HRMS (ES) calc'd for $C_{25}H_{33}N_2O_3S$ $[M+H]^+$: 441.2212, found 441.2209.

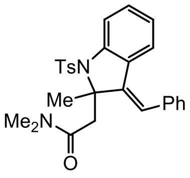

13n

13n: pale-yellow foam (218 mg, 95% yield); ^{1}H NMR (500 MHz, $CDCl_3$) δ 7.83 (d, *J* = 8.3 Hz, 1H), 7.48 (d, *J* = 8.4 Hz, 1H), 7.38 – 7.04 (m, 9H), 6.68 (ddd, *J* = 7.8, 7.3, 1.0 Hz, 1H), 6.30 (s, 1H), 3.69 (d, *J* = 15.9 Hz, 1H), 2.98 (s, 3H), 2.91 (d, *J* = 15.9 Hz, 1H), 2.77 (s, 3H), 2.37 (s, 3H), 1.87 (s, 3H); ^{13}C NMR (100 MHz, $CDCl_3$) δ 168.4, 144.0, 143.8, 143.6, 138.9, 137.2, 129.6, 128.6, 127.3, 126.8, 124.2, 122.2, 118.9, 113.9, 73.0, 43.9, 37.8, 35.4, 28.8, 21.5; IR (neat, cm^{-1}): 2928 (br), 1654, 1459, 1399, 1348, 1156, 1109, 736, 655; HRMS (ES) calc'd for $C_{27}H_{29}N_2O_3S$ $[M+H]^+$: 461.1899, found 461.1890.

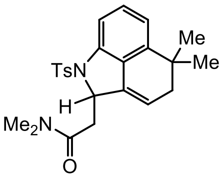

13o

13o: pale-yellow foam (203 mg, 96% yield; MW, 130 °C); ^{1}H NMR (500 MHz, $CDCl_3$) δ 7.63 (d, *J* = 8.0 Hz, 2H), 7.48 (d, *J* = 8.1 Hz, 1H), 7.21 – 7.14 (m, 3H), 6.92 (d, *J* = 7.6 Hz, 1H), 5.63 – 5.55 (m, 1H), 5.14 (d, *J* = 9.8 Hz, 1H), 3.35 (dd, *J* = 16.0, 3.2 Hz, 1H), 3.01 (s, 3H), 3.00 (s, 3H), 2.73 (dd, *J* = 16.0, 9.9 Hz, 1H), 2.34 (s, 3H), 2.24 – 2.03 (m, 2H), 1.14 (s, 3H), 1.13 (s, 3H); ^{13}C NMR (125 MHz, $CDCl_3$) δ 169.9, 144.0, 141.7, 139.9, 135.7, 129.7, 129.6, 127.4, 126.8, 118.9, 116.6, 113.3, 62.9, 41.3, 40.4, 37.2, 35.3, 33.2, 28.8, 28.1, 21.5; IR (neat, cm^{-1}): 2957 (br), 1645, 1449, 1350, 1162, 1090, 760; HRMS (ES) calc'd for $C_{24}H_{29}N_2O_3S$ $[M+H]^+$: 425.1899, found 425.1895.

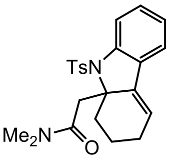

13p

13p: white foam (164 mg, 80% yield; MW, 130 °C); ^{1}H NMR (500 MHz, $CDCl_3$) δ 7.83 (ddd, *J* = 8.2, 1.0, 0.6 Hz, 1H), 7.63 (d, *J* = 8.3 Hz, 2H), 7.33 (ddd, *J* = 7.6, 1.3, 0.6 Hz, 1H), 7.20 (ddd, *J* = 8.1, 7.8, 1.4 Hz, 1H), 7.16 (d, *J* = 8.3 Hz, 2H), 7.03 (ddd, *J* = 7.5, 7.5, 1.0 Hz, 1H), 5.89 (t, *J* = 3.9 Hz, 1H), 3.10 (s, 3H), 3.01 (dt, *J* = 12.0, 3.8 Hz, 1H), 2.95 (d, *J* = 13.5 Hz, 1H), 2.82 (d, *J* = 13.5 Hz, 1H), 2.75 (s, 3H), 2.34 (s, 3H), 2.33 – 2.24 (m, 1H), 2.22 – 2.10 (m, 1H), 2.07 – 1.95 (m, 1H), 1.91 – 1.80 (m, 1H), 1.56 – 1.46 (m, 1H); ^{13}C NMR (125 MHz, $CDCl_3$) δ 169.1, 143.4, 141.6, 139.5, 139.4, 129.7, 129.6, 128.8, 126.2, 124.0, 120.4, 119.5, 114.7, 71.7, 40.9, 38.8, 35.4, 29.4, 23.2, 21.5, 18.1; IR (neat, cm^{-1}): 2930 (br), 1650, 1460, 1385, 1160, 1090, 998, 664; HRMS (ES) calc'd for $C_{23}H_{27}N_2O_3S$ $[M+H]^+$: 411.1742, found 411.1740.

Gram-Scale Application

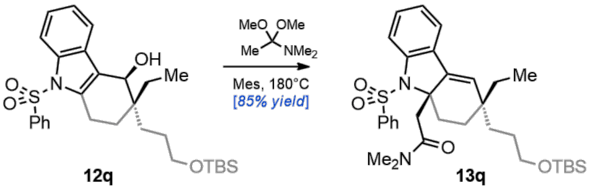

To a stirred solution of indolyl alcohol **SI-4** (1.05 g, 2 mmol) in freshly distilled mesitylene (8 mL) was added DMAA (0.88 mL, 6 mmol, 3 equiv) at room temperature. The reaction vessel was purged with argon for 20 min under vigorous stirring, then placed on oil bath pre-heated to 180 °C. After stirring for 24 h at 180 °C, the mixture was concentrated under reduced to provide the crude product, which was purified using silica gel column chromatography (EtOAc/hexanes = 1:9 to 3:7) to afford the desired [3,3]-rearrangement product **13q** (1.01 g, 85% yield) as a yellow oil.

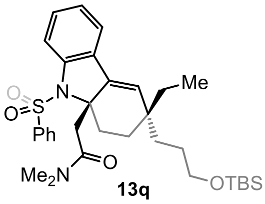

13q: ^{1}H NMR (500 MHz, $CDCl_3$) δ 7.86 (ddd, *J* = 8.2, 0.8, 0.8 Hz, 1H), 7.70 – 7.64 (m, 2H), 7.49 – 7.42 (m, 1H), 7.41 – 7.32 (m, 3H), 7.24 (ddd, *J* = 8.3, 7.5, 1.4 Hz, 1H), 7.08 (ddd, *J* = 7.5, 7.5, 1.0 Hz, 1H), 5.59 (s, 1H), 3.36 (t, *J* = 5.8 Hz, 2H), 3.09 (s, 3H), 2.91 – 2.86 (m, 1H), 2.84 (d, *J* = 13.4 Hz, 1H), 2.81 (s, 3H), 2.75 (d, *J* = 13.4 Hz, 1H), 1.96 – 1.87 (m, 1H), 1.66 – 1.55 (m, 2H), 1.41 (q, *J* = 7.4 Hz, 2H), 1.24 – 1.05 (m, 4H), 0.88 (t, *J* = 7.4 Hz, 3H), 0.85 (s, 9H), -0.01 (s, 3H), -0.02 (s, 3H); ^{13}C NMR (100 MHz, $CDCl_3$) δ 168.9, 142.6, 142.0, 139.0, 132.5, 129.8, 129.0, 128.2, 126.0, 124.4, 120.8, 115.5, 72.0, 63.6, 40.7, 38.8, 38.0, 37.0, 35.4, 33.8, 29.3, 27.7, 27.5, 26.0, 18.4, 8.6, -5.3.. HRMS (ES) calc'd for $C_{33}H_{49}N_2O_4SSi$ $[M+H]^+$: 597.3182, found 597.3175.

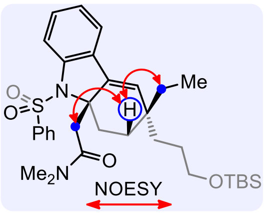

Synthesis of Enatioenriched Alcohols

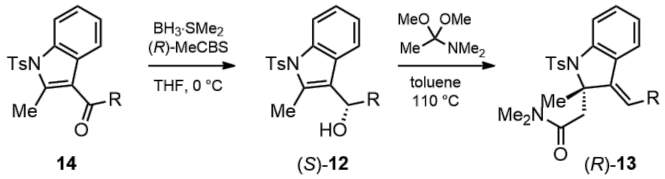

14 (S)-12 (R)-13

To a stirred solution (*R*)-MeCBS catalyst (37.5 mg, 0.13 mmol, 0.5 equiv) in THF (2.0 mL) at was added $BH_3·SMe_2$ (0.12 mL, 1.35 mmol, 5 equiv) at 0 °C. After stirring for 15 min, a solution of prochiral ketone **14** (0.27 mmol, 1 equiv) in THF (2 mL) was added dropwise over a period of 10 min. The reaction mixture was allowed to stir at 0 °C until TLC indicated complete consumption of the starting ketone (*ca.* 2 h). Then, it was quenched with MeOH (a few drops), 1 M aq. NaOH (2 mL), and diluted with EtOAc. The layers were separated, and the organic layer was washed with 1 M aq. NaOH 3-4 times (until washings were colorless), and then with brine. The organic layer was then dried over Na_2SO_4, filtered, and evaporated providing the crude product, which was purified using silica gel column chromatography (EtOAc/hexanes = 1:9 to 2:8) to afford enantioenriched 3-indolyl alcohol (*S*)-**12**.

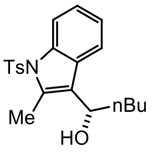

(*S*)-**12k**

(*S*)-**12k**: 98% yield;
ee = 86%, R_t (major) = 24.04 min , R_t (minor) = 20.66 min;
CHIRALPAK AS-H; 10% iPrOH/hexanes; 1 mL min^{-1}, λ = 254 nm

Signal 1: DAD1 A, Sig=254,4 Ref=360,100

Peak #	RetTime [min]	Type	Width [min]	Area [mAU*s]	Height [mAU]	Area %
1	19.396	BB	0.5737	1.16518e4	313.40497	50.0815
2	23.310	BB	0.6567	1.16139e4	270.60266	49.9185
Totals :				2.32656e4	584.00763	

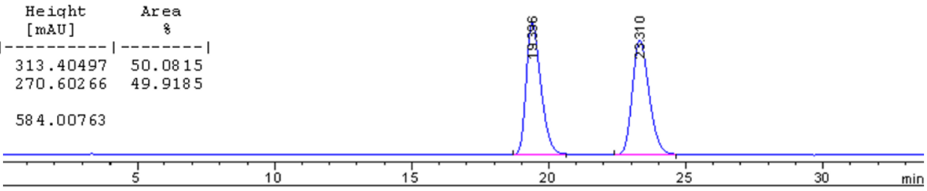

Signal 1: DAD1 A, Sig=254,4 Ref=360,100

Peak #	RetTime [min]	Type	Width [min]	Area [mAU*s]	Height [mAU]	Area %
1	20.662	VB	0.4999	2500.18140	74.81065	6.6522
2	24.045	BB	0.6226	3.50843e4	862.42682	93.3478
Totals :				3.75845e4	937.23747	

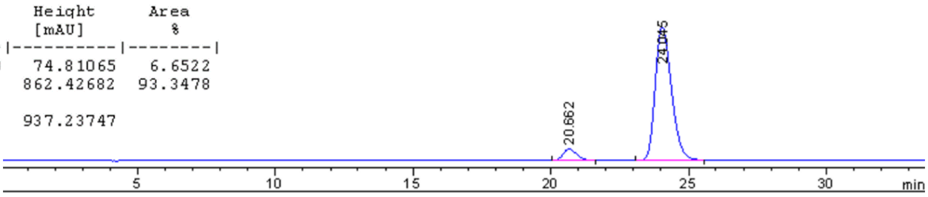

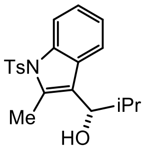

(*S*)-**12l**

(*S*)-**12l**: 98% yield;
ee = 89%, R_t (major) = 11.72 min, R_t (minor) = 12.93 min;
CHIRALPAK AS-H; 10% iPrOH/hexanes; 1 mL min^{-1}, λ = 230 nm.

Signal 1: DAD1 A, Sig=254,4 Ref=360,100

Peak #	RetTime [min]	Type	Width [min]	Area [mAU*s]	Height [mAU]	Area %
1	8.860	BB	0.4835	6138.10254	189.72243	49.9670
2	10.672	BB	0.5168	6146.21094	177.15089	50.0330
Totals :				1.22843e4	366.87332	

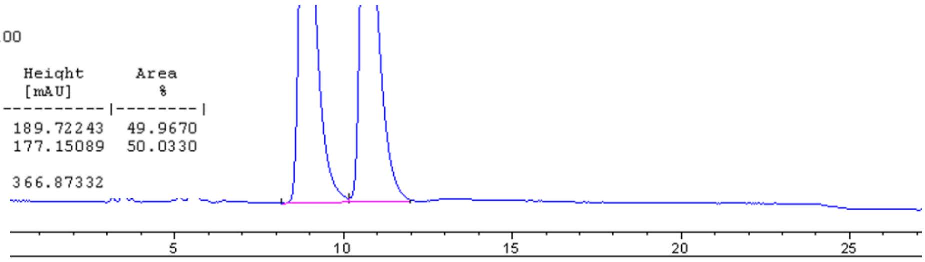

Signal 4: DAD1 D, Sig=230,16 Ref=360,100

Peak #	RetTime [min]	Type	Width [min]	Area [mAU*s]	Height [mAU]	Area %
1	11.723	MM T	0.3176	4.24271e4	2496.64453	94.5677
2	12.936	MM T	0.2725	2437.15381	149.04988	5.4323
Totals :				4.48643e4	2645.69441	

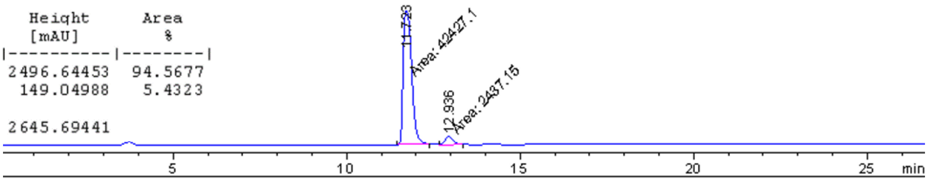

JACS JOURNAL OF THE AMERICAN CHEMICAL SOCIETY

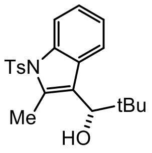

(*S*)-**12m**

(*S*)-**12m**: 97% yield;
ee = 86%, R_t (major) = 19.69 min, R_t (minor) = 23.14 min;
CHIRALPAK AS-H; 10% iPrOH/hexanes; 1 mL min^{-1}, λ = 280 nm.

Signal 1: DAD1 A, Sig=254,4 Ref=360,100

Peak #	RetTime [min]	Type	Width [min]	Area [mAU*s]	Height [mAU]	Area %
1	20.069	VB	0.7395	1.32285e4	256.57147	50.0157
2	24.015	BB	0.7100	1.32202e4	266.85010	49.9843
Totals :				2.64487e4	523.42157	

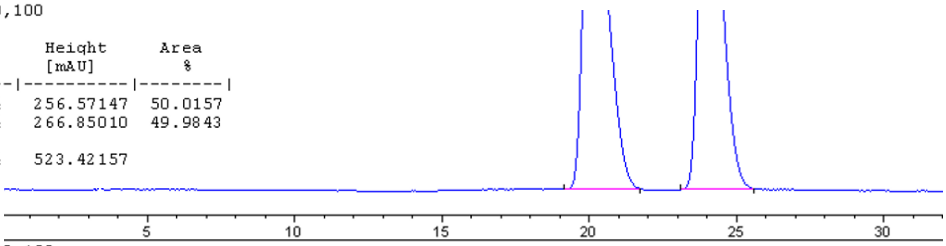

Signal 5: DAD1 E, Sig=280,16 Ref=360,100

Peak #	RetTime [min]	Type	Width [min]	Area [mAU*s]	Height [mAU]	Area %
1	19.698	BB	0.5486	8560.04395	238.56538	92.7752
2	23.149	MM T	0.6621	666.60571	16.77923	7.2248
Totals :				9226.64966	255.34461	

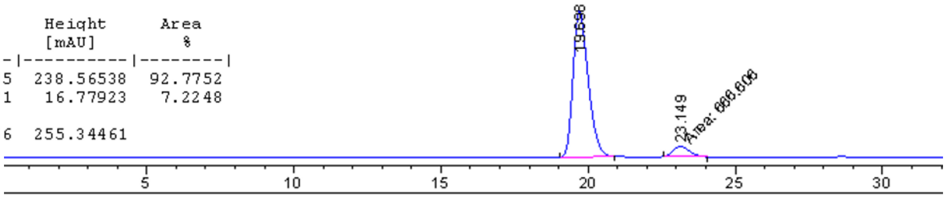

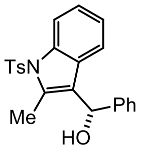

(*S*)-**12n**

(*S*)-**12n**: 95% yield;
ee = 94%, R_t (major) = 16.75 min, R_t (minor) = 20.46 min;
CHIRALPAK ID; 50% CH_2Cl_2/hexanes; 1 mL min^{-1}, λ = 254 nm.

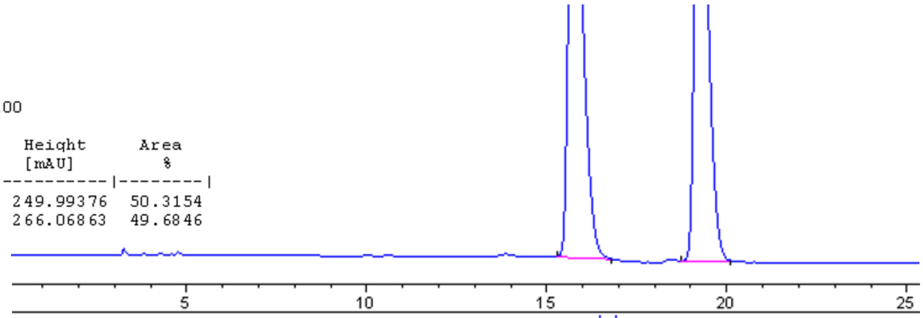

Signal 1: DAD1 A, Sig=254,4 Ref=360,100

Peak #	RetTime [min]	Type	Width [min]	Area [mAU*s]	Height [mAU]	Area %
1	15.936	BB	0.4087	6700.57471	249.99376	50.3154
2	19.463	VB	0.3861	6616.57813	266.06863	49.6846

Signal 1: DAD1 A, Sig=254,4 Ref=360,100

Peak #	RetTime [min]	Type	Width [min]	Area [mAU*s]	Height [mAU]	Area %
1	16.756	BB	0.4181	1.29584e4	481.32700	97.3628
2	20.463	BB	0.3742	350.99805	14.01513	2.6372
Totals :				1.33094e4	495.34213	

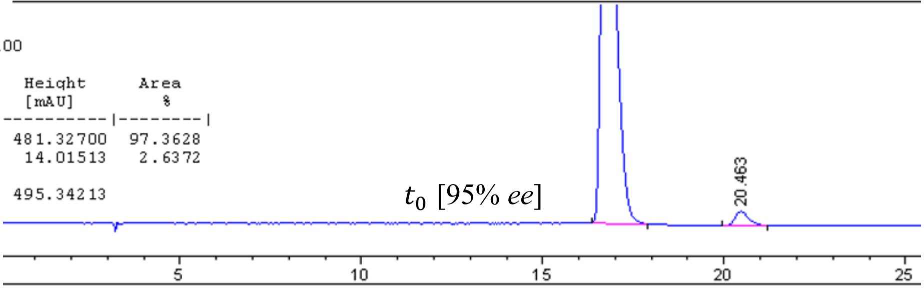

Signal 1: DAD1 A, Sig=254,4 Ref=360,100

Peak #	RetTime [min]	Type	Width [min]	Area [mAU*s]	Height [mAU]	Area %
1	16.064	MM T	0.3487	8242.42188	393.93115	94.2060
2	17.768	MM T	0.4254	506.93454	19.86246	5.7940
Totals :				8749.35641	413.79361	

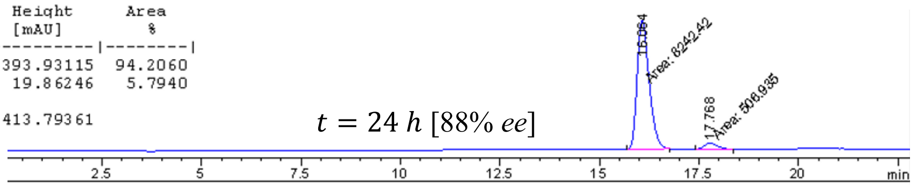

Signal 5: DAD1 E, Sig=280,16 Ref=360,100

Peak #	RetTime [min]	Type	Width [min]	Area [mAU*s]	Height [mAU]	Area %
1	15.757	BB	0.3063	1846.08069	91.92853	90.5074
2	17.386	BB	0.3411	193.62155	8.33265	9.4926
Totals :				2039.70224	100.26118	

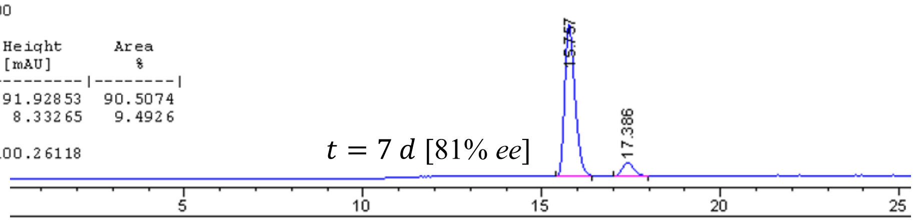

JACS
JOURNAL OF THE AMERICAN CHEMICAL SOCIETY

Chirality Transfer Experiments

Following general experimental procedure for the Indole-Clasien rearrangement, enantioenriched amides (*R*)-**13** were obtained from 3-indolyl alcohols (*S*)-**12**:

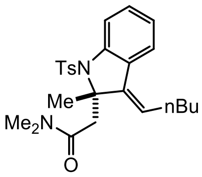

(*S*)-**13k**

(*S*)-**13k**: 95% yield;
ee = 86%, R_t (major) = 26.83 min, R_t (minor) = 23.56 min;
CHIRALPAK ID; 10-30% iPrOH/hexanes; 1 mL min^{-1}, λ = 280 nm.

Signal 5: DAD1 E, Sig=280,16 Ref=360,100

Peak #	RetTime [min]	Type	Width [min]	Area [mAU*s]	Height [mAU]	Area %
1	23.417	BB	0.4865	2.65152e4	848.79486	49.7498
2	27.117	BB	0.6240	2.67820e4	667.62836	50.2502
Totals :				5.32972e4	1516.42322	

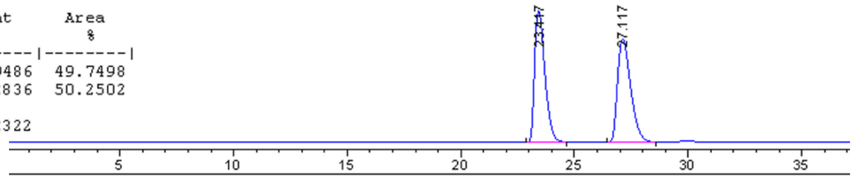

Signal 5: DAD1 E, Sig=280,16 Ref=360,100

Peak #	RetTime [min]	Type	Width [min]	Area [mAU*s]	Height [mAU]	Area %
1	23.565	MM T	0.4744	6371.39111	223.82077	6.6382
2	26.837	MM T	0.8189	8.96088e4	1823.86328	93.3618
Totals :				9.59802e4	2047.68405	

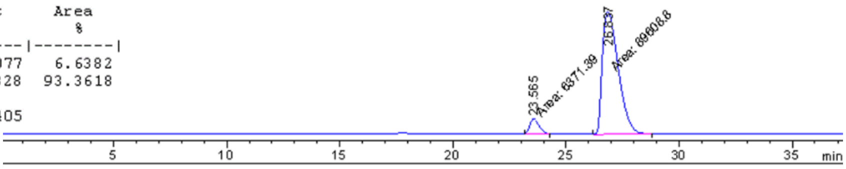

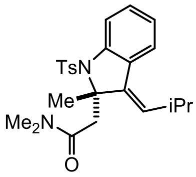

(*S*)-**13l**

(*S*)-**13l**: 78% yield;
ee = 89%, R_t (major) = 22.04 min, R_t (minor) = 25.53 min;
CHIRALPAK ID; 10-30% iPrOH/hexanes; 1 mL min^{-1}, λ = 230 nm.

Signal 1: DAD1 A, Sig=254,4 Ref=360,100

Peak #	RetTime [min]	Type	Width [min]	Area [mAU*s]	Height [mAU]	Area %
1	22.148	MM T	0.4322	2.05451e4	792.34583	51.1887
2	25.513	MM T	0.5683	1.95910e4	574.52020	48.8113
Totals :				4.01361e4	1366.86603	

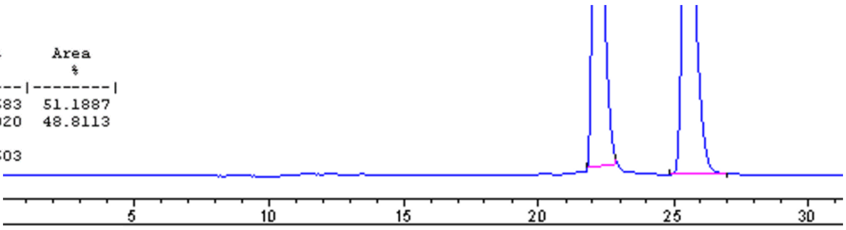

Signal 4: DAD1 D, Sig=230,16 Ref=360,100

Peak #	RetTime [min]	Type	Width [min]	Area [mAU*s]	Height [mAU]	Area %
1	22.041	MM T	0.4261	3.61080e4	1412.20581	94.5784
2	25.538	MM T	0.5030	2069.86670	68.58411	5.4216
Totals :				3.81779e4	1480.78992	

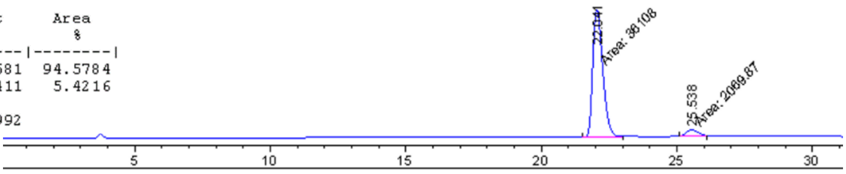

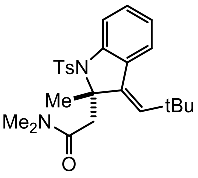

(*S*)-**13m**

(*S*)-**13m**: 38% yield;
ee = 86%, R_t (major) = 23.40 min, R_t (minor) = 28.13 min;
CHIRALPAK ID; 10-30% iPrOH/hexanes; 1 mL min^{-1}, λ = 280 nm.

Signal 5: DAD1 E, Sig=280,16 Ref=360,100

Peak #	RetTime [min]	Type	Width [min]	Area [mAU*s]	Height [mAU]	Area %
1	23.173	BB	0.4554	2.17505e4	734.65973	49.9995
2	27.796	VB	0.6250	2.17509e4	536.49957	50.0005

Totals : 4.35014e4 1271.15930

Signal 5: DAD1 E, Sig=280,16 Ref=360,100

Peak #	RetTime [min]	Type	Width [min]	Area [mAU*s]	Height [mAU]	Area %
1	23.409	BB	0.4348	3500.66528	124.96166	92.7632
2	28.130	BB	0.4584	273.10175	7.16978	7.2368

Totals : 3773.76703 132.13144

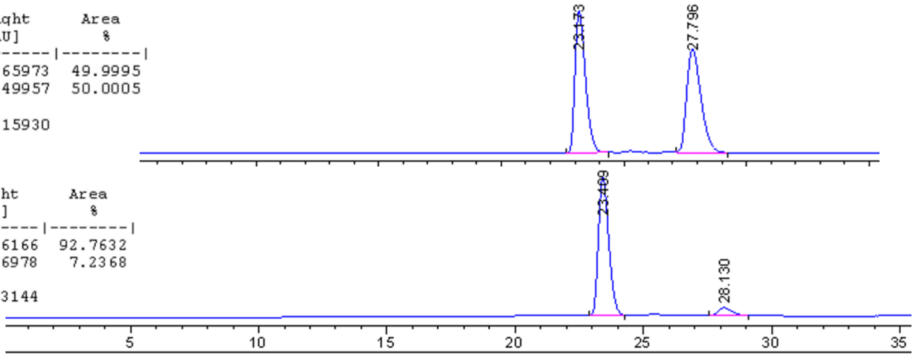

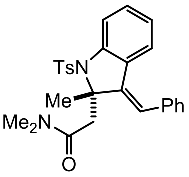

(*S*)-**13n**

(*S*)-**13m**: 78% yield;
ee = 81%, R_t (major) = 26.44 min, R_t (minor) = 28.87 min;
CHIRALPAK ID; 10-30% iPrOH/hexanes; 1 mL min^{-1}, λ = 280 nm.

Signal 5: DAD1 E, Sig=280,16 Ref=360,100

Peak #	RetTime [min]	Type	Width [min]	Area [mAU*s]	Height [mAU]	Area %
1	26.260	BB	0.5539	1.67730e4	468.25931	49.2236
2	28.492	BB	0.6439	1.73021e4	408.67520	50.7764

Totals : 3.40751e4 876.93451

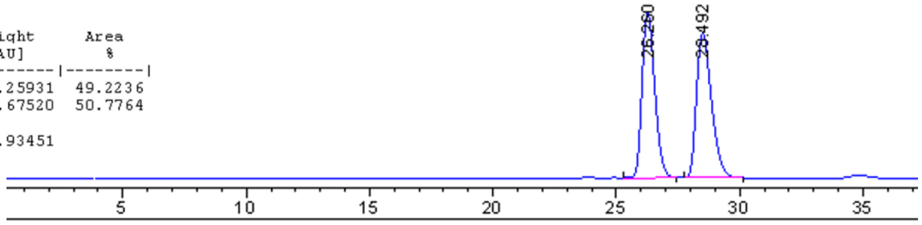

Signal 5: DAD1 E, Sig=280,16 Ref=360,100

Peak #	RetTime [min]	Type	Width [min]	Area [mAU*s]	Height [mAU]	Area %
1	26.441	MM T	0.5896	3300.77759	93.29774	90.5749
2	28.871	MM T	0.8782	343.47339	6.51834	9.4251

Totals : 3644.25098 99.81608

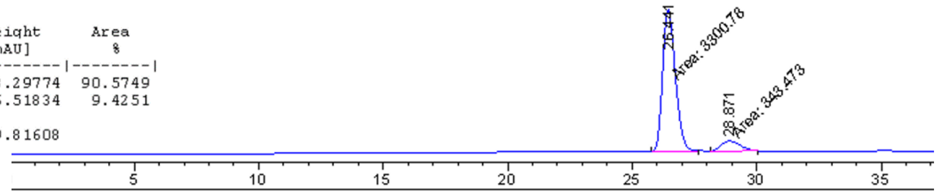

Section 2: Hinckdentine A

Synthesis of Starting Material[1]

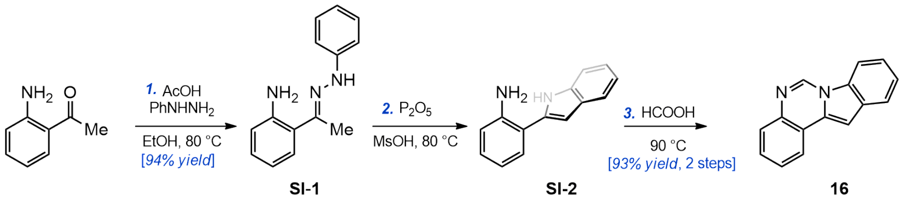

[1] Hydrazone Synthesis

To a stirred mixture of 2-aminacetophenone (10 g, 74 mmol) and phenylhydrazine (8.2 mL, 80 mmol, 1.08 equiv) in EtOH (20 mL) was added AcOH (1.2 mL, 21 mmol, 0.28 equiv) at room temperature. After stirring at 80 °C overnight, the reaction mixture was cooled to room temperature triggering crystallization of the desired hydrazone **SI-1**, which was filtered and washed with chilled EtOH (3 crops; 15.6 g, 94% yield).

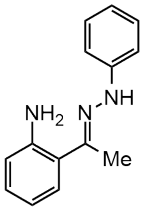

SI-1

SI-1: R_f 0.32 (EtOAc/hexanes = 1:2); visualized with UV and PMA stain; mp 104-105 °C (lit.[2] 103-108 °C); ^{1}H NMR (500 MHz, DMSO) δ 9.11 (s, 1H), 7.34 (dd, *J* = 8.0, 1.5 Hz, 1H), 7.27 – 7.19 (m, 2H), 7.09 (dt, *J* = 7.8, 1.1 Hz, 2H), 7.00 (ddd, *J* = 8.4, 7.1, 1.5 Hz, 1H), 6.76 (tt, *J* = 7.3, 1.1 Hz, 1H), 6.72 (dd, *J* = 8.1, 1.3 Hz, 1H), 6.64 (bs, 2H), 6.57 (ddd, *J* = 8.2, 7.0, 1.2 Hz, 1H); ^{13}C NMR (125 MHz, DMSO) δ 146.5, 146.1, 145.8, 129.0, 128.1, 128.1, 119.9, 118.8, 115.8, 115.2, 112.3, 14.5; HRMS (ESI) *m/z* calc'd for $C_{14}H_{15}N_3$: 225.1266, found 225.1266. No spectroscopic data was provided for this compound in the original report.

[2] Fischer Indole Synthesis

Methanesulfonic acid (100 mL) was heated at 80 °C and P_2O_5 (14.4 g, 100 mmol, 2.2 equiv) was added. The mixture was stirred at 80 °C (occasionally mixing stuck P_2O_5 with a glass rod) until all P_2O_5 dissolved (*ca.* 2 h). To this solution was added solid **SI-1** (10.0 g, 46.0 mmol) in small spatula-tip amounts over 10 min. When the addition was complete, the reaction mixture was allowed to stir for 30 min at 80 °C. Then, it was cooled to room temperature and poured over crushed ice (~ 200 mL). NaOH pellets (65 g) were then carefully added to neutralize the acid.[3] The precipitated beige-colored material was filtered, washed with excess H_2O, washed with hexanes (50 mL), and used directly in the next step without further purification.

[3] Indoloquinazoline Synthesis

A stirred solution of crude **SI-2** in formic acid (70 mL) was heated at 90 °C for 1 h. Then, it was cooled to room temperature and poured over crushed ice. The precipitate was filtered, washed with excess water, then with chilled EtOAc (50 mL), and finally with pentane (20 mL) to provide the title indoloquinazoline **16** (8.9 g, 93% yield, 2 steps) as a cream-colored solid.

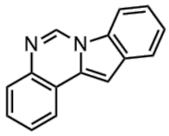

16

16: R_f 0.56 (acetone/hexanes = 1:2); visualized with UV and PMA stain; mp (EtOAc) 196-197 °C [lit.[3] (EtOH) 201 °C]; ^{1}H NMR (500 MHz, $CDCl_3$) δ 9.00 (d, *J* = 2.0 Hz, 1H), 8.04 (ddd, *J* = 7.6, 2.1, 1.7 Hz, 1H), 7.94 (d, *J* = 7.9 Hz, 1H), 7.85 – 7.77 (m, 2H), 7.54 (tdd, *J* = 7.9, 2.7, 1.6 Hz, 1H), 7.50 (tdd, *J* = 7.4, 2.7, 1.4 Hz, 1H), 7.44 (ddd, *J* = 7.1, 2.7, 1.2 Hz, 1H), 7.41 (ddd, *J* = 7.1, 2.7, 1.3 Hz, 1H), 7.11 (dt, *J* = 2.7, 0.9 Hz, 1H); ^{13}C NMR (125 MHz, $CDCl_3$) δ 139.6, 137.2, 133.0, 130.5, 129.8, 129.2, 128.3, 127.8, 124.2, 123.3, 122.4, 121.4, 121.1, 110.0, 94.8; HRMS (ESI) *m/z* calc'd for $C_{15}H_{10}N_2$: 218.0844, found 218.0844. The spectroscopic data matched that reported for this compound.[1]

---

[1] A slight modification of a previously reported procedure was used: Billimoria, A. D.; Cava, M. P. Chemistry of indolo[1,2-*c*]quinazoline: an approach to the marine alkaloid hinckdentine A. *J. Org. Chem.* **1994**, *59*, 6777–6782.

[2] Kiang, A. K.; Mann, F. G.; Prior, A. F.; Topham, A. J. *J. Chem. Soc.* **1956**, 1319–1331.

[3] A brown-colored impurity forms if the addition of NaOH is too rapid; a couple of pellets at a time is an optimal rate of addition.

Total Synthesis of (+)-Hinckdentine A

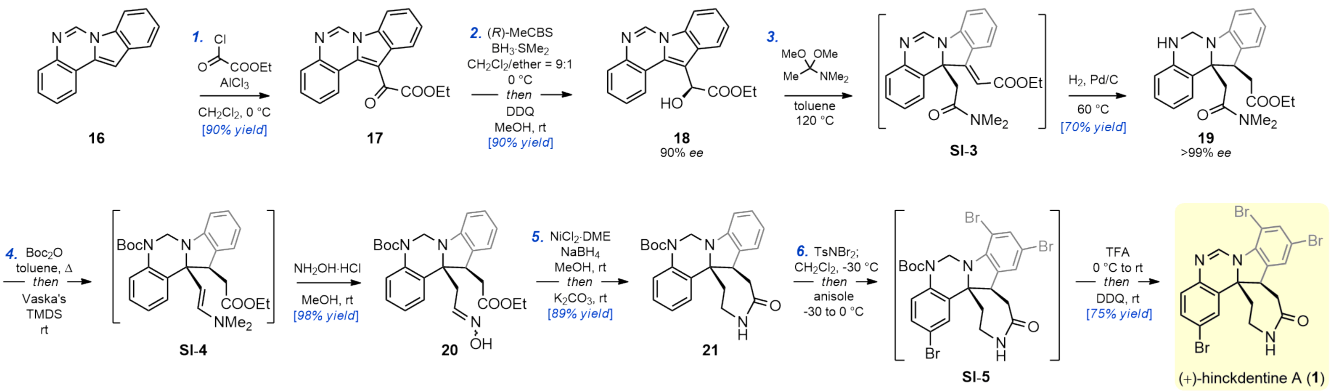

[1] Friedel-Crafts

To a stirred suspension of freshly sublimed $AlCl_3$ (3.67 g, 27.5 mmol) in CH_2Cl_2 (50 mL) was dropwise added ethyl chlorooxoacetate (3.1 mL, 27.5 mmol) at 0 °C. Cold bath was removed, and the mixture was stirred until fully homogenized (*ca.* 10 min). Then, the reaction vessel was once again cooled to 0 °C, and indoloquinazoline **16** (2.0 g, 9.2 mmol) was added as a solid in small portions. After stirring for 2 h, the mixture was quenched with sat. aq. Roschelle's salt, and stirred until clear biphasic mixture was obtained. The layers were separated, and the aq. phase was extracted with CH_2Cl_2 once. The combined organic extract was washed with sat. aq. $NaHCO_3$, dried over Na_2SO_4, filtered, and evaporated providing a yellow solid, which was washed with cold MeOH and dried to provide keto-ester **17** (2.6 g, 90% yield) as a yellow solid.

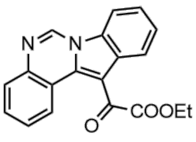
17

17: R_f 0.53 (acetone/hexanes = 1:2), visualized with UV and PMA stain; mp (MeOH) 155-159 °C, decomp.; ^{1}H NMR (500 MHz, $CDCl_3$) δ 9.36 (dd, J = 8.4, 1.4 Hz, 1H), 8.09 – 8.00 (m, 1H), 7.98 (dd, J = 8.1, 1.3 Hz, 1H), 7.93 – 7.86 (m, 1H), 7.80 (ddd, J = 8.2, 7.1, 1.4 Hz, 1H), 7.67 (ddd, J = 8.4, 7.1, 1.4 Hz, 1H), 7.53 (tt, J = 7.2, 5.6 Hz, 2H), 4.50 (q, J = 7.2 Hz, 2H), 1.40 (t, J = 7.2 Hz, 3H).; ^{13}C NMR (125 MHz, $CDCl_3$) δ 182.2, 165.9, 142.4, 138.8, 136.0, 132.4, 130.3, 128.6, 128.4, 128.2, 128.1, 126.3, 124.3, 120.4, 120.3, 110.3, 107.3, 62.6, 14.1; HRMS (ESI) *m/z* calc'd for $C_{19}H_{14}N_2O_3$: 318.1004, found 318.1004.

[2] CBS reduction

To a stirred suspension of (*R*)-MeCBS (9 mg, 0.031 mmol) in ether (5.7 mL) was dropwise added $BH_3{\cdot}SMe_2$ (2.0 M in THF, 0.16 mL, 0.31 mmol) at 0 °C. After stirring for 5 min, a solution of prochiral keto-ester **17** (20 mg, 0.063 mmol) in CH_2Cl_2 (0.6 mL) was slowly added over 10 min using a syringe pump. After addition was complete, the reaction mixture was allowed to stir for 20 min, then MeOH (2 mL) was added, and stirring was continued for another 5 min. Then, a cold bath was removed, and a solution of DDQ (16 mg, 0.07 mmol) in CH_2Cl_2 (1.2 mL) was added dropwise over 10 min at room temperature. Then, the mixture was diluted with EtOAc, and washed with sat. aq. $NaHCO_3$ three times, and once with brine. The organic layer was dried over Na_2SO_4, filtered, and evaporated providing crude material, which was purified using silica gel column chromatography (EtOAc/hexanes = 1:2 to 2:1) furnishing the desired allylic alcohol **18** (18 mg, 90% yield) as a white solid.

90% *ee*, R_t (major) = 9.8 min, R_t (minor) = 11.9 min; CHIRALPACK ID, 100% iPrOH, 1 mL min^{-1}, λ = 280 nm:

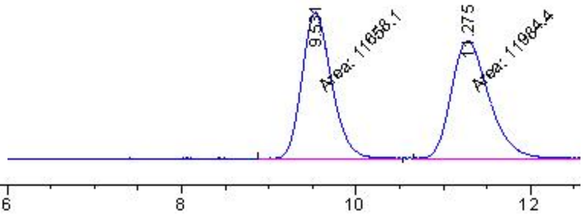
Signal 5: DAD1 E, Sig=280,16 Ref=360,100

Peak #	RetTime [min]	Type	Width [min]	Area [mAU*s]	Height [mAU]	Area %
1	9.531	MM	0.3932	1.16581e4	494.16150	49.3098
2	11.275	MM	0.5013	1.19844e4	398.46481	50.6902
Totals :				2.36425e4	892.62631	

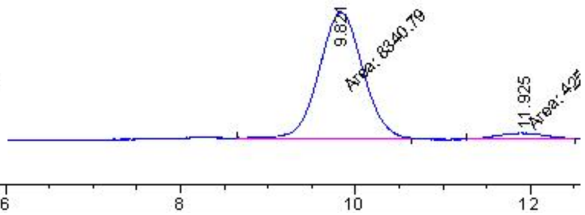
Signal 5: DAD1 E, Sig=280,16 Ref=360,100

Peak #	RetTime [min]	Type	Width [min]	Area [mAU*s]	Height [mAU]	Area %
1	9.821	MM	0.5762	8340.79492	241.26665	95.1509
2	11.925	MM	0.6197	425.06299	11.43281	4.8491
Totals :				8765.85791	252.69946	

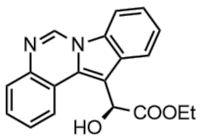

18

18: R$_f$ 0.33 (acetone/hexanes = 1:2), visualized with UV and PMA stain; $[\alpha]_D^{23}$ +142° (*c* 1.0, CHCl$_3$); mp (MeOH) 193 °C, decomp.; ^{1}H NMR (500 MHz, CDCl$_3$) δ 9.01 (s, 1H), 8.37 (dd, *J* = 7.9, 1.7 Hz, 1H), 7.96 – 7.90 (m, 1H), 7.90 – 7.84 (m, 1H), 7.83 (dd, *J* = 7.8, 1.6 Hz, 1H), 7.58 (td, *J* = 7.5, 1.6 Hz, 1H), 7.54 (td, *J* = 7.5, 1.6 Hz, 1H), 7.47 – 7.41 (m, 2H), 6.18 (s, 1H), 4.30 (dq, *J* = 10.8, 7.2 Hz, 1H), 4.18 (dq, *J* = 10.8, 7.2 Hz, 1H), 3.89 – 2.77 (bs, 1H), 1.12 (t, *J* = 7.2 Hz, 3H); ^{13}C NMR (125 MHz, CDCl$_3$) δ 173.8, 140.6, 137.1, 130.3, 129.7, 129.6, 128.5, 128.3, 127.9, 125.0, 124.5, 123.1, 121.2, 119.9, 109.9, 107.3, 66.6, 62.6, 14.2; HRMS (ESI) *m/z* calc'd for $C_{19}H_{16}N_2O_3$: 320.1161, found 320.1161.

[3] Indole-Claisen

To a stirred suspension of indolyl alcohol **18** (85 mg, 0.26 mmol) in toluene[4] (8.7 mL) was added DMAA (0.12 mL, 0.8 mmol)[5] at room temperature. The reaction mixture was placed in an oil bath preheated to 120 °C and stirred overnight. Then, it was cooled to room temperature, and 10% Pd/C (55 mg, 0.05 mmol) was added. The reaction vessel was sealed with a rubber septum, and purged with H_2. After stirring at 60 °C under balloon pressure of H_2 for 15 h, the mixture was filtered through a short Celite® pad. The filtrate was evaporated to provide a crude material, which was purified using silica gel column chromatography (acetone/hexanes = 1:4) to afford aminal **19** as a white solid.[6] This material was taken in minimal amount of acetone/hexanes = 1:9, and allowed to stand for a couple of hours. The racemate crystallized out; evaporation of the mother liquor provided enantiomerically pure material (74 mg, 70% yield).

>99% *ee*, R_t (major) = 8.2 min, R_t (major) = 9.8 min; CHIRALPACK ID, iPrOH/hexane = 1:1, 1 mL min^{-1}, λ = 254 nm:

Peak #	RetTime [min]	Type	Width [min]	Area [mAU*s]	Height [mAU]	Area %
1	8.227	MM	0.4245	8435.15137	331.16751	49.9911
2	9.827	MM	0.4847	8438.16211	290.15363	50.0089
Totals :				1.68733e4	621.32114	

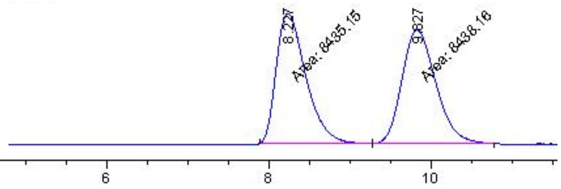

Signal 2: DAD1 B, Sig=254,16 Ref=360,100

Peak #	RetTime [min]	Type	Width [min]	Area [mAU*s]	Height [mAU]	Area %
1	7.955	MM	0.5302	6023.82715	189.37105	100.0000
Totals :				6023.82715	189.37105	

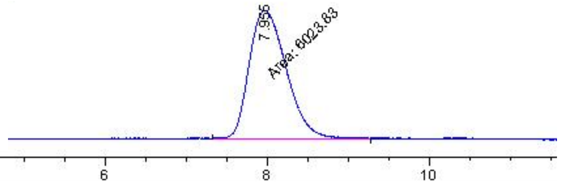

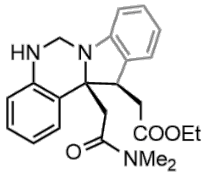

19

19: R$_f$ 0.19 (acetone/hexanes = 1:2), visualized with UV and PMA stain; $[\alpha]_D^{23}$ +82° (*c* 1.0, CHCl$_3$); mp (acetone) 177-178 °C; ^{1}H NMR (500 MHz, CDCl$_3$) δ 7.35 – 7.29 (m, 1H), 7.09 (ddt, *J* = 7.3, 1.2, 0.6 Hz, 1H), 7.06 (td, *J* = 7.6, 1.3 Hz, 1H), 6.94 – 6.87 (m, 1H), 6.71 (d, *J* = 8.1 Hz, 1H), 6.68 (td, *J* = 7.4, 1.0 Hz, 1H), 6.63 (ddd, *J* = 7.9, 7.2, 1.3 Hz, 1H), 6.39 – 6.33 (m, 1H), 4.84 (dd, *J* = 13.3, 3.8 Hz, 1H), 4.72 (dd, *J* = 13.3, 3.0 Hz, 1H), 4.23 – 4.08 (m, 4H), 3.76 (dd, *J* = 14.8, 3.9 Hz, 1H), 3.28 (d, *J* = 14.1 Hz, 1H), 2.84 (s, 3H), 2.84 (d, *J* = 14.1 Hz, 1H), 2.50 (s, 3H), 2.48 (dd, *J* = 14.8, 11.1 Hz, 1H), 1.24 (t, J = 7.2 Hz, 2H); ^{13}C NMR (125 MHz, CDCl$_3$) δ 172.6, 169.9, 148.2, 141.9, 133.1, 128.1, 127.8, 127.6, 125.6, 124.9, 120.3, 118.0, 115.8, 109.6, 69.9, 60.3, 52.0, 46.8, 39.0, 37.6, 37.5, 35.6, 14.3; HRMS (ESI) *m/z* calc'd for $C_{23}H_{27}N_3O_3$: 393.2052, found 393.2077.

[4] Amide reduction

To a stirred suspension of aminal **19** (40 mg, 0.1 mmol) in toluene (2.5 mL) was added Boc$_2$O (44 mg, 0.2 mmol) at room temperature. After refluxing the reaction mixture overnight (*ca.* 16 h), it was cooled to room temperature, and Vaska's complex (0.8 mg, 1 μmol), followed by TMDS (53 μL, 0.3 mmol) were added. After stirring for 5 min, the mixture was diluted with MeOH (1.5 mL), and $NH_2OH \cdot HCl$ (10 mg, 0.15 mmol) was added. The reaction mixture was allowed to stir for 20 min, whereupon a clean formation of the desired oxime was observed. The mixture was evaporated to dryness, providing a crude material, which was purified using silica gel column chromatography (acetone/hexanes = 1:9 → 1:4 → 1:2) furnishing oxime **20** (45 mg, 98% yield, *cis*/*trans* = 1:1) as a white solid.

[4] Toluene was degassed by freeze-pump-thaw (three cycles) shortly before use

[5] DMAA was freshly distilled from CaH_2

[6] While the hydrogenation event proceeds highly stereoselectively, resulting in a single diastereomer of **19**, the overall yield of this one-pot transformation (**18**→**SI-3**) is dictated by the [3,3] rearrangement step. The details on this transformation will be published in the upcoming full paper.

JACS
JOURNAL OF THE AMERICAN CHEMICAL SOCIETY

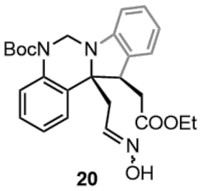

20

20: R_f 0.34 (acetone/hexanes = 1:2), visualized with UV and PMA stain; $[\alpha]_D^{23}$ +58° (*c* 0.5, $CHCl_3$); ^1H NMR (500 MHz, $CDCl_3$) *mixture* δ 8.85 (s, 0.5H), 8.28 (s, 0.5H), 7.63 (s, 1H), 7.51 (dt, *J* = 7.7, 2.4 Hz, 1H), 7.45 – 7.30 (m, 1H), 7.19 – 7.05 (m, 3H), 7.04 – 6.95 (m, 2H), 6.90 (dd, J = 13.1, 8.0 Hz, 1H), 6.83 – 6.72 (m, 1H), 6.08 – 5.83 (m, 1H), 4.48 (dd, J = 14.0, 9.5 Hz, 1H), 4.27 – 4.20 (m, 2H), 4.20 – 4.14 (m, 1H), 3.45 (dd, *J* = 18.0, 6.3 Hz, 0.5H), 2.94 (d, *J* = 0.0 Hz, 2H), 2.83 (dd, *J* = 17.9, 3.5 Hz, 0.5H), 2.63 – 2.51 (m, 1H), 1.49 (s, 9H), 1.33 – 1.25 (m, 3H); ^{13}C NMR (126 MHz, $CDCl_3$) *mixture* δ 172.7, 172.6, 153.3, 149.4, 148.5, 148.0, 148.0, 136.8, 136.4, 133.6, 132.5, 132.3, 129.9, 129.1, 128.2, 128.1, 127.1, 127.1, 126.9, 124.8, 124.7, 124.5, 124.5, 123.5, 121.2, 120.0, 119.0, 111.9, 81.7, 81.7, 69.4, 69.0, 61.1, 55.8, 55.3, 50.0, 49.7, 38.3, 38.1, 37.9, 33.1, 28.3, 14.3, 14.3; HRMS (ESI) *m/z* calc'd for $C_{26}H_{31}N_3O_5$: 465.2264, found 465.2249.

[5] Lactamization

To a stirred solution of oxime **20** (30 mg, 0.065 mmol) in MeOH (2.6 mL) were sequentially added $NiCl_2$·DME (28 mg, 0.13 mmol) and $NaBH_4$ (25 mg, 0.65 mmol) at 0°C. After stirring for 1 h at room temperature, K_2CO_3 (175 mg, 1.3 mmol) was added, and the mixture was allowed to stir overnight. Then, the mixture was filtered through a pad of packed Celite®. The filtrate was evaporated providing crude material, which was purified using silica gel column chromatography (MeOH/CH_2Cl_2 = 1% to 3%) furnishing caprolactam **21** (24 mg, 89% yield) as a white solid.

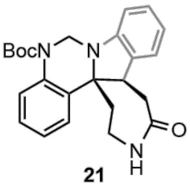

21

21: R_f 0.3 (acetone/hexanes = 1:1), visualized with UV and PMA stain; $[\alpha]_D^{23}$ +28° (*c* 0.45, $CHCl_3$); mp (acetone) 233 °C, decomp.; ^1H NMR (500 MHz, $CDCl_3$) δ 7.58 (s, 1H), 7.25 (dd, *J* = 8.0, 1.7 Hz, 1H), 7.12 – 7.05 (m, 2H), 7.05 – 7.00 (m, 2H), 6.91 (d, J = 7.9 Hz, 1H), 6.79 (td, *J* = 7.5, 1.0 Hz, 1H), 6.48 – 6.42 (m, 1H), 5.97 (d, *J* = 13.9 Hz, 1H), 4.53 (d, *J* = 13.9 Hz, 1H), 3.85 – 3.72 (m, 2H), 3.08 (dtd, *J* = 14.8, 6.3, 2.0 Hz, 1H), 2.99 (dd, *J* = 14.0, 12.2 Hz, 1H), 2.48 (ddd, *J* = 14.0, 3.5, 1.7 Hz, 1H), 2.43 (dd, *J* = 16.0, 6.1 Hz, 1H), 2.10 (ddd, *J* = 16.1, 10.9, 2.0 Hz, 1H), 1.49 (s, 9H); ^{13}C NMR (125 MHz, $CDCl_3$) δ 174.8, 147.4, 136.5, 133.3, 133.1, 127.9, 127.2, 126.9, 124.5, 123.9, 123.2, 121.8, 112.4, 81.9, 70.9, 54.0, 47.0, 40.4, 39.6, 37.3, 28.4.; HRMS (ESI) *m/z* calc'd for $C_{24}H_{27}N_3O_3$: 405.2050, found 405.2077.

[6] Bromination

To a stirred solution of caprolactam **21** (35 mg, 0.09 mmol) in CH_2Cl_2 (2.1 mL) was added $TsNBr_2$ (85 mg, 0.26 mmol)[7] at -30 °C. After stirring for 2 h, anisole (93 µL, 0.86 mmol) was added and the mixture was warmed up to 0 °C. After stirring for 15 min, TFA (1 mL) was added, and the mixture was allowed to stir at room temperature for 1 h, whereupon DDQ (23 mg, 0.1 mmol) was introduced. After stirring for 10 min, the mixture was concentrated under reduced pressure, re-dissolved in EtOAc, and washed with sat. aq. $NaHCO_3$ several times, followed by brine wash. The organic layer was dried over Na_2SO_4, filtered, and evaporated providing crude material, which was purified using silica gel column chromatography (MeOH/CH_2Cl_2 = 1% to 2%) furnishing hinckdentine A (**1**, 34 mg, 75% yield) as a white solid.

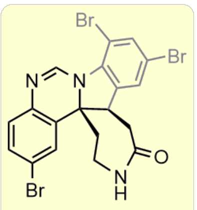

(+)-**1**

(+)-**1**: R_f 0.19 (CH_2Cl_2/MeOH = 19:1), visualized with UV and PMA stain; $[\alpha]_D^{22}$ +270° (*c* 1.0, $CHCl_3$); mp (acetone) 229-230 °C, decomp. (lit.[8] >250 °C, decomp.); ^1H NMR (500 MHz, $CDCl_3$) δ 9.08 (s, 1H), 7.59 (dd, *J* = 1.5, 1.5 Hz, 1H), 7.50 – 7.46 (m, 2H), 7.42 (d, *J* = 2.1 Hz, 1H), 7.23 (d, *J* = 8.4 Hz, 1H), 5.76 (bs, 1H), 4.11 (d, *J* = 5.7 Hz, 1H), 3.48 (dd, *J* = 16.2, 2.9 Hz, 1H), 3.35 (dd, *J* = 16.2, 6.2 Hz, 1H), 3.20 – 3.11 (m, 1H), 3.05 – 2.96 (m, 1H), 2.20 (dd, *J* = 15.2, 8.9 Hz, 1H), 1.96 (dd, *J* = 15.2, 8.5 Hz, 1H); ^{13}C NMR (125 MHz, $CDCl_3$) δ 171.9, 141.3, 139.7, 137.7, 136.6, 134.5, 132.4, 131.5, 127.6, 127.3, 125.5, 119.1, 116.7, 104.8, 66.9, 45.6, 37.1, 36.5, 35.2; HRMS (ESI) *m/z* calc'd for $C_{19}H_{14}Br_3N_3O$: 536.8687, found 536.8694. The spectroscopic data for the synthetic sample matched those reported for the natural product (see pp. 76-77).

---

[7] See next page for optimization of reaction conditions.

[8] See Appendix.

JACS JOURNAL OF THE AMERICAN CHEMICAL SOCIETY

Bromination

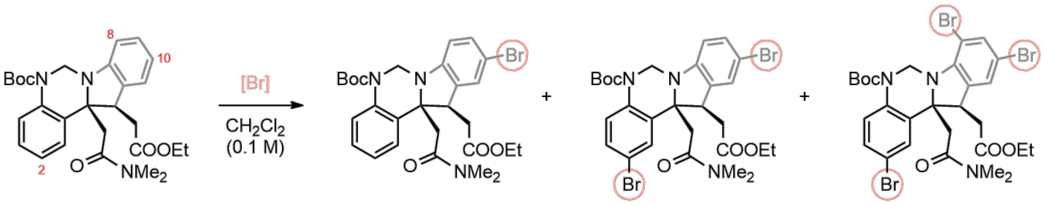

SI-6 SI-7 SI-8 SI-9

entry	[Br]	equiv	cat.	*t*, °C	time	**SI-7**, %[a]	**SI-8**, %[a]	**SI-9**, %[a]
1	NBS	3	-	rt	30 min	98	-	-
2	NBS	4	-	rt	30 min	<5	90	-
3[b]	NBS	4	-	rt	on	-	-	-
4	DBDMH	3	-	rt	30 min	-	90	-
5[b]	DBDMH	3	-	rt	on	-	-	-
6[b]	Br_2	3	-	rt	10 min	-	-	-
7	NBS	4	CAN (2.5 mol%)	rt	40 min	-	-	41
8	NBS	4	CAN (2.5 mol%)	0	2 h	-	-	55
9	NBS	4	CAN (2.5 mol%)	-15	6 h	-	-	59
10	$TsNBr_2$	2	-	rt	30 min	-	-	57
11	$TsNBr_2$	2	-	0	1 h	-	-	61
12	$TsNBr_2$	2	-	-15	2 h	-	-	76
13	$TsNBr_2$	2	-	-30	4 h	-	-	76

[a] isolated yields; [b] decomposition

Optimal bromination protocol was developed during the course of an early generation of the synthesis. Optimized reaction conditions were then applied to caprolactam **21** (see *step 5*, p. 16).

Thus, isolation of carbamate **SI-6** (see *step 4*, p. 15) provided an ideal model substrate for bromination experiments. Electrophilic aromatic substitution at C2 was found to be extremely challenging; none of the standard brominating agents (e.g. NBS, DBDMH; entries 2 and 4, respectively) could promote the desired transformation. Prolonged exposure of the substrate to electrophilic bromine source consistently resulted in a complex mixture formation, which did not contain any of the desired product (entries 3 and 5). Although, a number of Lewis base additives (not shown) provided the desired tribromide **SI-9** in poor yields, ammonium cerium (IV) nitrate (CAN) catalyzed the bromination and furnished synthetically useful yields of **SI-9** (entries 7-9, presumably *via* transient bromonium cerium (IV) nitrate). Finally, treatment of **SI-6** with a much stronger brominating agent, namely *N*,*N*-dibromo-*p*-toluenesulfonamide ($TsNBr_2$),[9] at low temperatures smoothly returned **SI-9** in good yield (entries 12 and 13). Gratifyingly, bromination of caprolactam **21** turned out to be a much easier task compared to amide **SI-6**.

[9] Preparation of $TsNBr_2$: To a vigorously stirred suspension of chloramine-T trihydrate (3 g, 13.2 mmol) in H_2O (43 mL) was dropwise added molecular Br_2 (0.67 mL, 13.2 mmol) using a pipette. After addition was complete, the mixture was sonicated, then stirred for additional 5 min. The golden yellow suspension was filtered, thoroughly washed with H_2O, washed with hexanes, and dried providing the desired brominating agent (3 g, 70% yield). A vial containing the material was wrapped with Al-foil and stored in a freezer to avoid degradation. Spectral data for $TsNBr_2$ are in agreement with that previously reported. See: Saikia, I.; Chakraborty, P.; Sarma, M. J.; Goswami, M.; Phukan, P. Rapid and total bromination of aromatic compounds using $TsNBr_2$ without any catalyst. *Synth. Commun.* **2015**, *45*, 211−217.

Section 3: NMR Spectra

^{1}H NMR Spectrum of **10** (500 MHz, $CDCl_3$, 25 °C) 20
^{13}C NMR Spectrum of **10** (125 MHz, $CDCl_3$, 25 °C) 21
^{1}H NMR Spectrum of **11** (500 MHz, $CDCl_3$, 25 °C) 22
^{13}C NMR Spectrum of **11** (125 MHz, $CDCl_3$, 25 °C) 23
^{1}H NMR Spectrum of **13a** (E/Z = 2:1; 500 MHz, $CDCl_3$, 25 °C) 24
^{13}C NMR Spectrum of **13a** (E/Z = 2:1; 125 MHz, $CDCl_3$, 25 °C) 25
^{1}H NMR Spectrum of **13b** (E/Z = 1:1; 500 MHz, $CDCl_3$, 25 °C) 26
^{13}C NMR Spectrum of **13b** (E/Z = 1:1; 125 MHz, $CDCl_3$, 25 °C) 27
^{1}H NMR Spectrum of **13c** (E/Z = 5:1; 500 MHz, $CDCl_3$, 25 °C) 28
^{13}C NMR Spectrum of **13c** (E/Z = 5:1; 125 MHz, $CDCl_3$, 25 °C) 29
^{1}H NMR Spectrum of **13d** (E/Z = 2:1; 500 MHz, $CDCl_3$, 25 °C) 30
^{13}C NMR Spectrum of **13d** (E/Z = 2:1; 125 MHz, $CDCl_3$, 25 °C) 31
^{1}H NMR Spectrum of **13e** (E/Z = 3:2; 500 MHz, $CDCl_3$, 25 °C) 32
^{13}C NMR Spectrum of **13e** (E/Z = 3:2; 125 MHz, $CDCl_3$, 25 °C) 33
^{1}H NMR Spectrum of **13f** (E/Z = 4:1; 500 MHz, $CDCl_3$, 25 °C) 34
^{13}C NMR Spectrum of **13f** (E/Z = 4:1; 125 MHz, $CDCl_3$, 25 °C) 35
^{1}H NMR Spectrum of **13g** (500 MHz, $CDCl_3$, 25 °C) 36
^{13}C NMR Spectrum of **13g** (125 MHz, $CDCl_3$, 25 °C) 37
^{1}H NMR Spectrum of **13h** (E/Z = 5:1; 500 MHz, $CDCl_3$, 25 °C) 38
^{13}C NMR Spectrum of **13h** (E/Z = 5:1; 125 MHz, $CDCl_3$, 25 °C) 39
^{1}H NMR Spectrum of **13i** (500 MHz, $CDCl_3$, 25 °C) 40
^{13}C NMR Spectrum of **13i** (125 MHz, $CDCl_3$, 25 °C) 41
^{1}H NMR Spectrum of **13j** (E only, indoline/indole = 10:1; 500 MHz, $CDCl_3$, 25 °C) 42
^{13}C NMR Spectrum of **13j** (E only, indoline/indole = 10:1; 125 MHz, $CDCl_3$, 25 °C) 43
^{1}H NMR Spectrum of **13k** (500 MHz, $CDCl_3$, 25 °C) 44
^{13}C NMR Spectrum of **13k** (125 MHz, $CDCl_3$, 25 °C) 45
^{1}H NMR Spectrum of **13l** (500 MHz, $CDCl_3$, 25 °C) 46
^{1}H NMR Spectrum of **13l** (100 MHz, $CDCl_3$, 25 °C) 47
^{1}H NMR Spectrum of **13m** (500 MHz, $CDCl_3$, 25 °C) 48
^{13}C NMR Spectrum of **13m** (125 MHz, $CDCl_3$, 25 °C) 49
^{1}H NMR Spectrum of **13n** (500 MHz, $CDCl_3$, 25 °C) 50
^{13}C NMR Spectrum of **13n** (100 MHz, $CDCl_3$, 25 °C) 51
^{1}H NMR Spectrum of **13o** (500 MHz, $CDCl_3$, 25 °C) 52
^{13}C NMR Spectrum of **13o** (125 MHz, $CDCl_3$, 25 °C) 53
^{1}H NMR Spectrum of **13p** (500 MHz, $CDCl_3$, 25 °C) 54
^{13}C NMR Spectrum of **13p** (125 MHz, $CDCl_3$, 25 °C) 55
^{1}H NMR Spectrum of **13q** (500 MHz, $CDCl_3$, 25 °C) 56
^{13}C NMR Spectrum of **13q** (100 MHz, $CDCl_3$, 25 °C) 57
^{1}H−^{1}H NOESY Spectrum of **13q** (500 MHz, $CDCl_3$, 25 °C) 58

Expanded $^{1}H-^{1}H$ NOESY Spectrum of **13q** (500 MHz, $CDCl_3$, 25 °C) 59
^{1}H NMR Spectrum of **SI-6** (500 MHz, DMSO-d_6, 25 °C) 60
^{13}C NMR Spectrum of **SI-6** (125 MHz, DMSO-d_6, 25 °C) 61
^{1}H NMR Spectrum of **16** (500 MHz, $CDCl_3$, 25 °C) 62
^{13}C NMR Spectrum of **16** (125 MHz, $CDCl_3$, 25 °C) 63
^{1}H NMR Spectrum of **17** (500 MHz, $CDCl_3$, 25 °C) 64
^{13}C NMR Spectrum of **17** (125 MHz, $CDCl_3$, 25 °C) 65
^{1}H NMR Spectrum of **18** (500 MHz, $CDCl_3$, 25 °C) 66
^{13}C NMR Spectrum of **18** (125 MHz, $CDCl_3$, 25 °C) 67
^{1}H NMR Spectrum of **19** (500 MHz, $CDCl_3$, 25 °C) 68
^{13}C NMR Spectrum of **19** (125 MHz, $CDCl_3$, 25 °C) 69
^{1}H NMR Spectrum of **20** (*cis*/*trans* = 1:1; 500 MHz, $CDCl_3$, 25 °C) 70
^{13}C NMR Spectrum of **20** (*cis*/*trans* = 1:1; 125 MHz, $CDCl_3$, 25 °C) 71
^{1}H NMR Spectrum of **21** (500 MHz, $CDCl_3$, 25 °C) 72
^{13}C NMR Spectrum of **21** (125 MHz, $CDCl_3$, 25 °C) 73
^{1}H NMR Spectrum of (+)-hinckdentine A (500 MHz, $CDCl_3$, 25 °C) 74
^{13}C NMR Spectrum of (+)-hinckdentine A (125 MHz, $CDCl_3$, 25 °C) 75
Stacked ^{1}H NMR spectra of (+)-hinckdentine A 76
Stacked ^{13}C NMR spectra of (+)-hinckdentine A 77

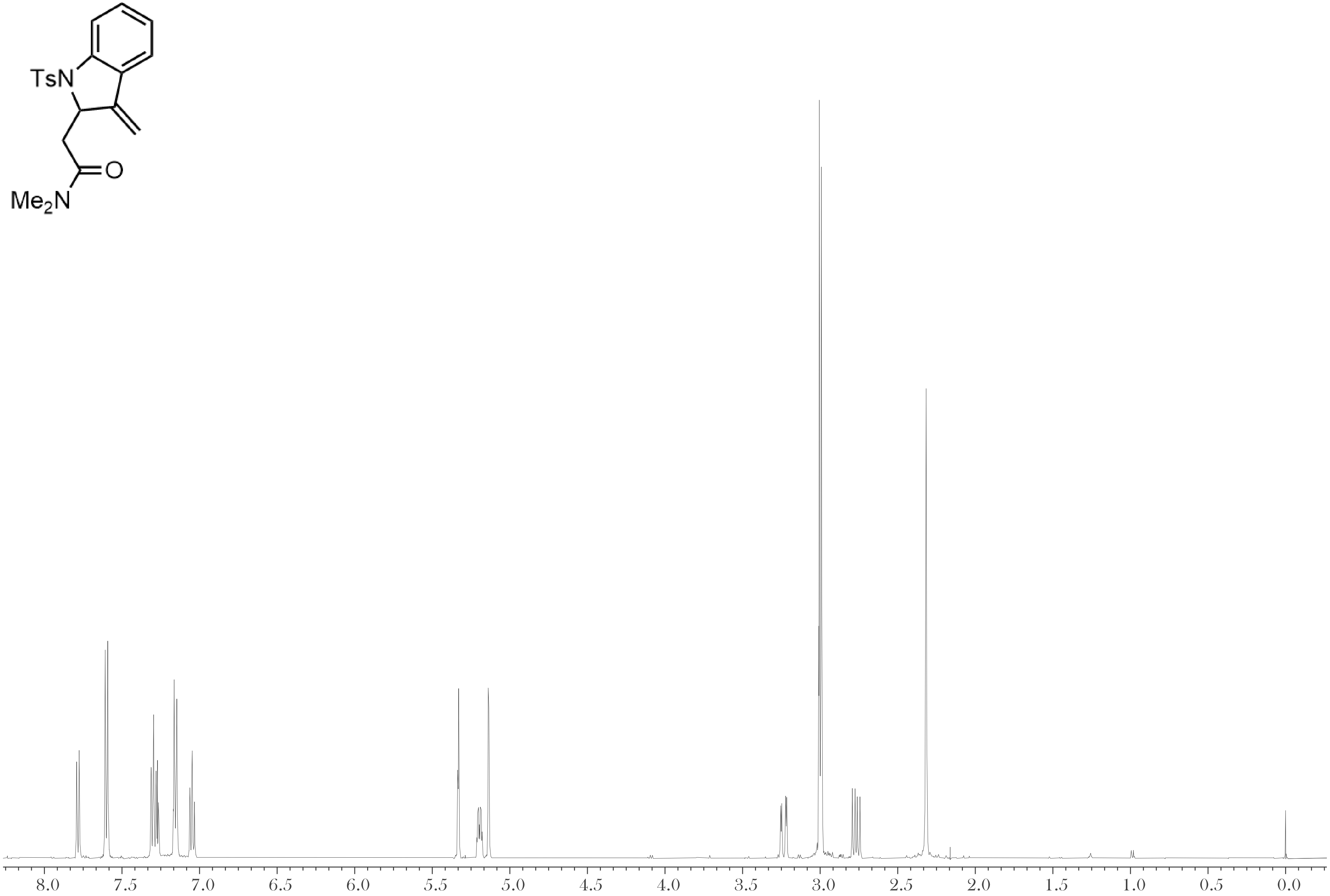

^{1}H NMR Spectrum of **10** (500 MHz, $CDCl_3$, 25 °C)

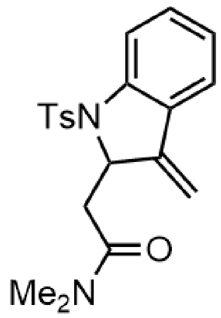

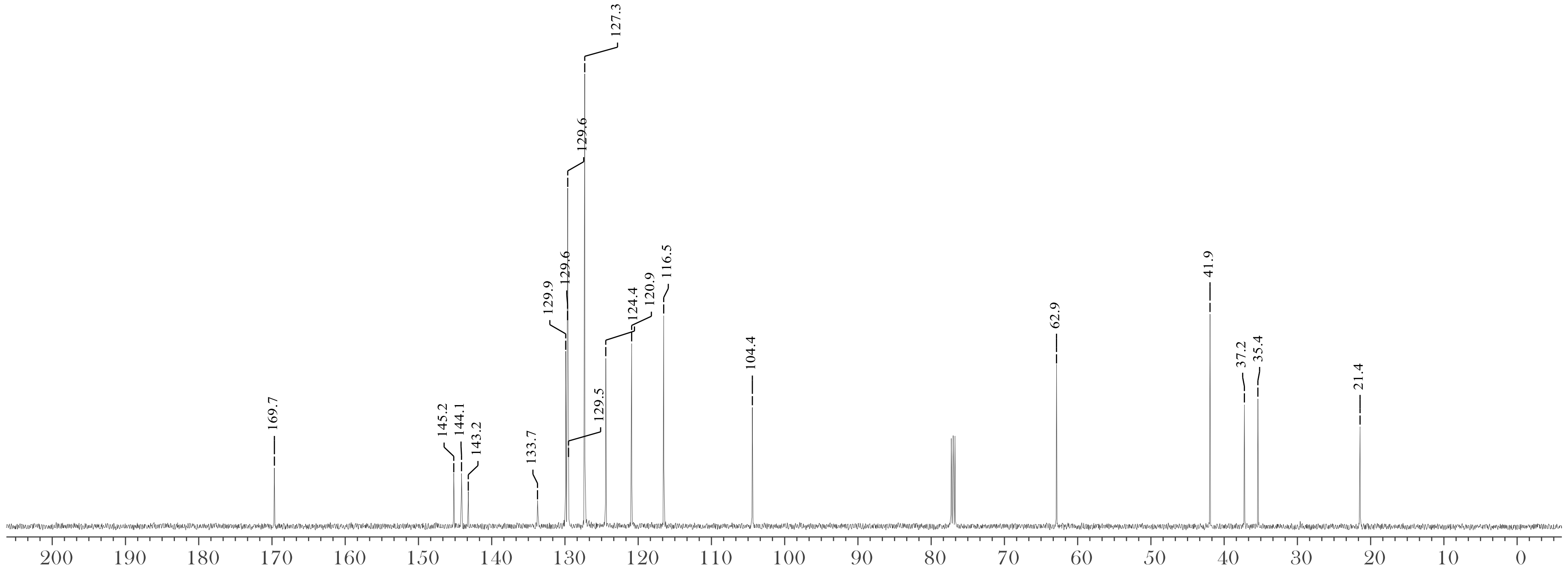

^{13}C NMR Spectrum of **10** (125 MHz, $CDCl_3$, 25 °C)

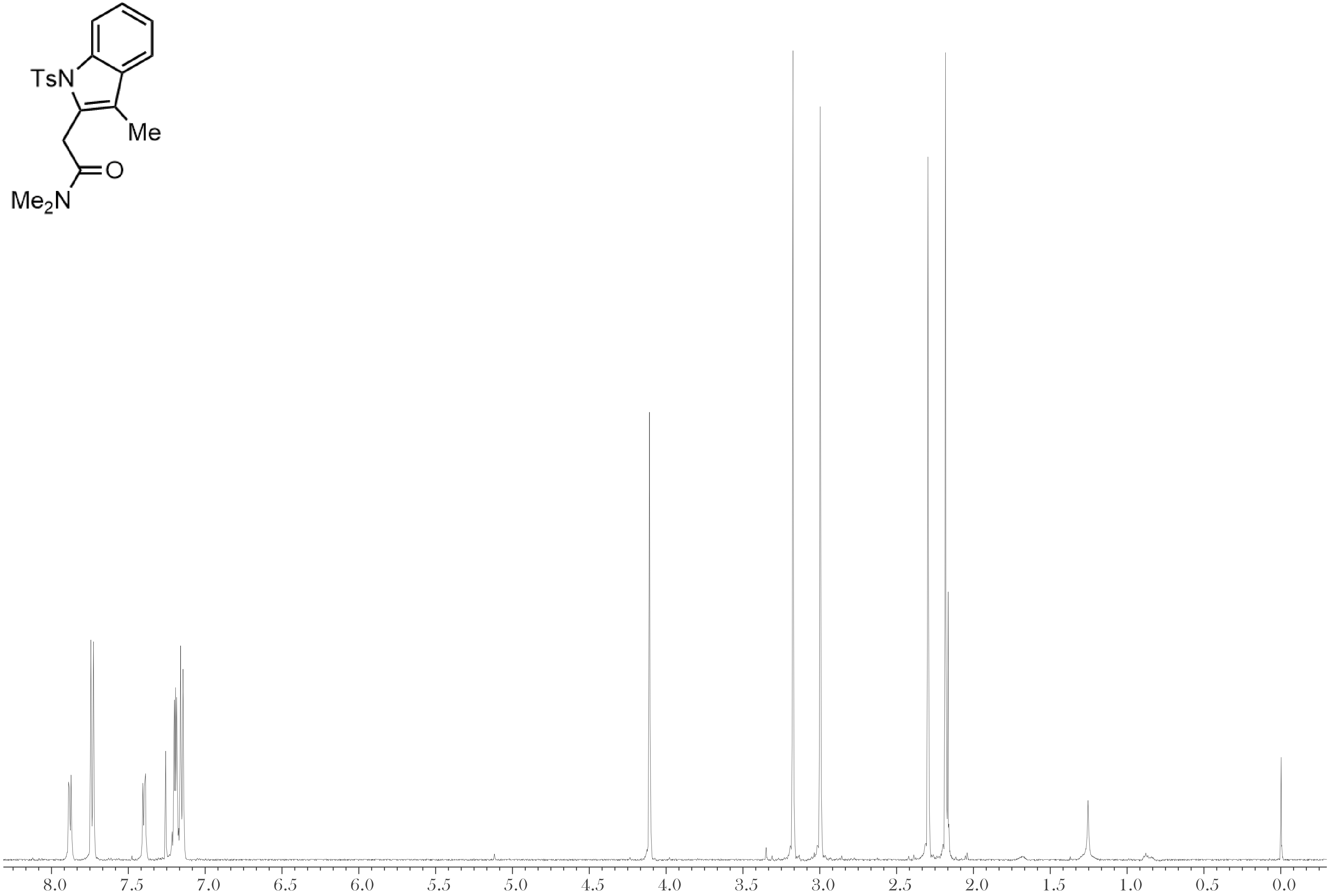

^{1}H NMR Spectrum of **11** (500 MHz, $CDCl_3$, 25 °C)

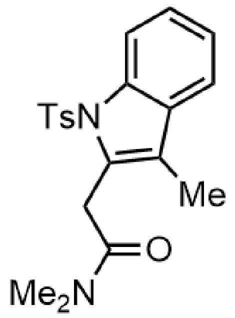

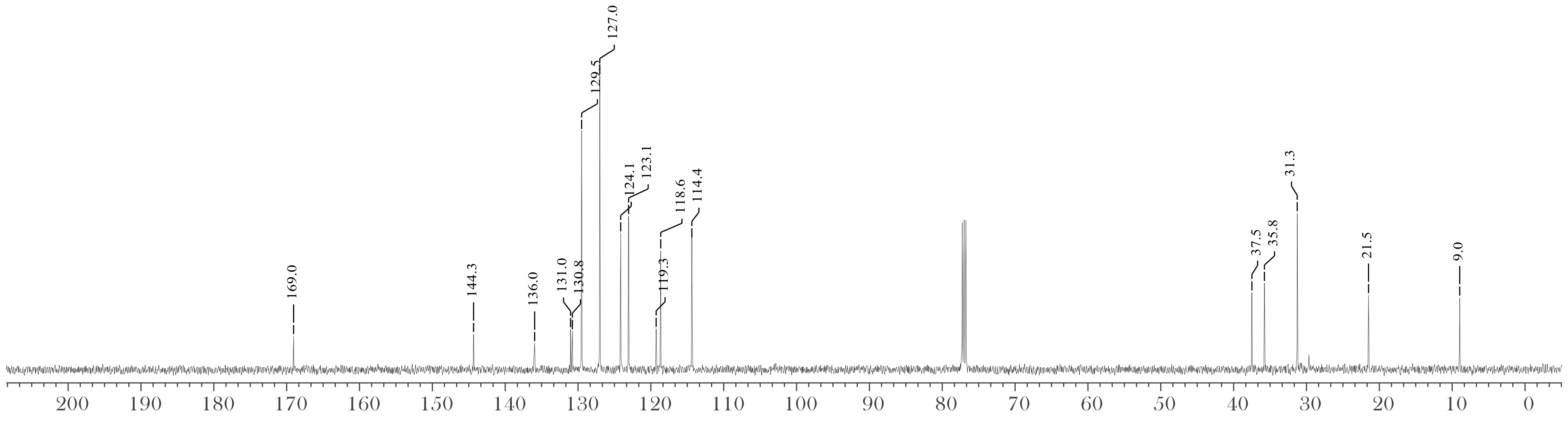

^{13}C NMR Spectrum of **11** (125 MHz, $CDCl_3$, 25 °C)

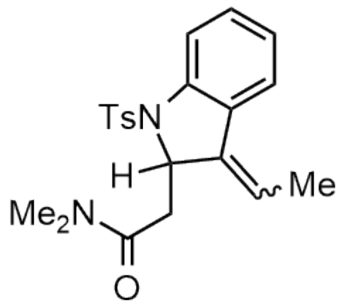

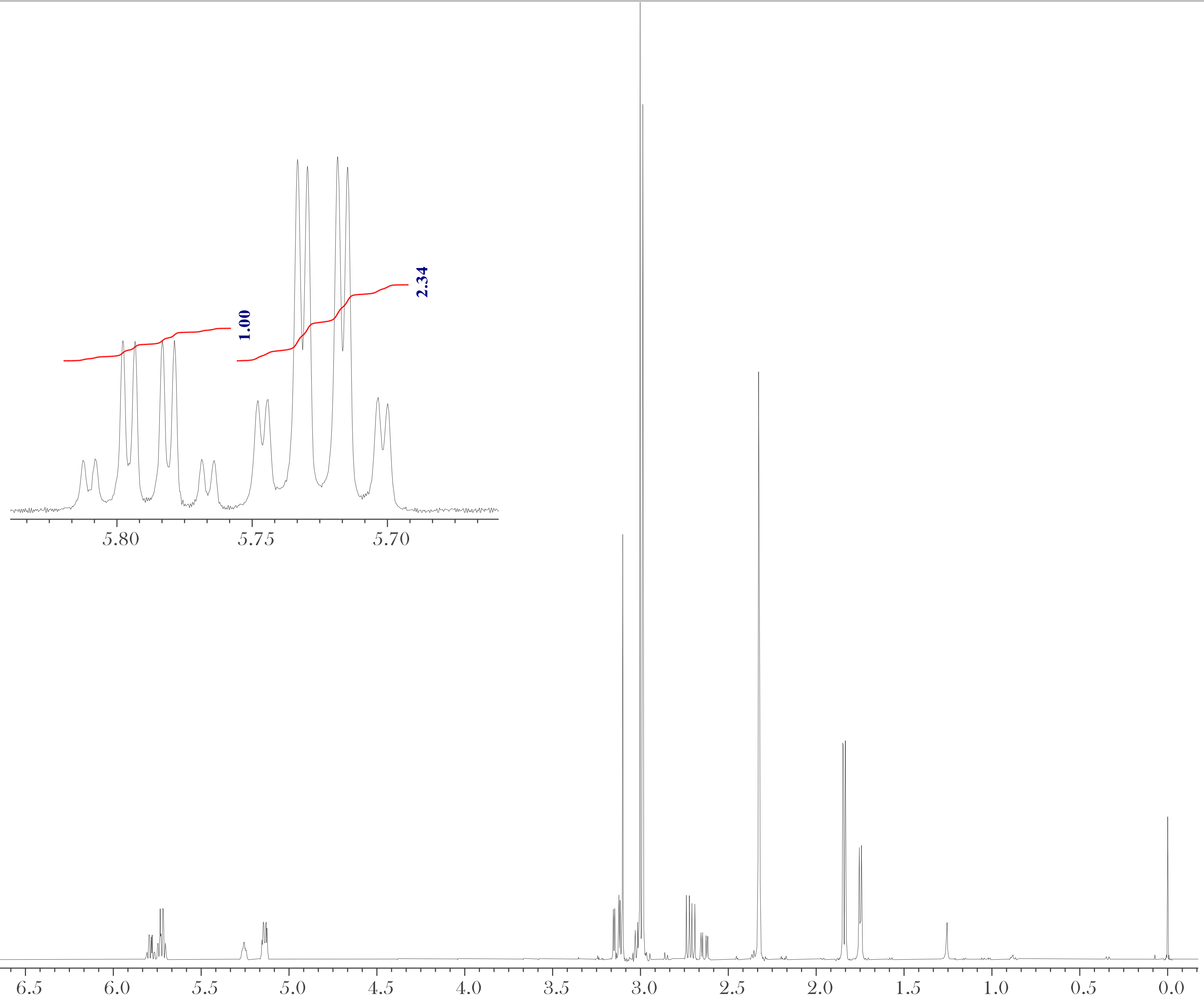

^{1}H NMR Spectrum of **13a** (E/Z = 2:1; 500 MHz, $CDCl_3$, 25 °C)

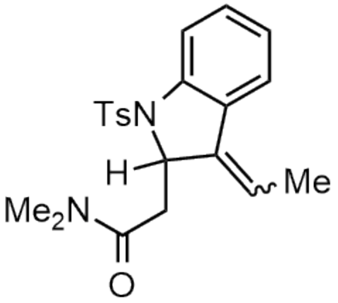

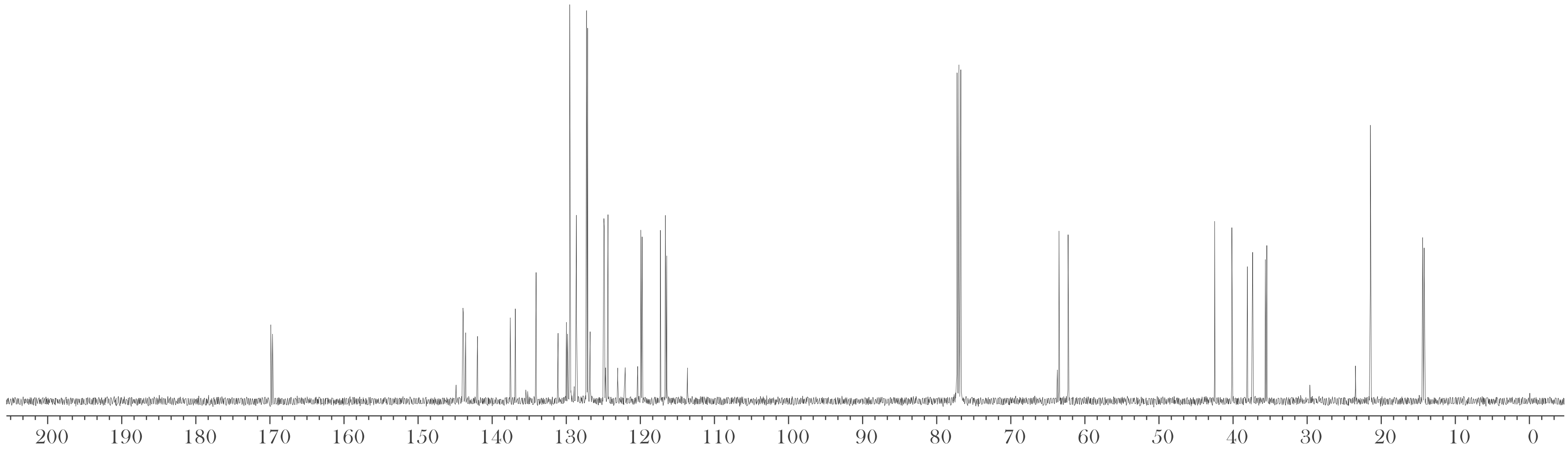

^{13}C NMR Spectrum of **13a** (E/Z = 2:1; 125 MHz, $CDCl_3$, 25 °C)

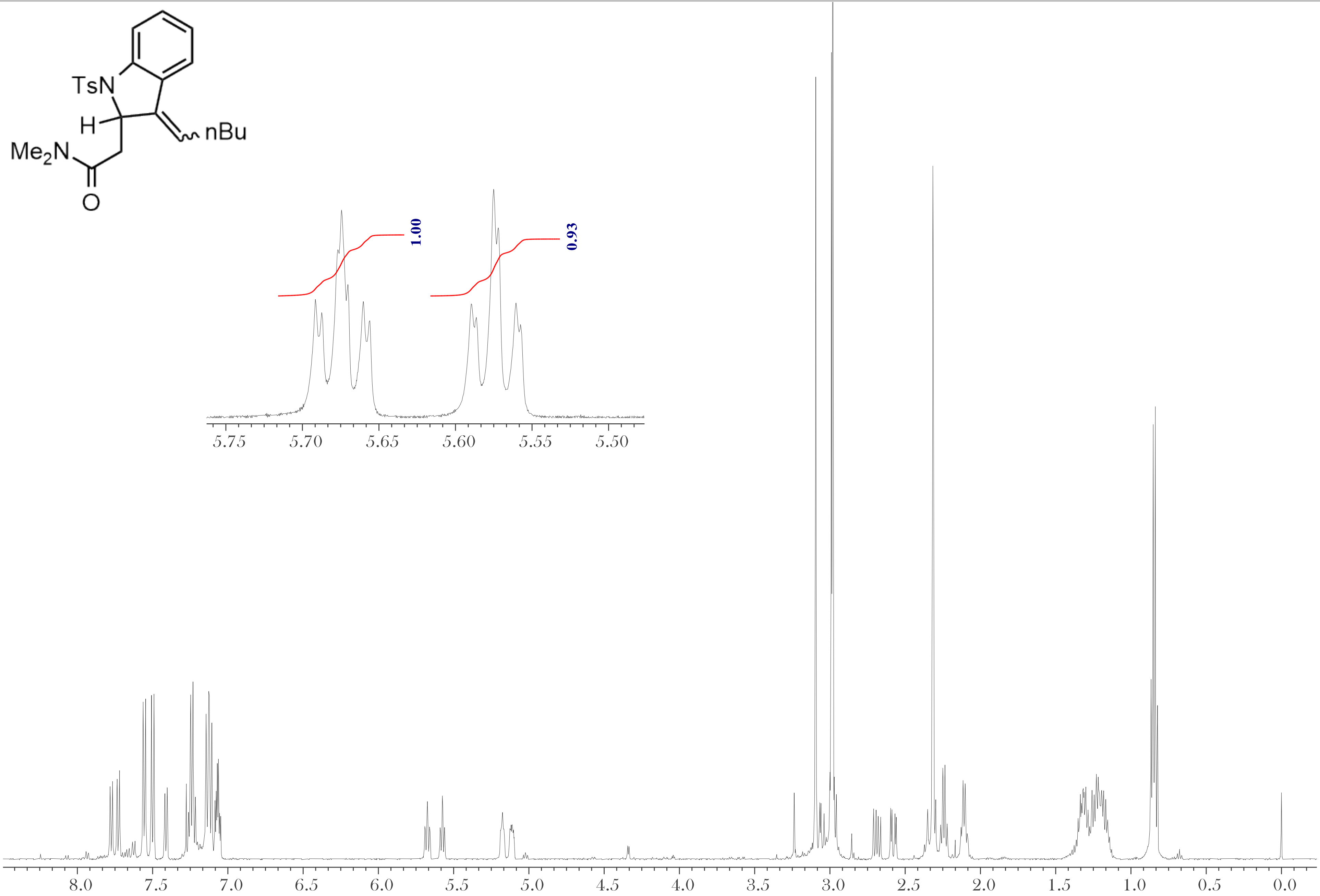

^{1}H NMR Spectrum of **13b** (E/Z = 1:1; 500 MHz, $CDCl_3$, 25 °C)

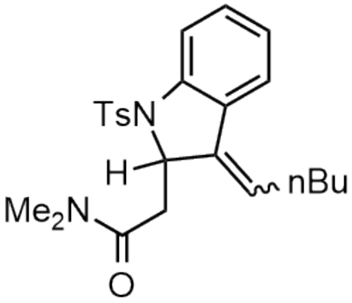

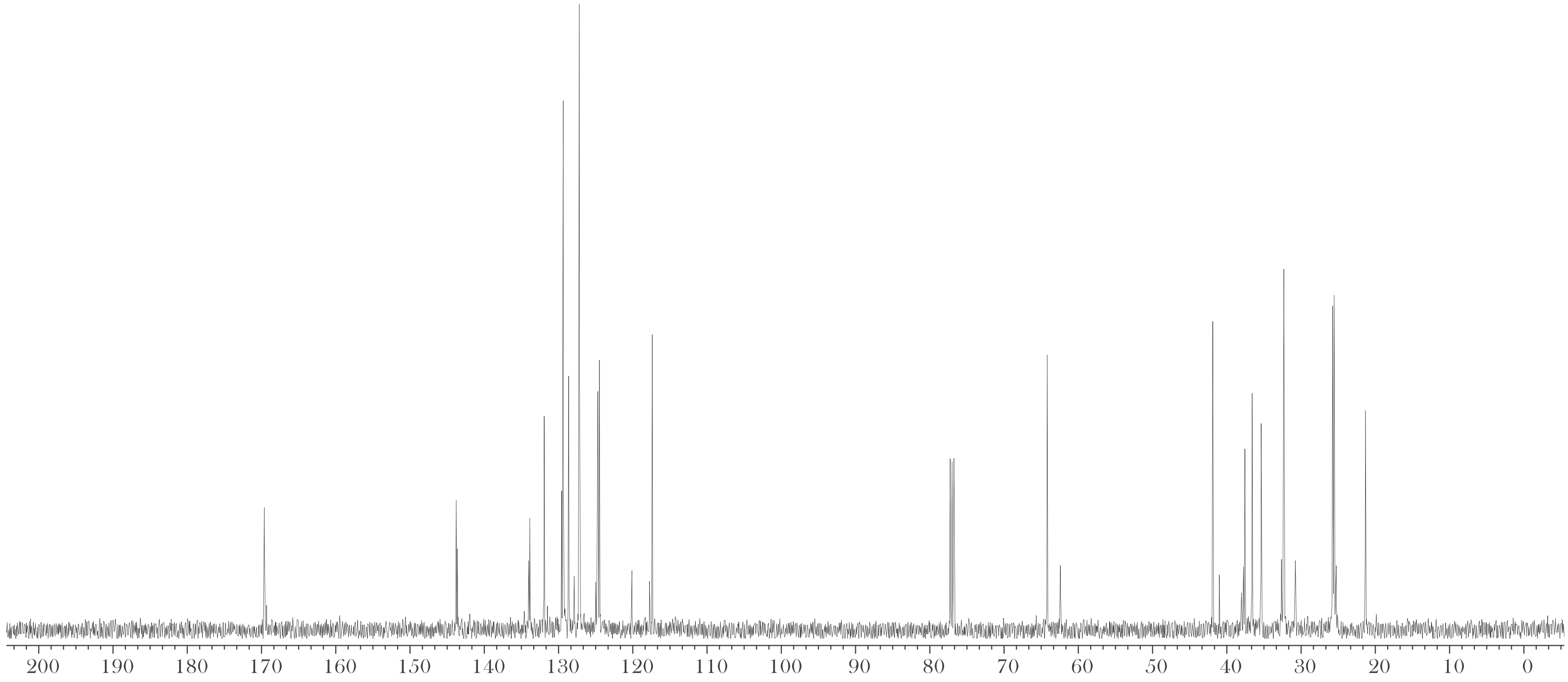

^{13}C NMR Spectrum of **13b** (E/Z = 1:1; 125 MHz, $CDCl_3$, 25 °C)

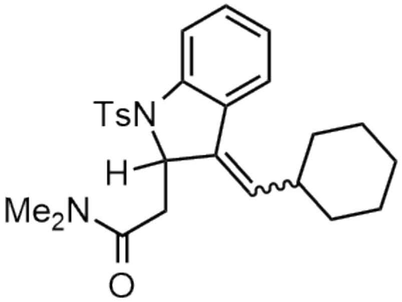

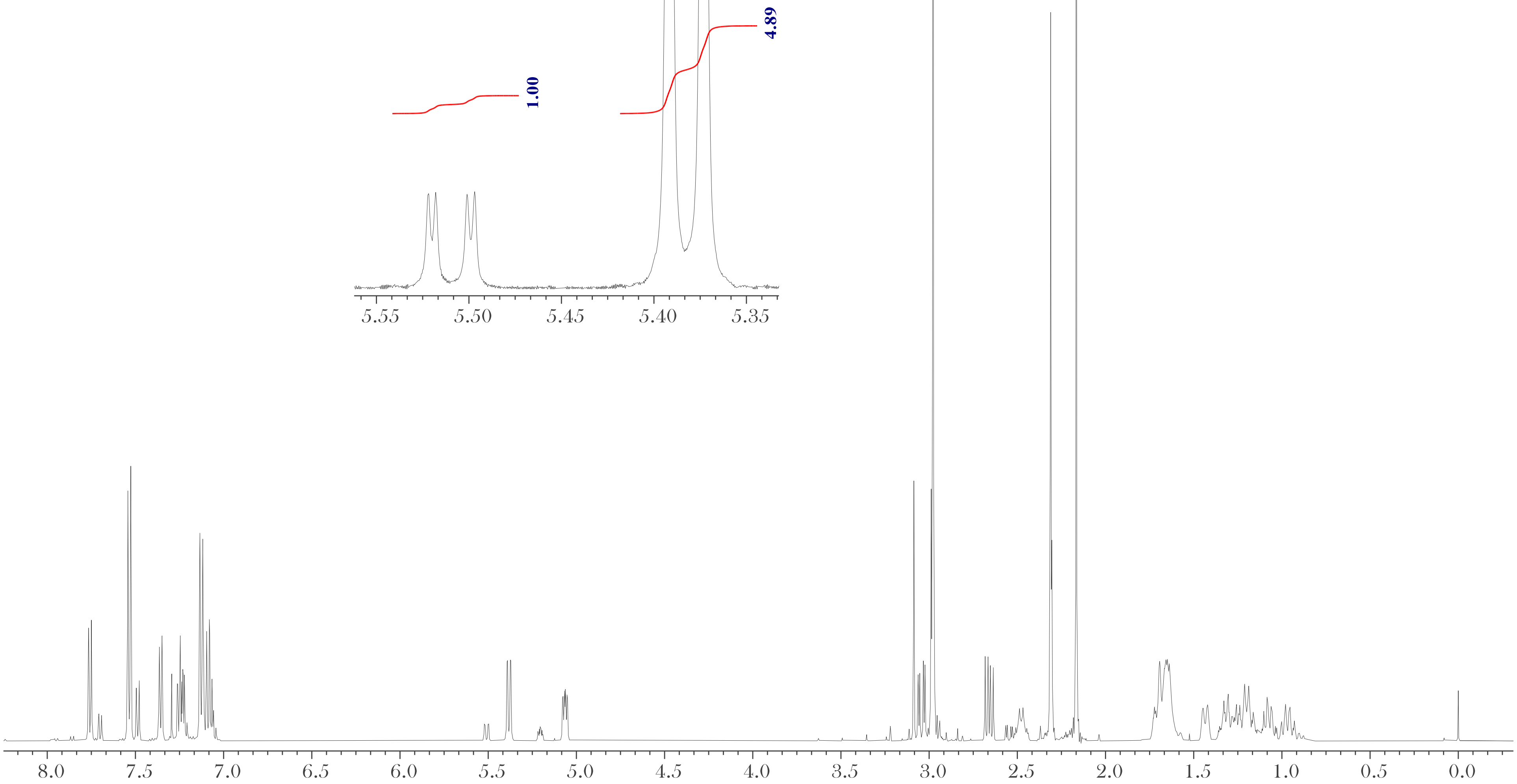

^{1}H NMR Spectrum of **13c** (E/Z = 5:1; 500 MHz, $CDCl_3$, 25 °C)

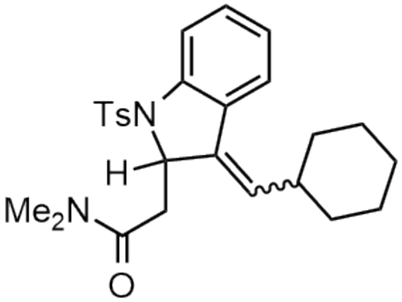

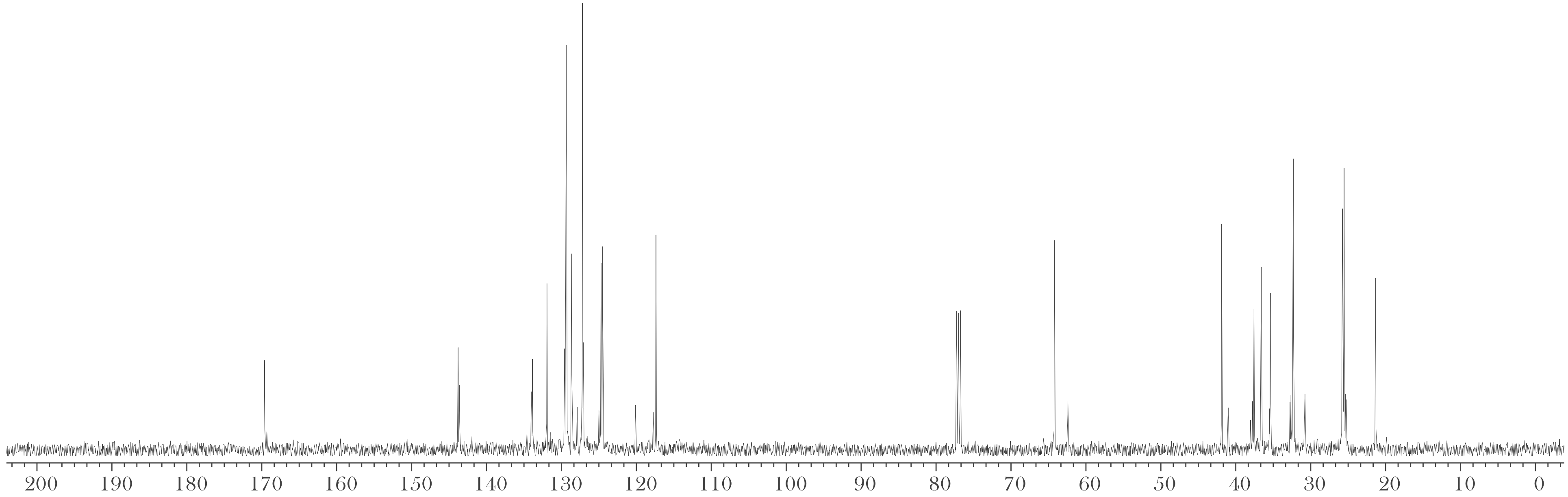

^{13}C NMR Spectrum of **13c** (E/Z = 5:1; 125 MHz, $CDCl_3$, 25 °C)

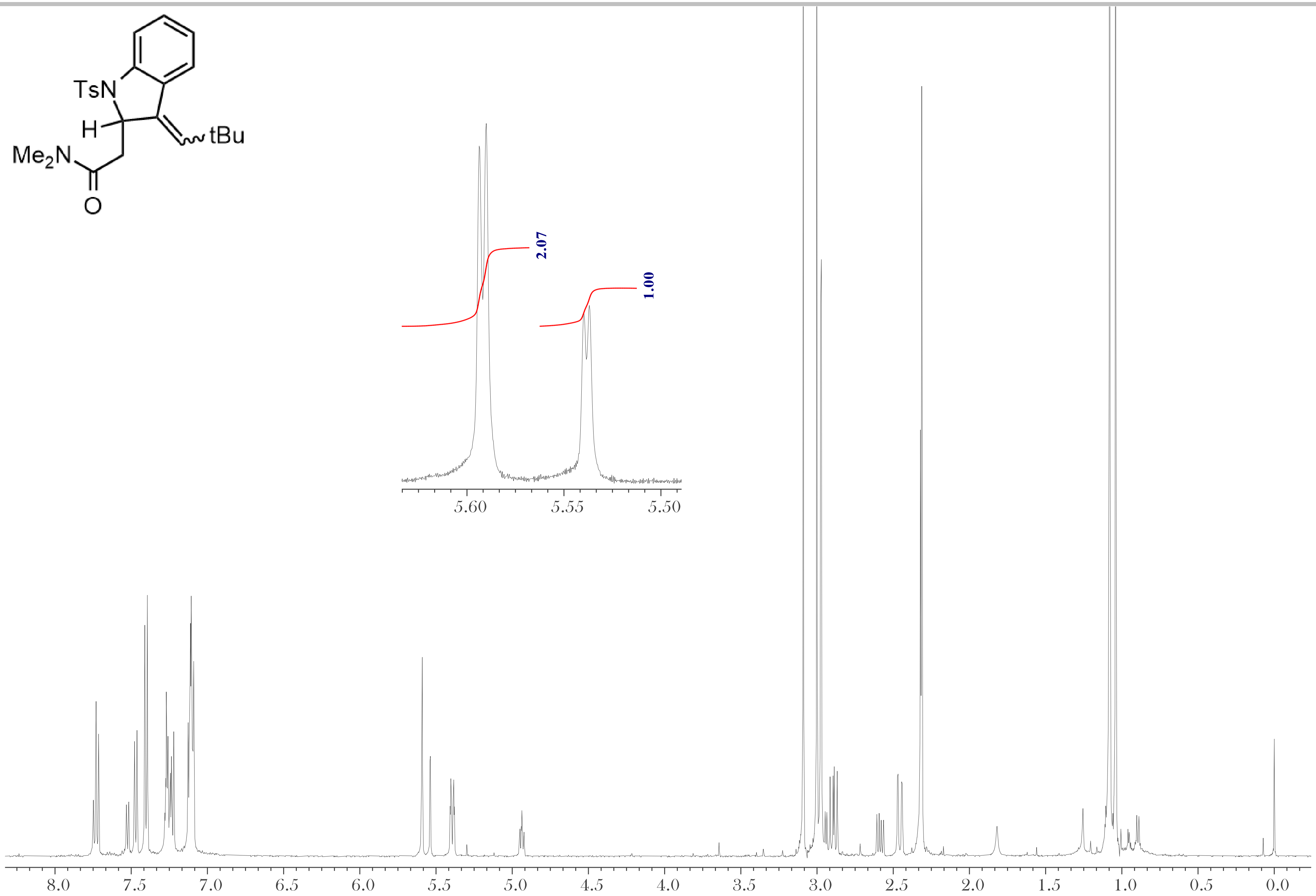

^{1}H NMR Spectrum of **13d** (E/Z = 2:1; 500 MHz, $CDCl_3$, 25 °C)

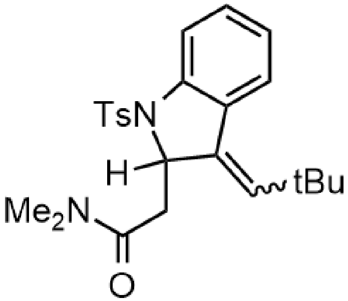

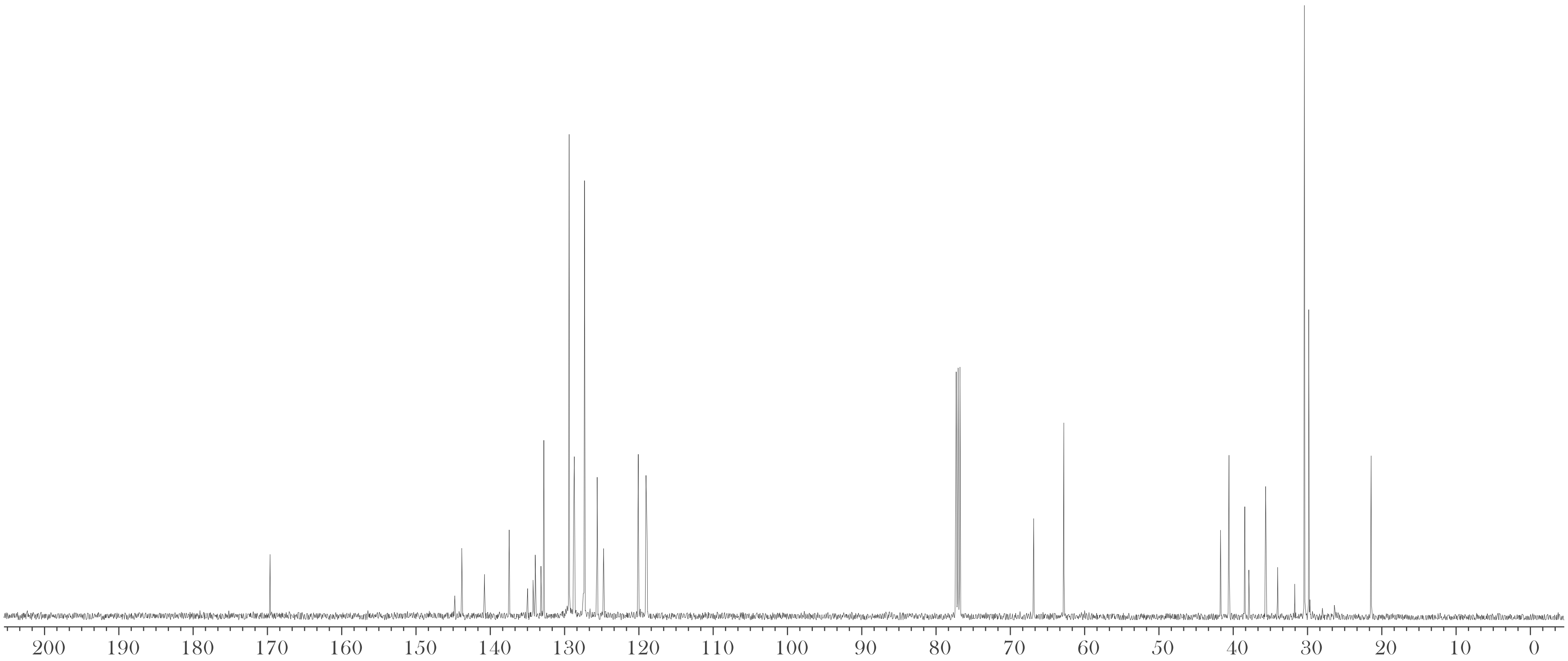

^{13}C NMR Spectrum of **13d** (E/Z = 2:1; 125 MHz, $CDCl_3$, 25 °C)

JACS JOURNAL OF THE AMERICAN CHEMICAL SOCIETY

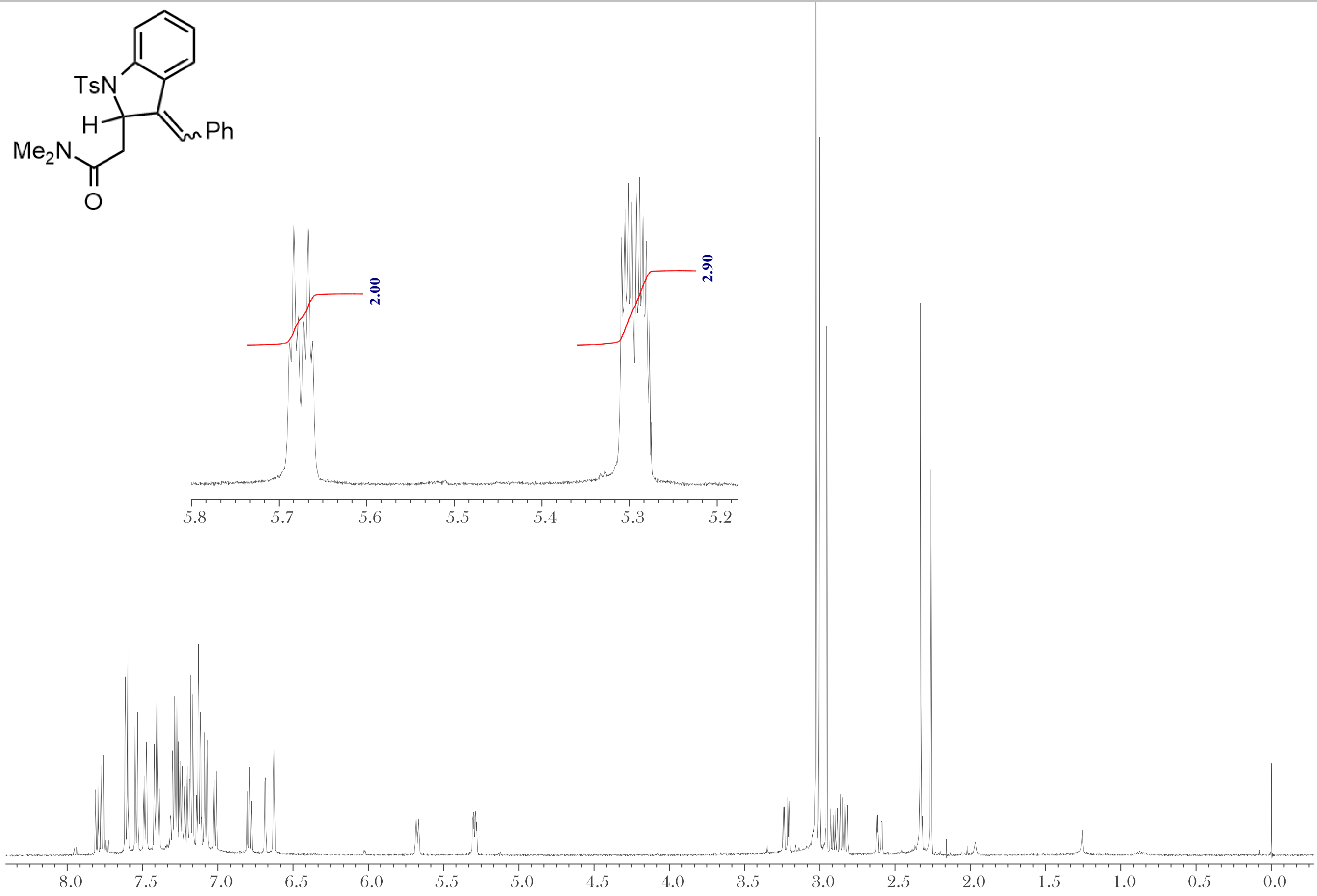

^{1}H NMR Spectrum of **13e** (E/Z = 3:2; 500 MHz, $CDCl_3$, 25 °C)

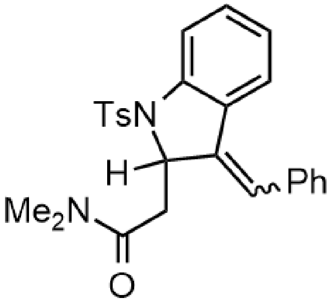

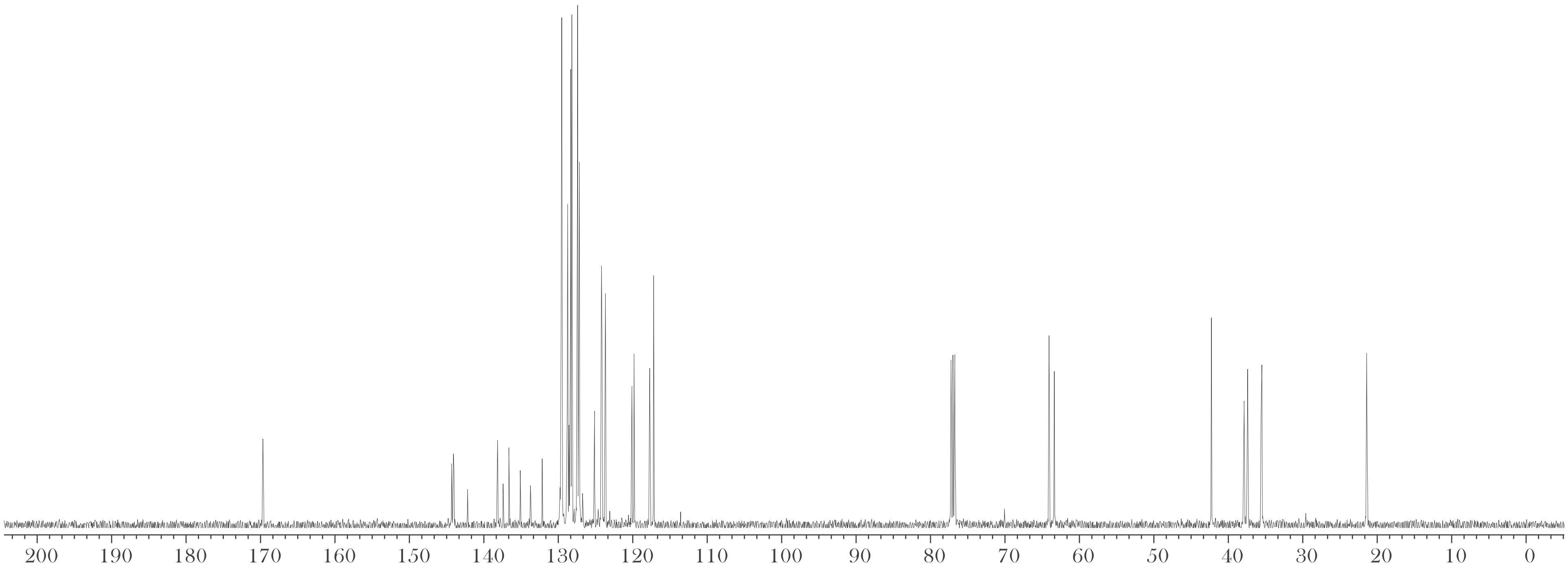

^{13}C NMR Spectrum of **13e** (E/Z = 3:2; 125 MHz, $CDCl_3$, 25 °C)

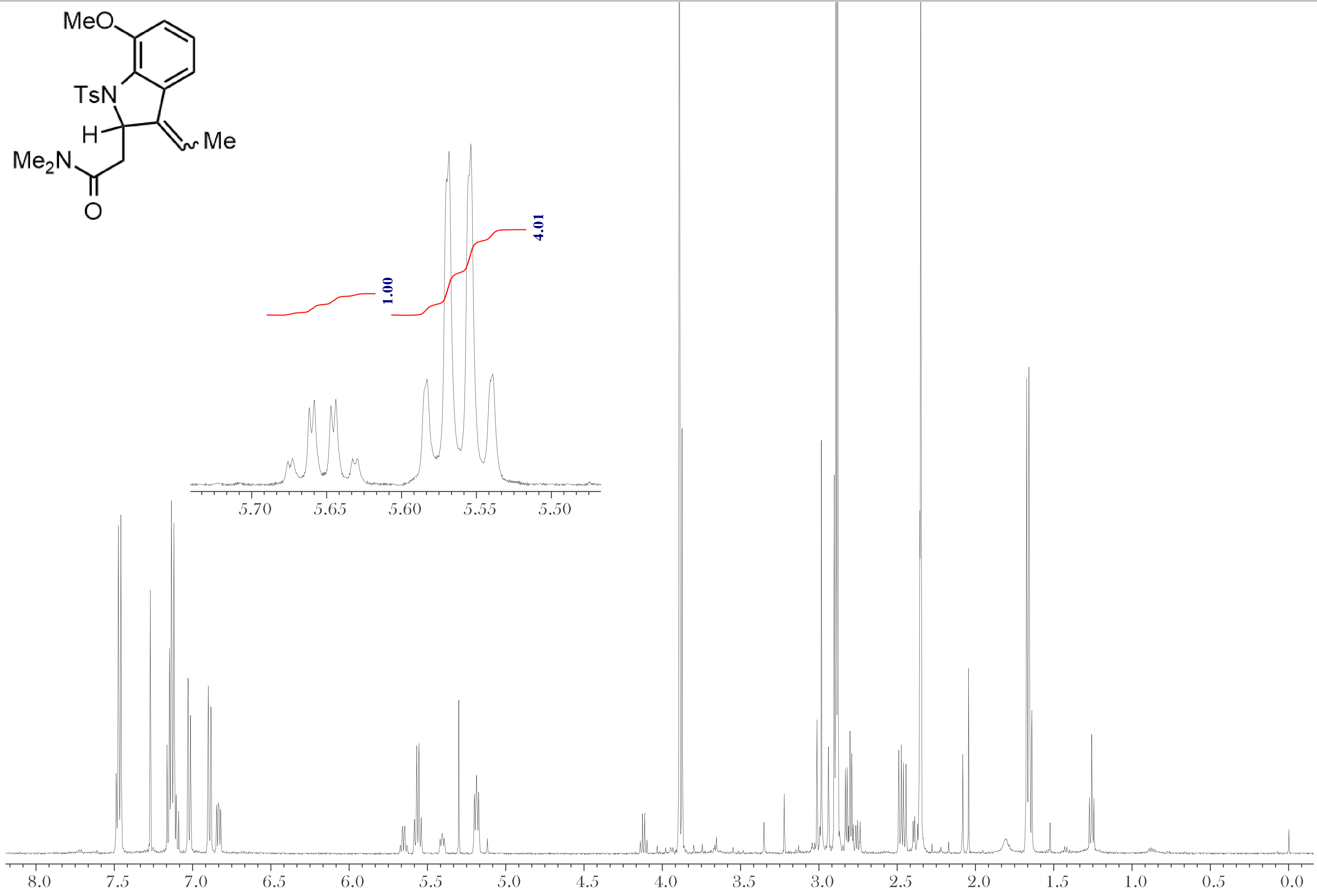

^{1}H NMR Spectrum of **13f** (E/Z = 4:1; 500 MHz, $CDCl_3$, 25 °C)

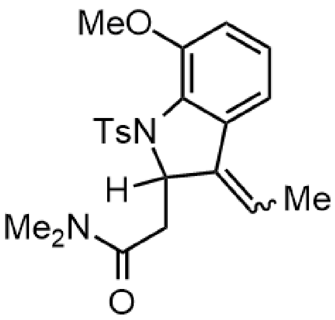

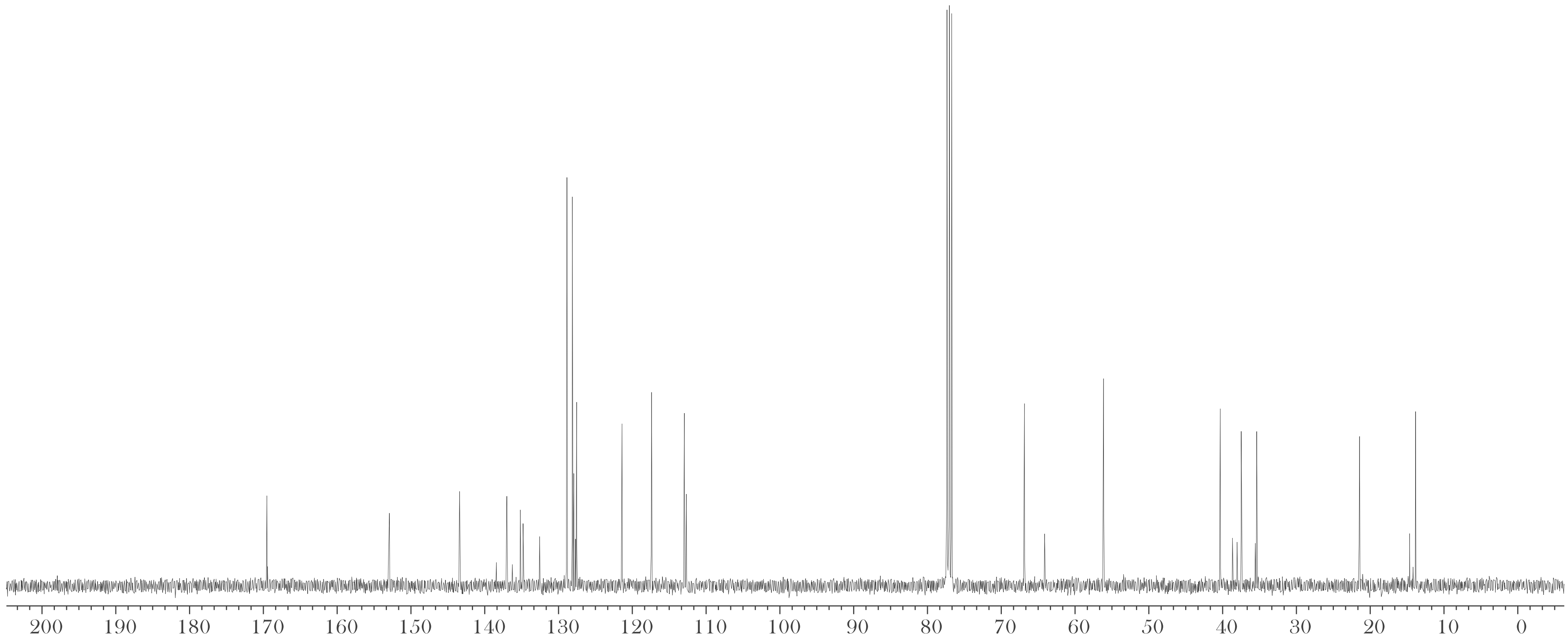

^{13}C NMR Spectrum of **13f** (E/Z = 4:1; 125 MHz, $CDCl_3$, 25 °C)

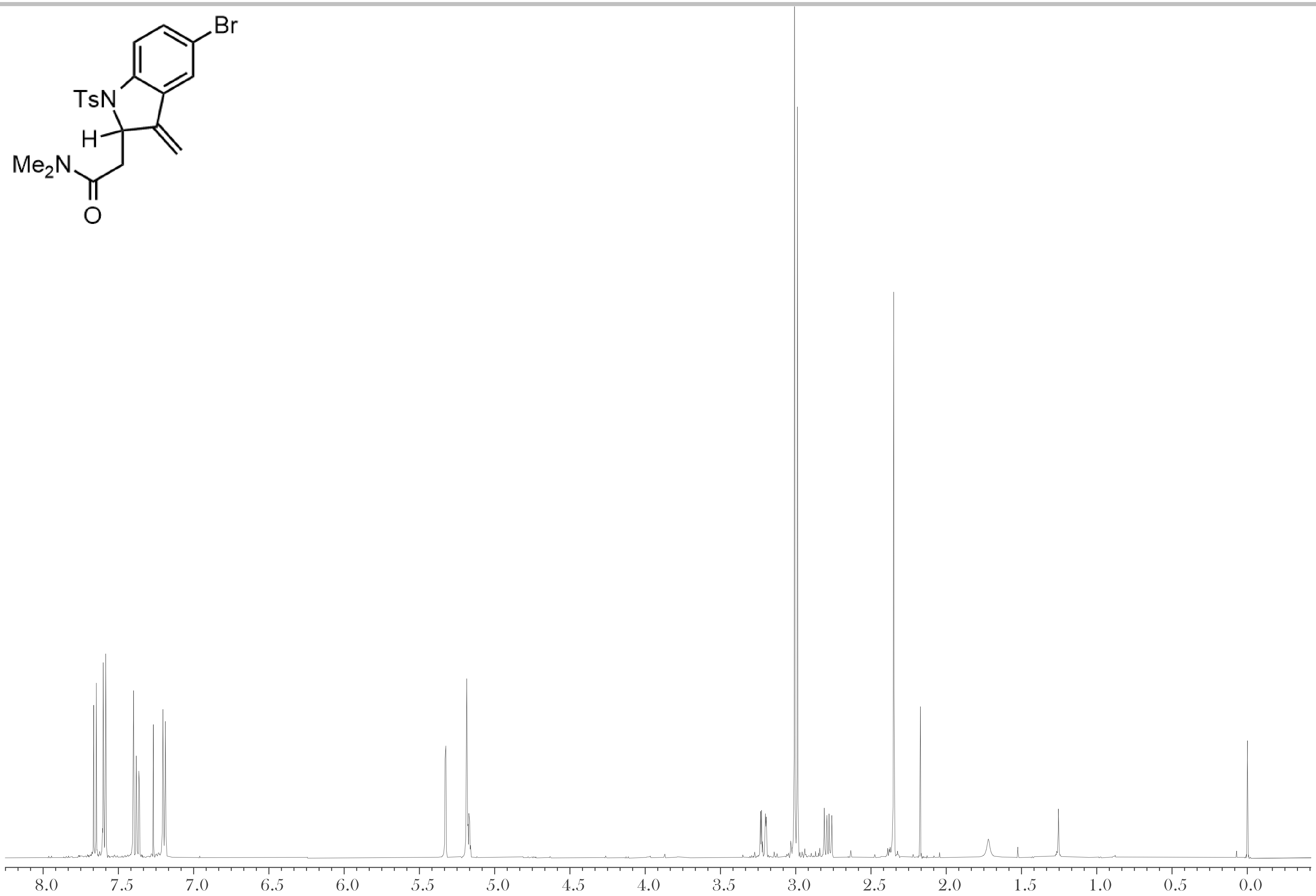

^{1}H NMR Spectrum of **13g** (500 MHz, $CDCl_3$, 25 °C)

JACS JOURNAL OF THE AMERICAN CHEMICAL SOCIETY

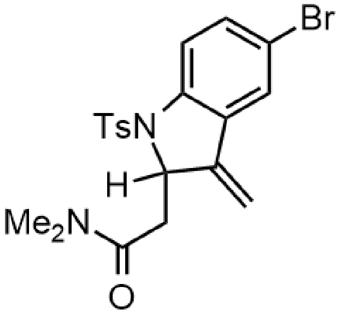

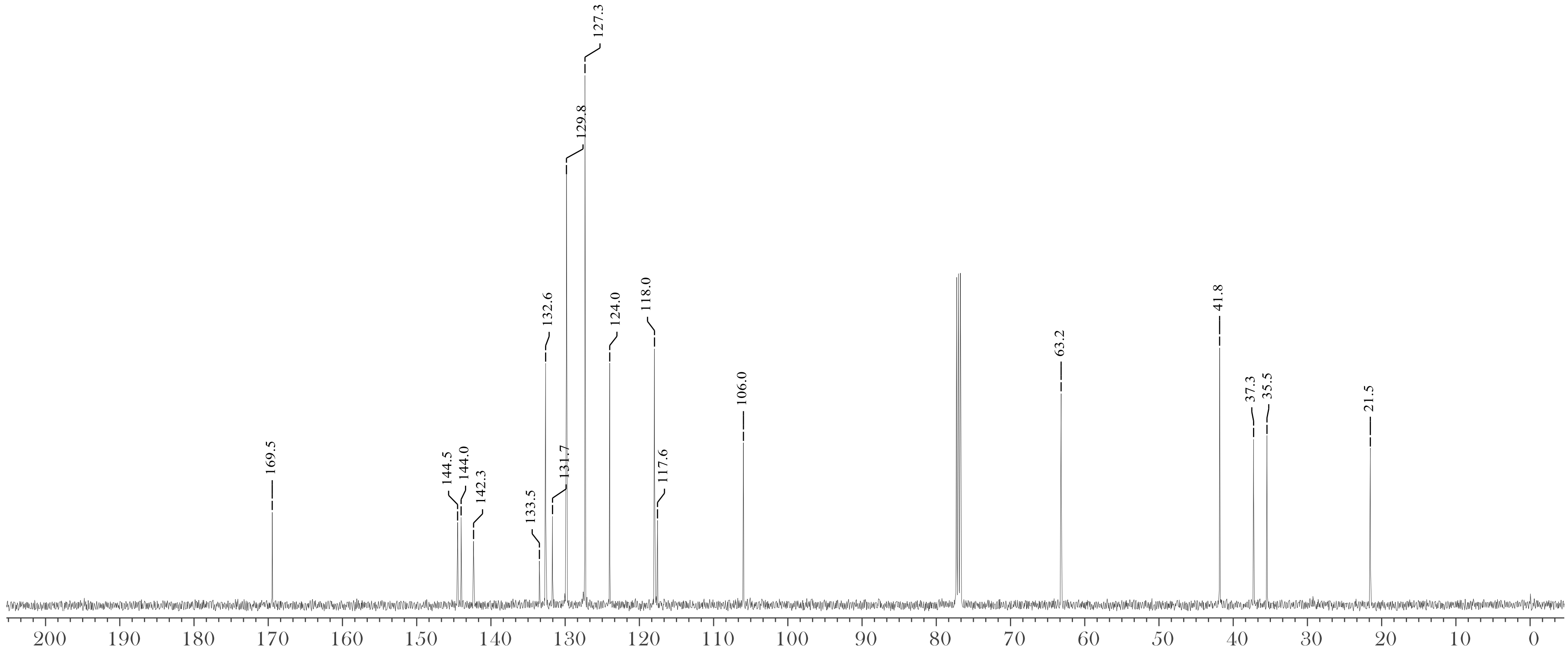

^{13}C NMR Spectrum of **13g** (125 MHz, $CDCl_3$, 25 °C)

JACS JOURNAL OF THE AMERICAN CHEMICAL SOCIETY

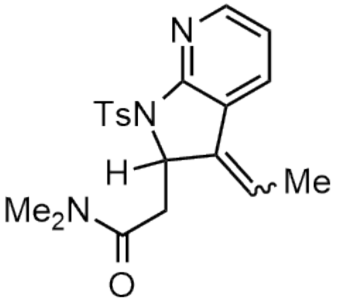

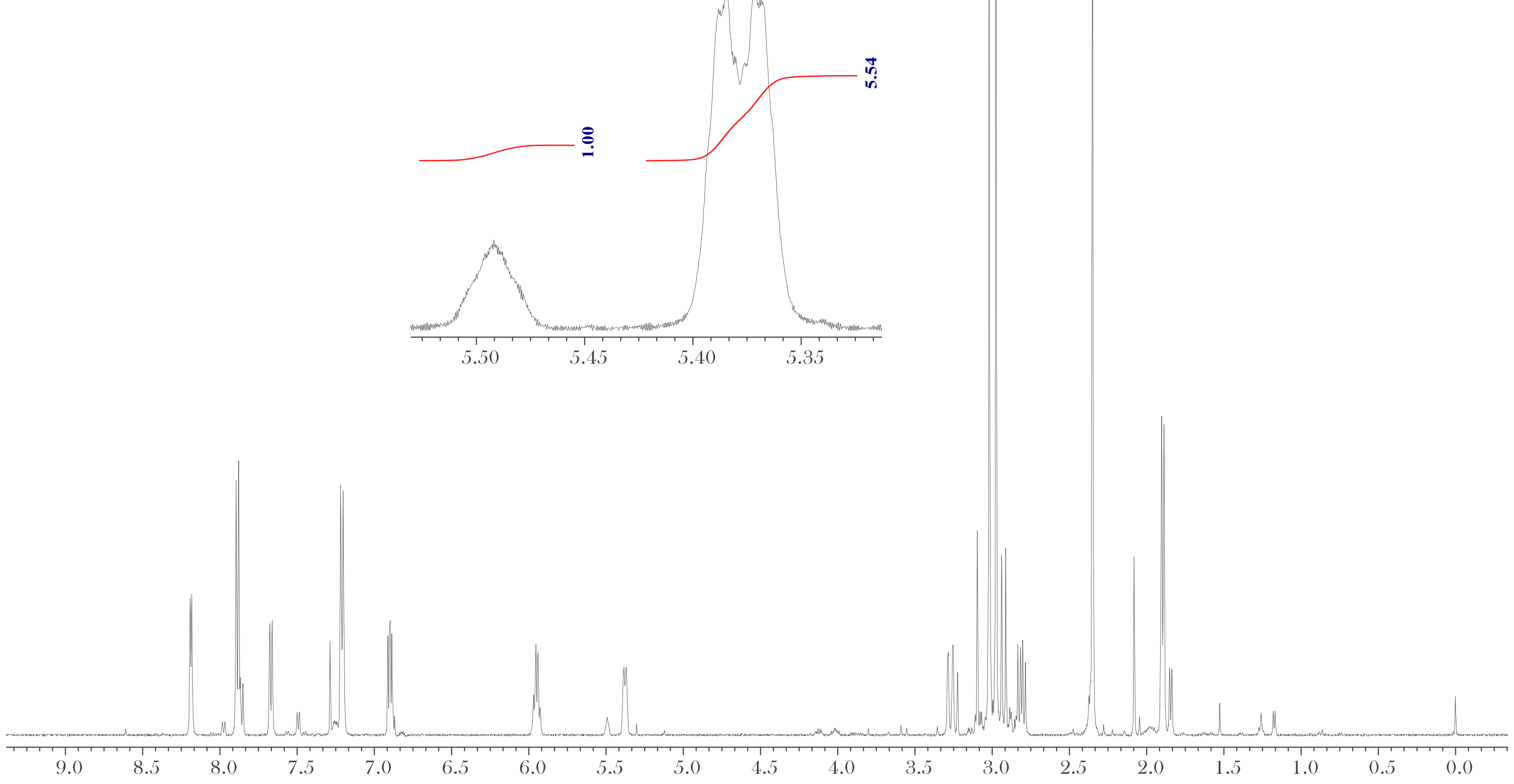

^{1}H NMR Spectrum of **13h** (E/Z = 5:1; 500 MHz, $CDCl_3$, 25 °C)

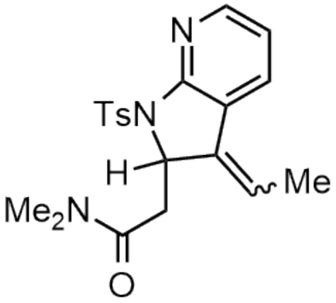

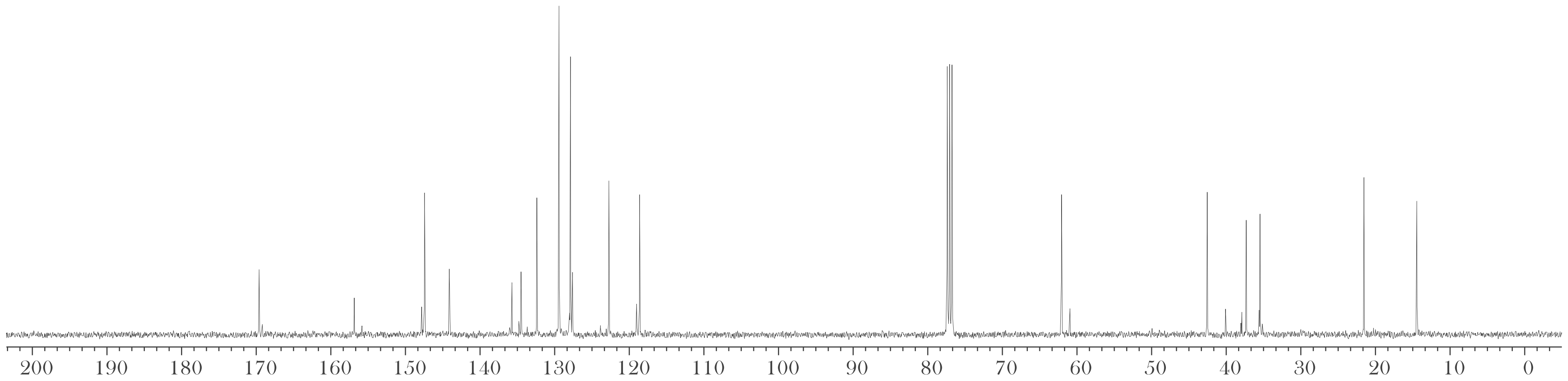

^{13}C NMR Spectrum of **13h** (E/Z = 5:1; 125 MHz, $CDCl_3$, 25 °C)

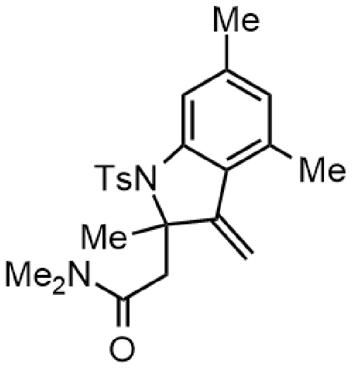

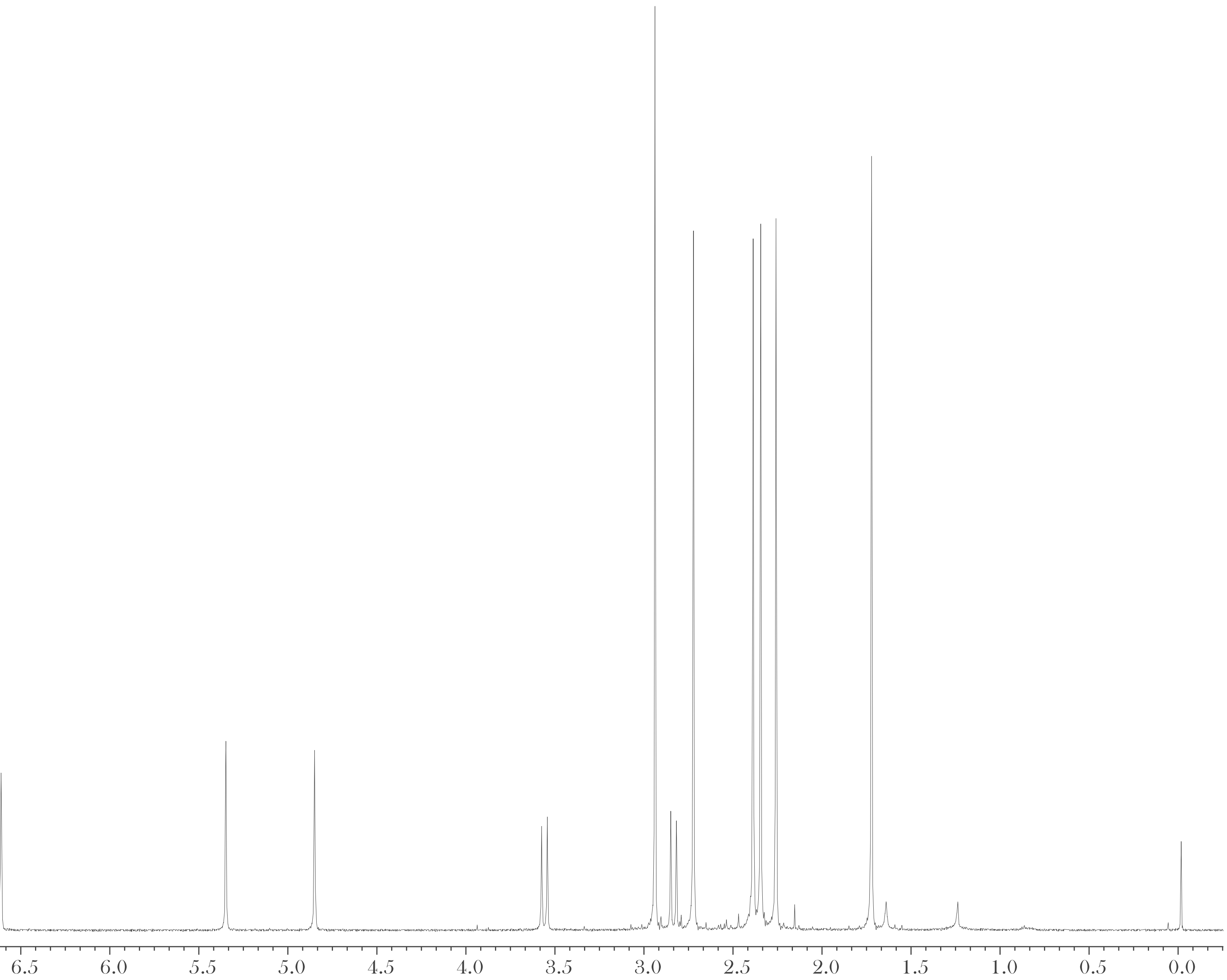

^{1}H NMR Spectrum of **13i** (500 MHz, $CDCl_3$, 25 °C)

JACS
JOURNAL OF THE AMERICAN CHEMICAL SOCIETY

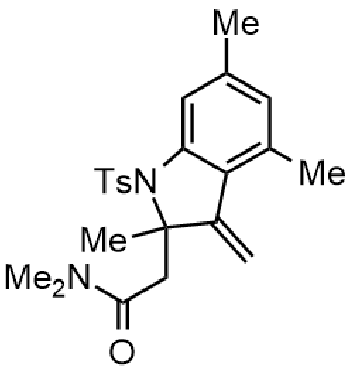

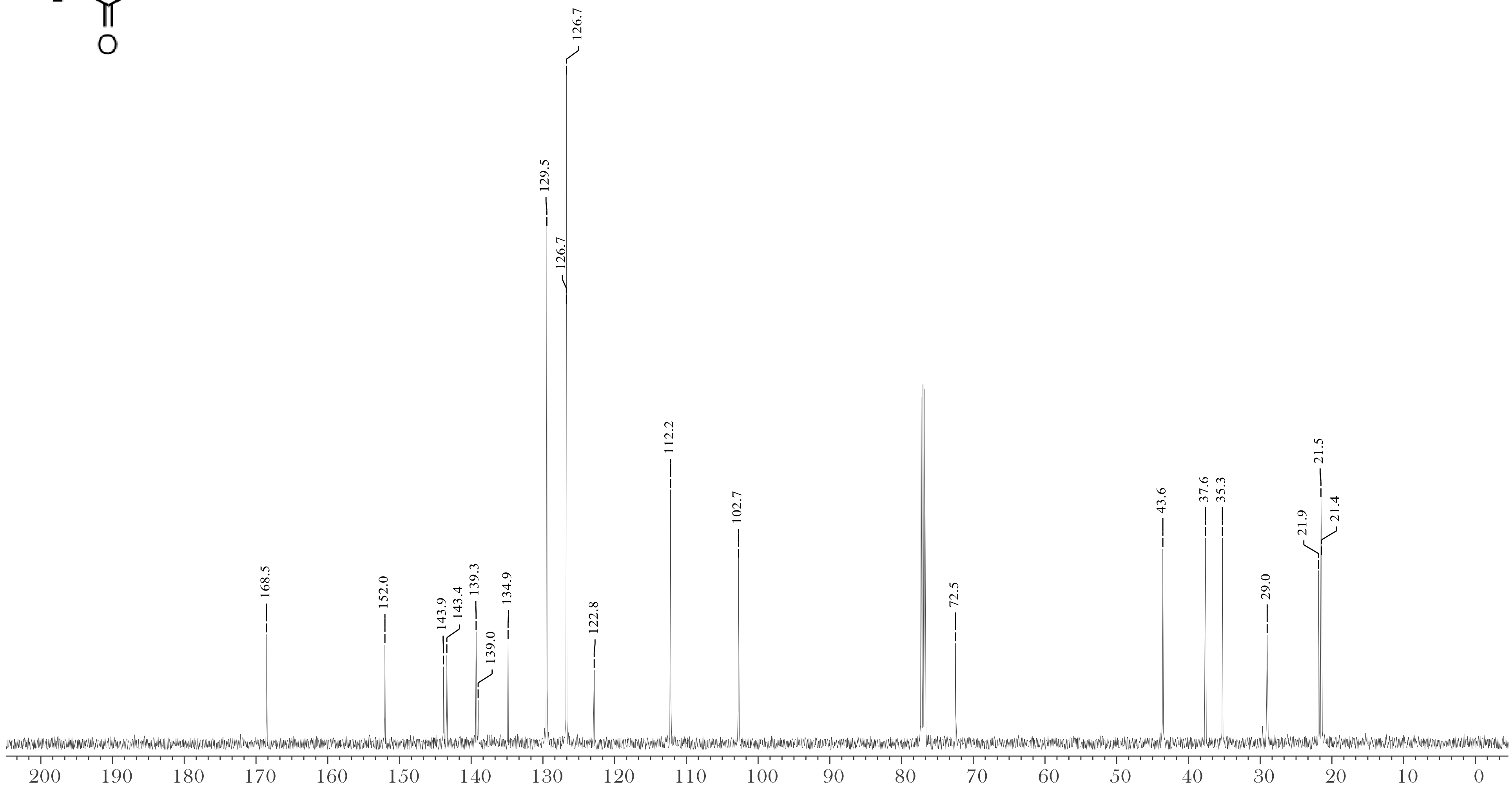

^{13}C NMR Spectrum of **13i** (125 MHz, $CDCl_3$, 25 °C)

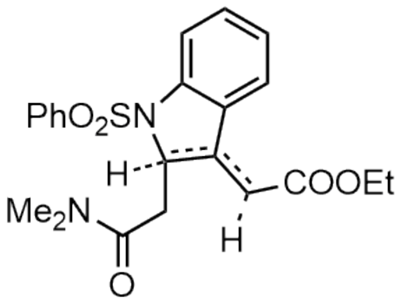

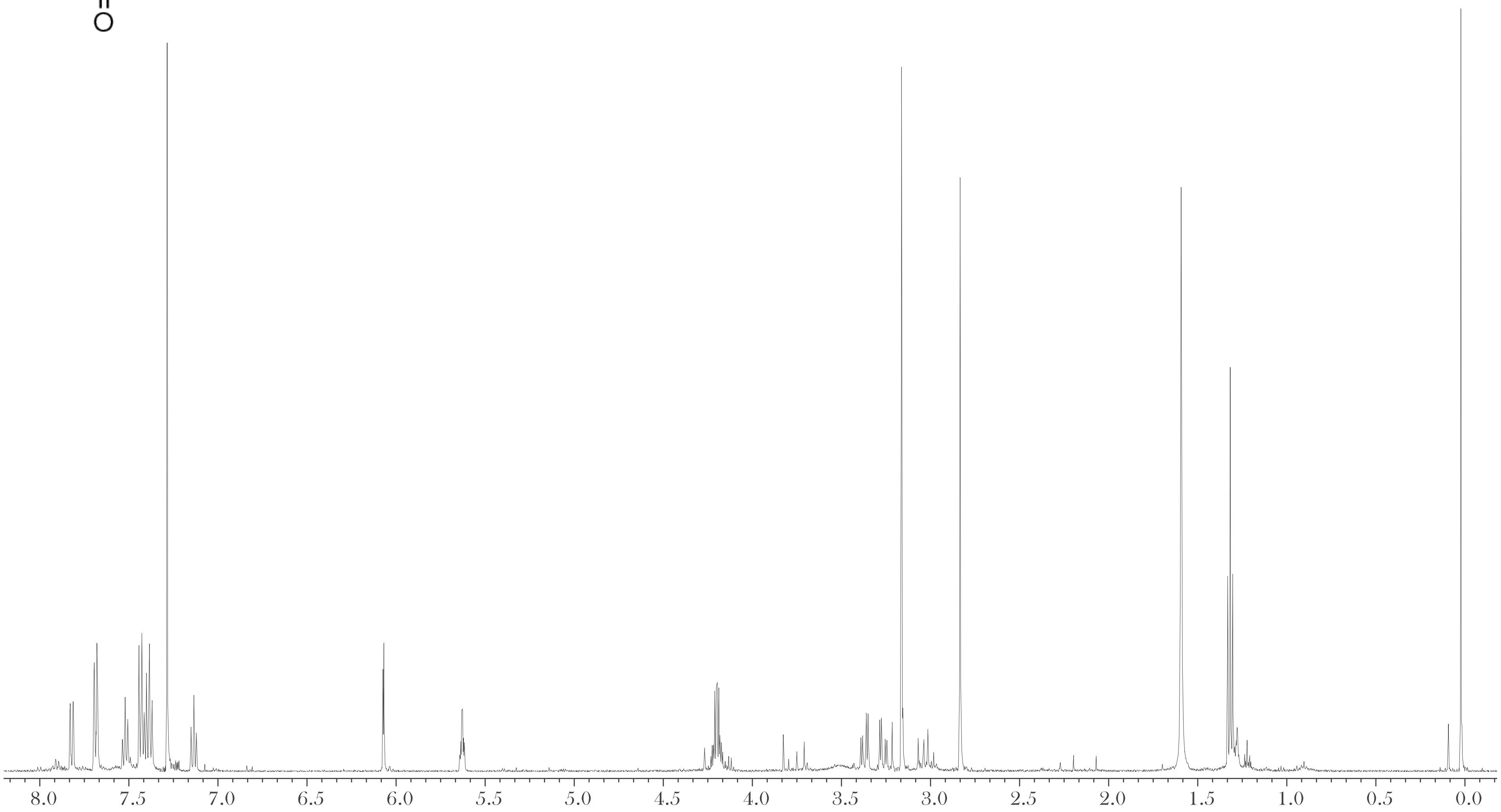

^{1}H NMR Spectrum of **13j** (E only, indoline/indole = 10:1; 500 MHz, $CDCl_3$, 25 °C)

JACS JOURNAL OF THE AMERICAN CHEMICAL SOCIETY

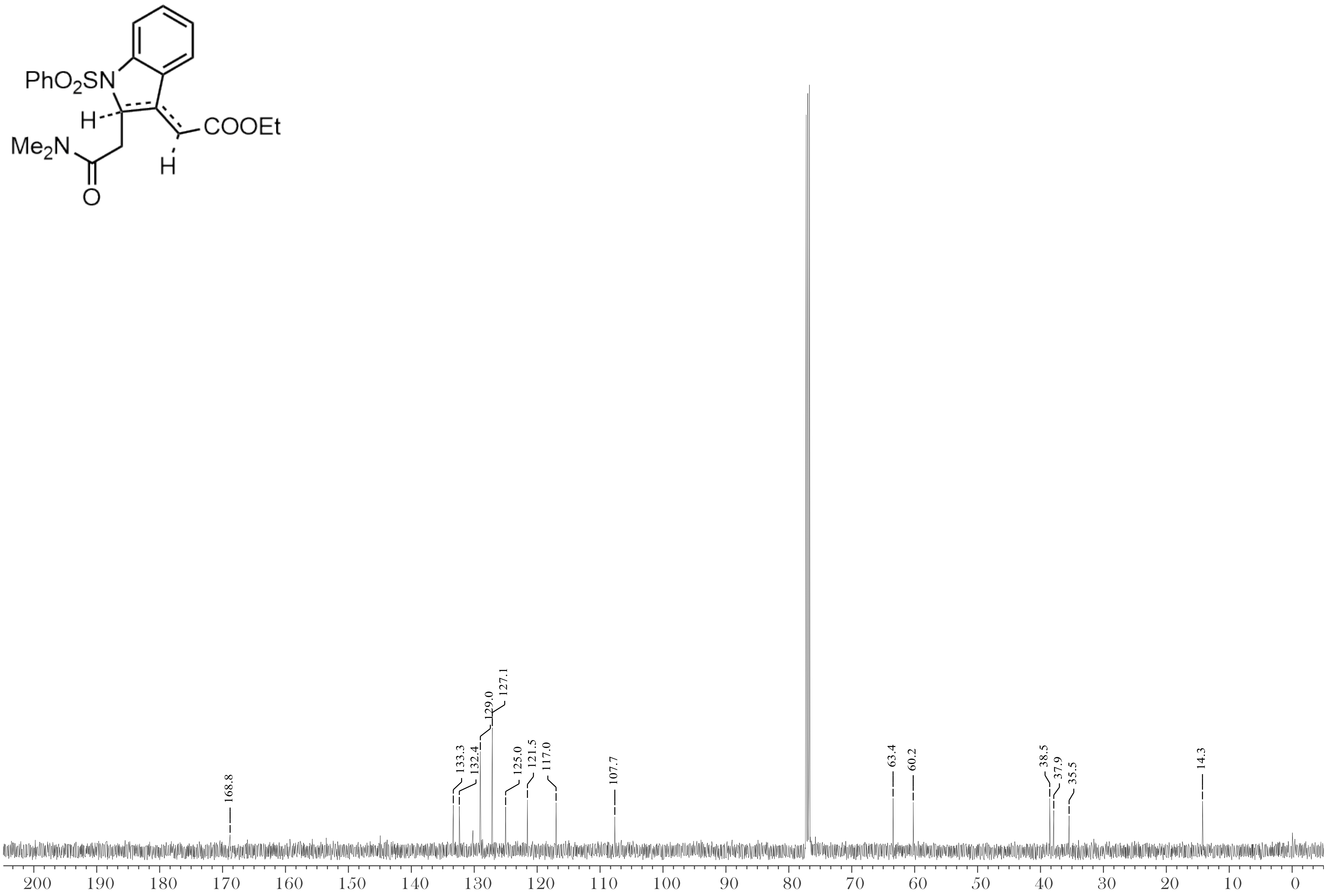

^{13}C NMR Spectrum of **13j** (E only, indoline/indole = 10:1; 125 MHz, $CDCl_3$, 25 °C)

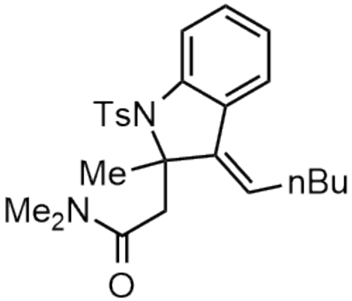

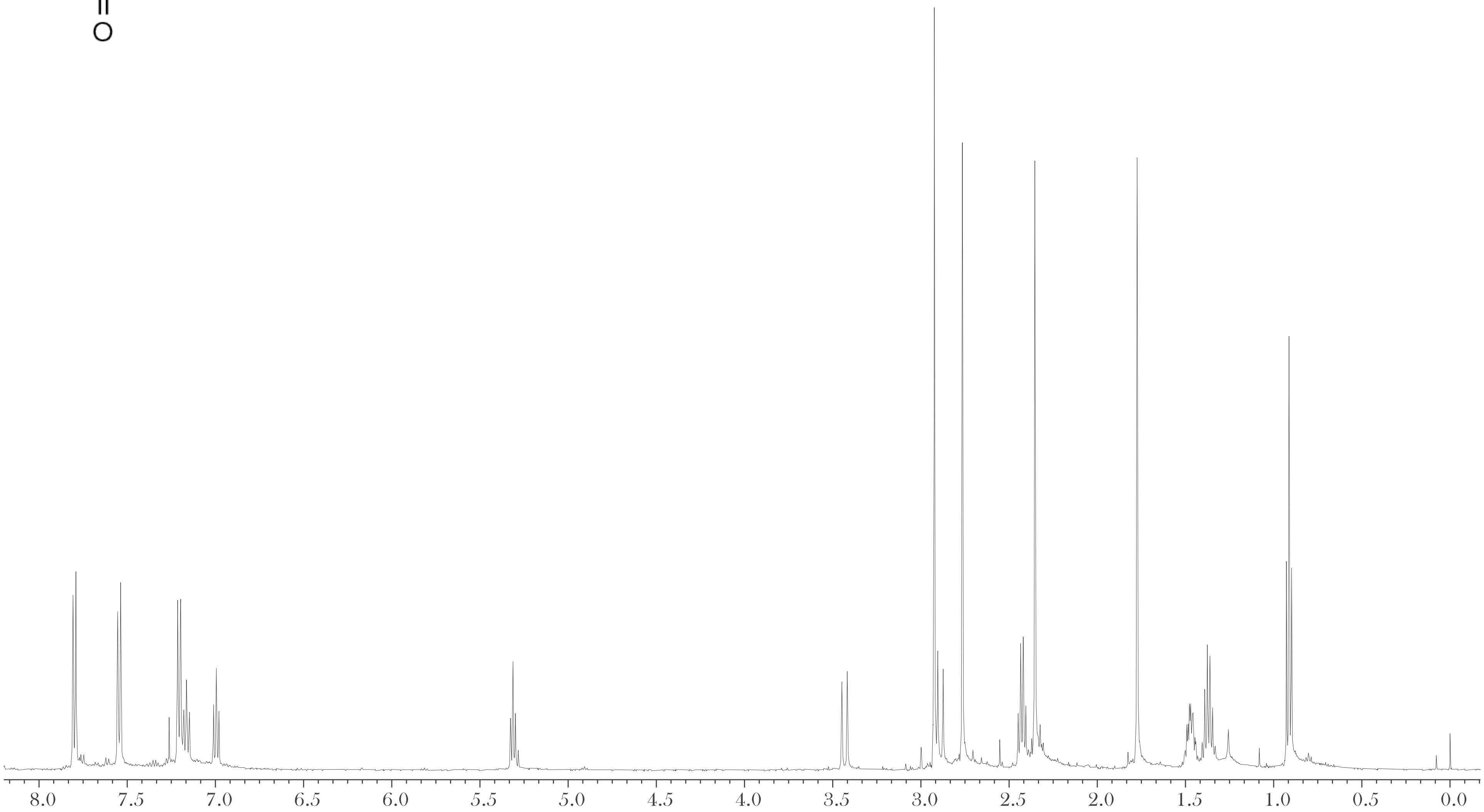

^{1}H NMR Spectrum of **13k** (500 MHz, $CDCl_3$, 25 °C)

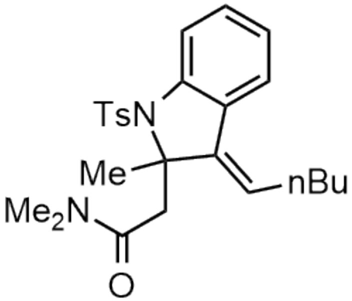

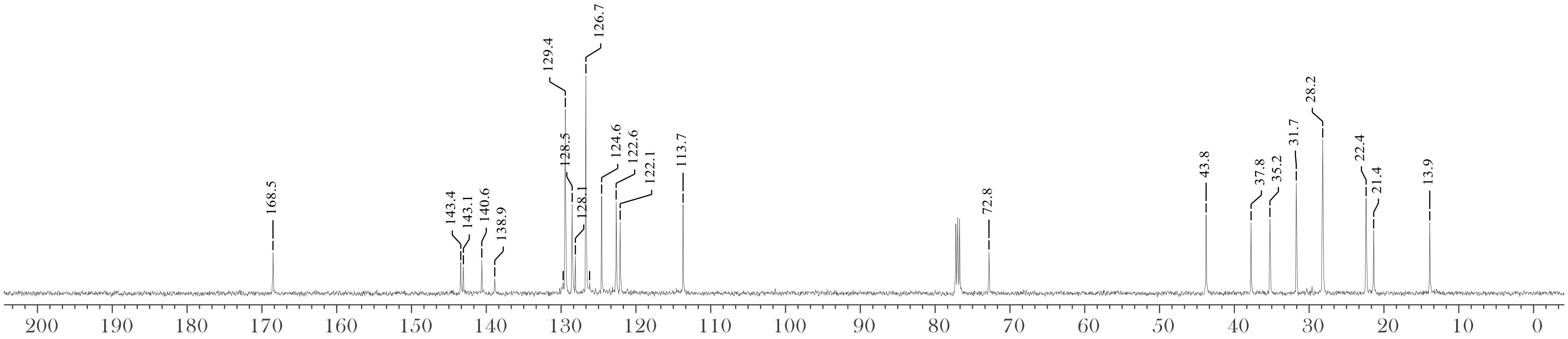

^{13}C NMR Spectrum of **13k** (125 MHz, $CDCl_3$, 25 °C)

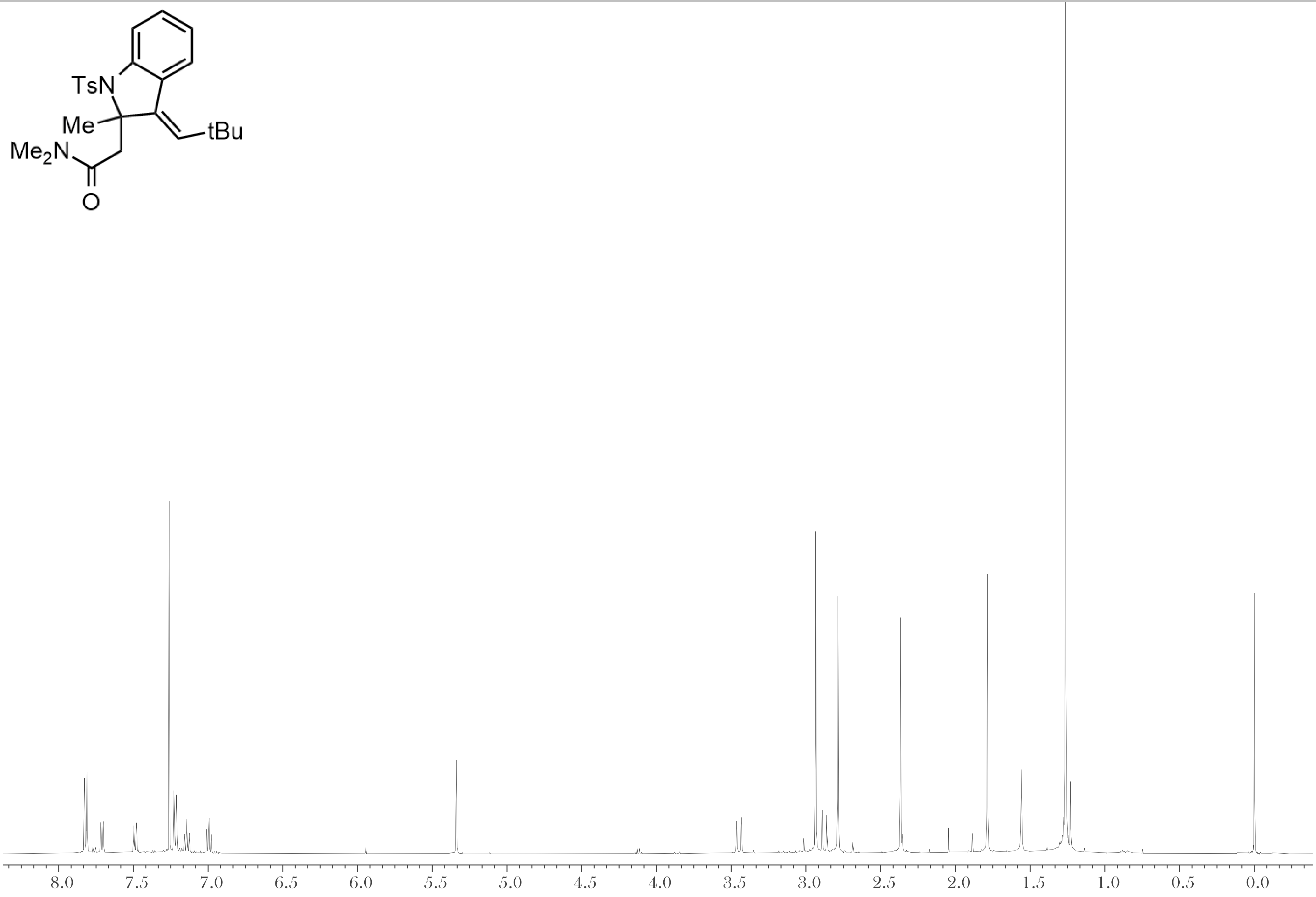

^{1}H NMR Spectrum of **13l** (500 MHz, $CDCl_3$, 25 °C)

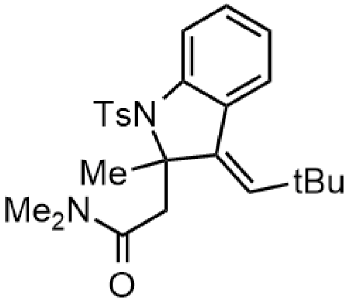

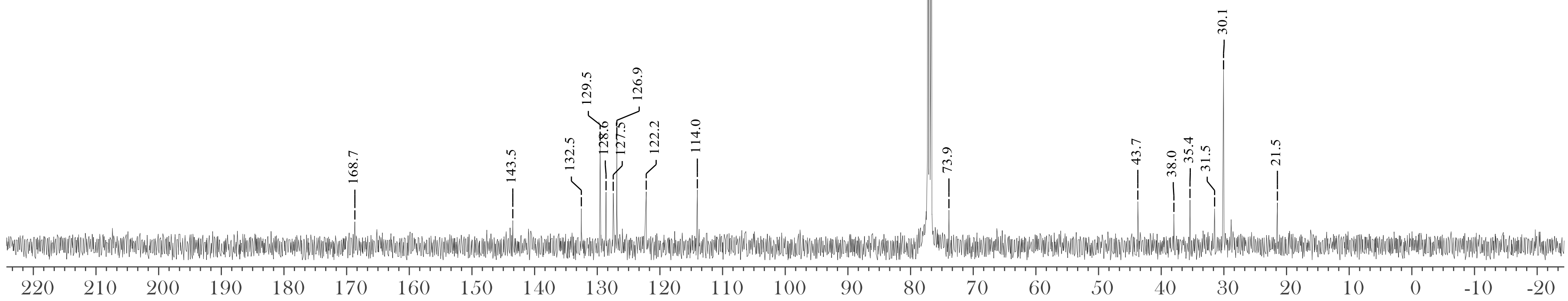

^{1}H NMR Spectrum of **13l** (100 MHz, $CDCl_3$, 25 °C)

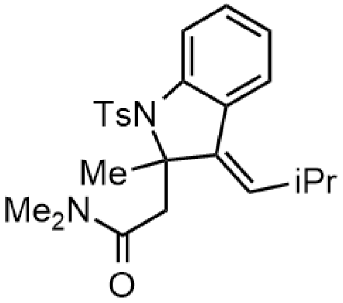

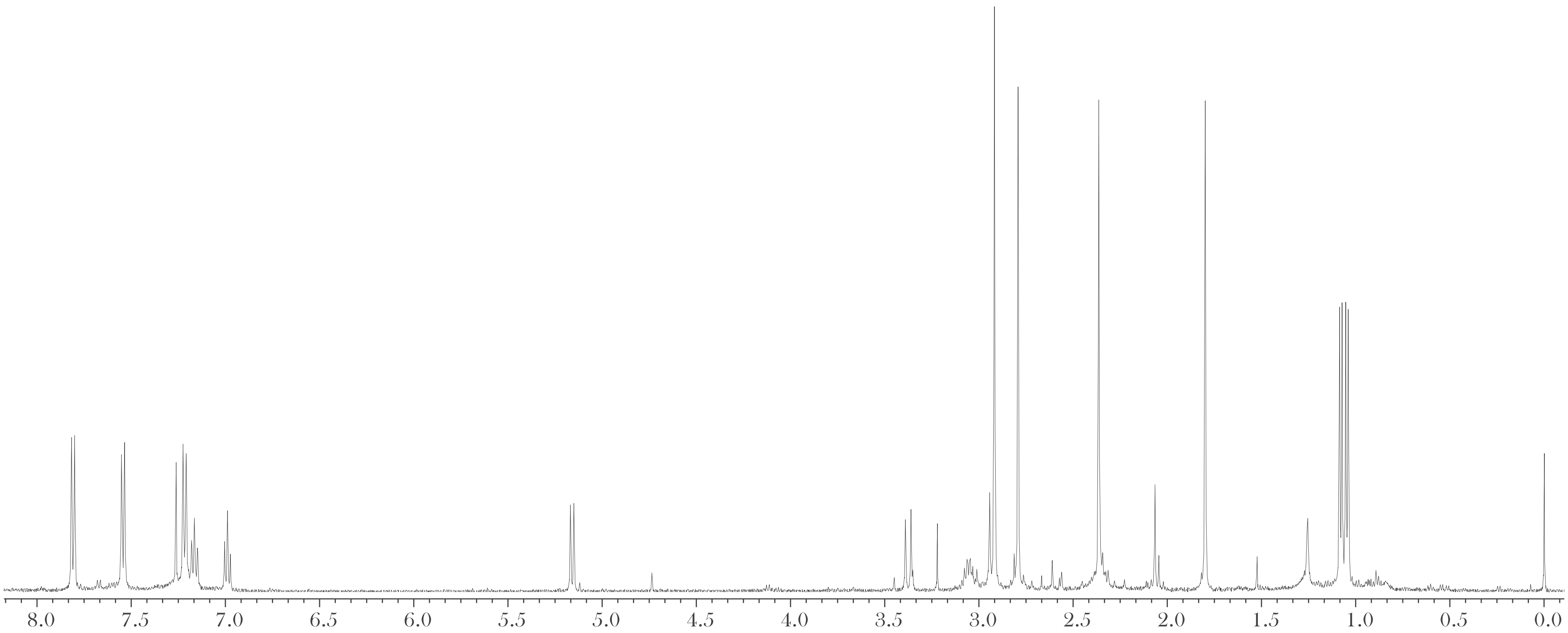

^{1}H NMR Spectrum of **13m** (500 MHz, $CDCl_3$, 25 °C)

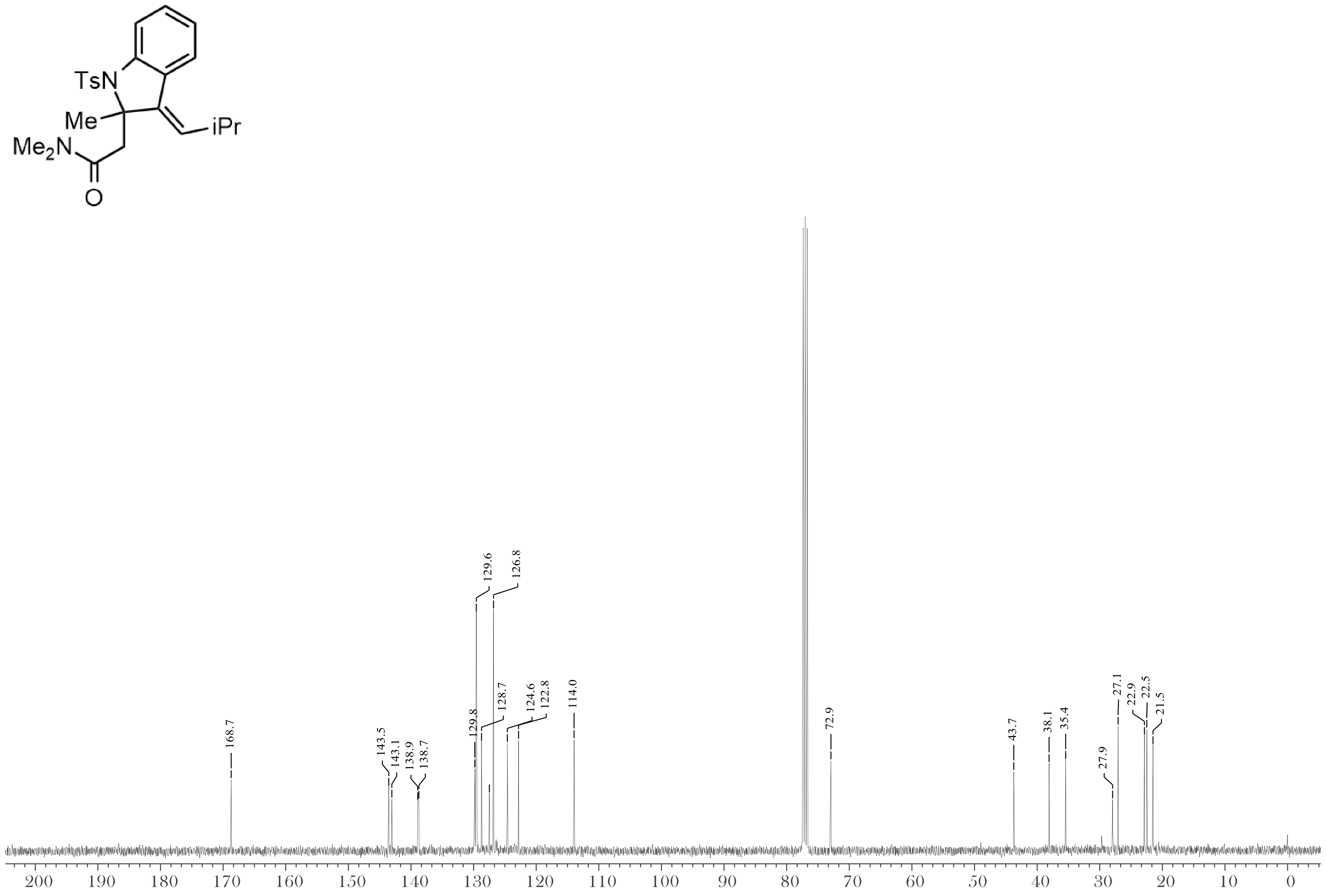

^{13}C NMR Spectrum of **13m** (125 MHz, $CDCl_3$, 25 °C)

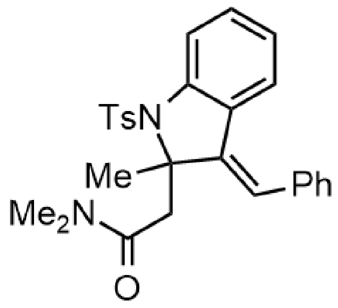

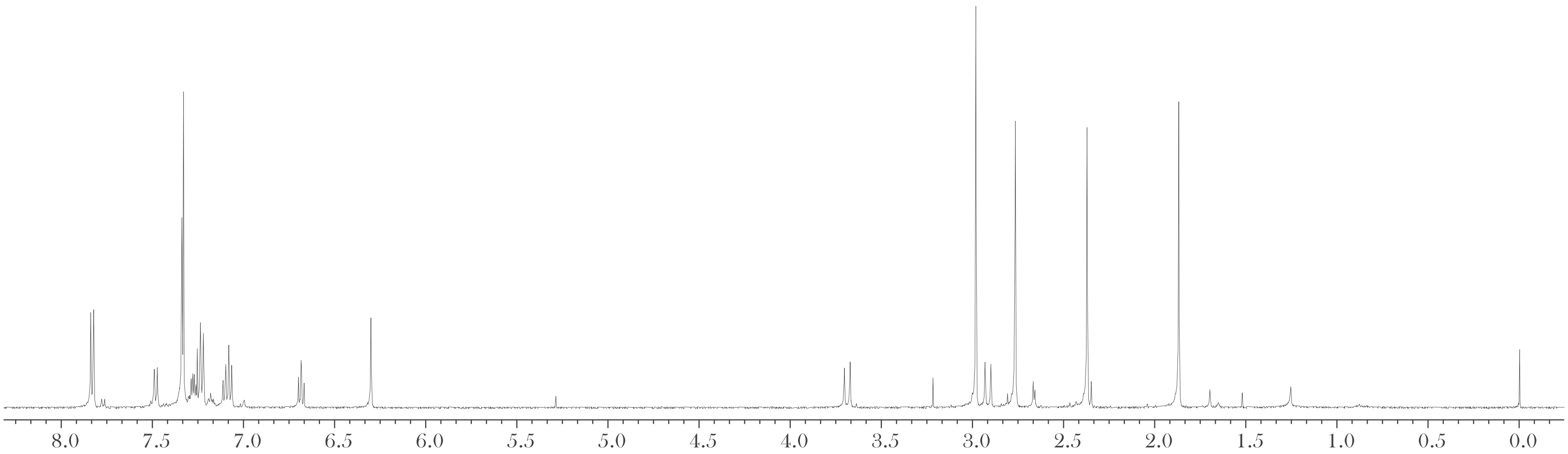

^{1}H NMR Spectrum of **13n** (500 MHz, $CDCl_3$, 25 °C)

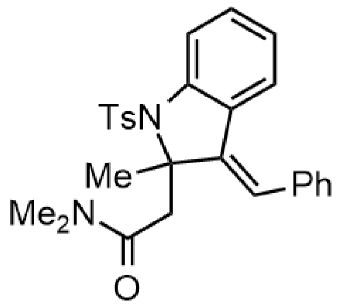

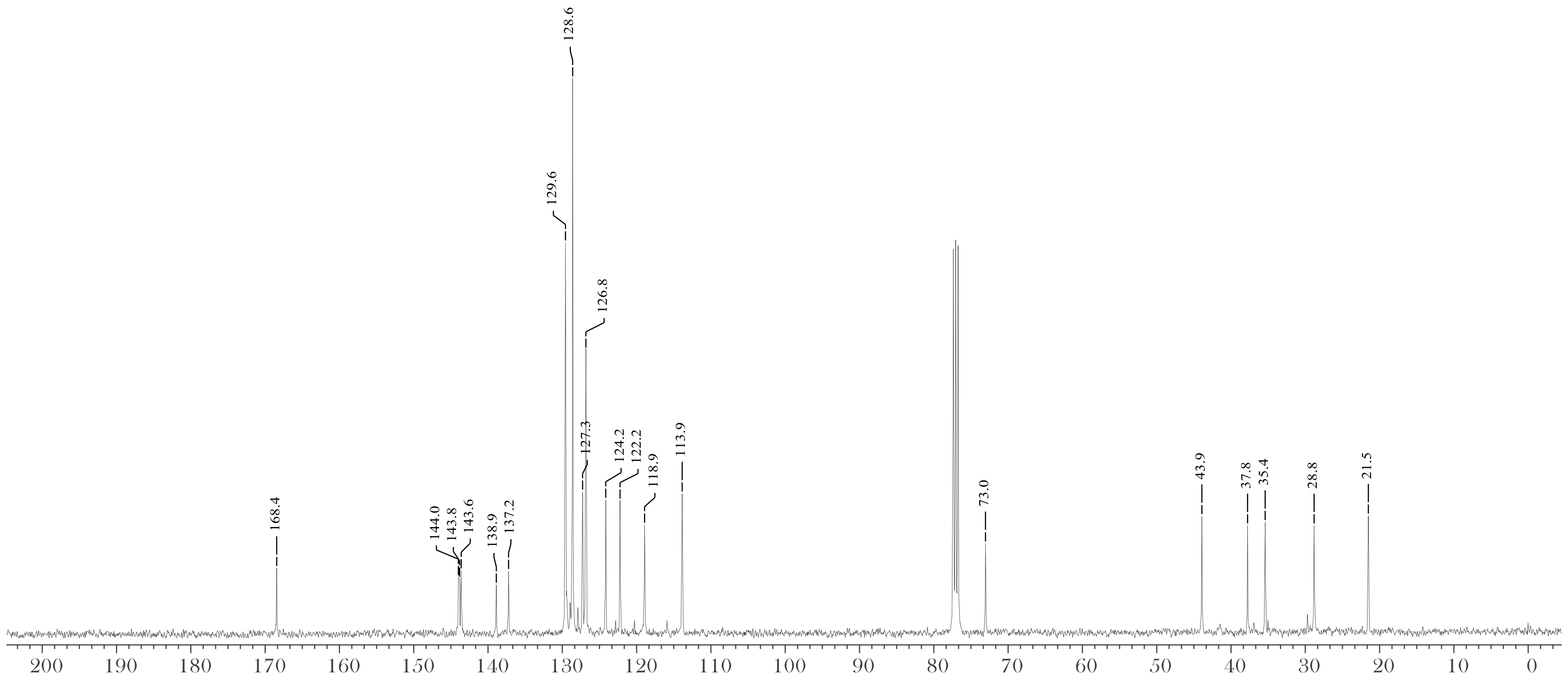

^{13}C NMR Spectrum of **13n** (100 MHz, $CDCl_3$, 25 °C)

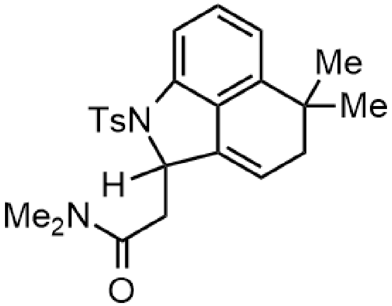

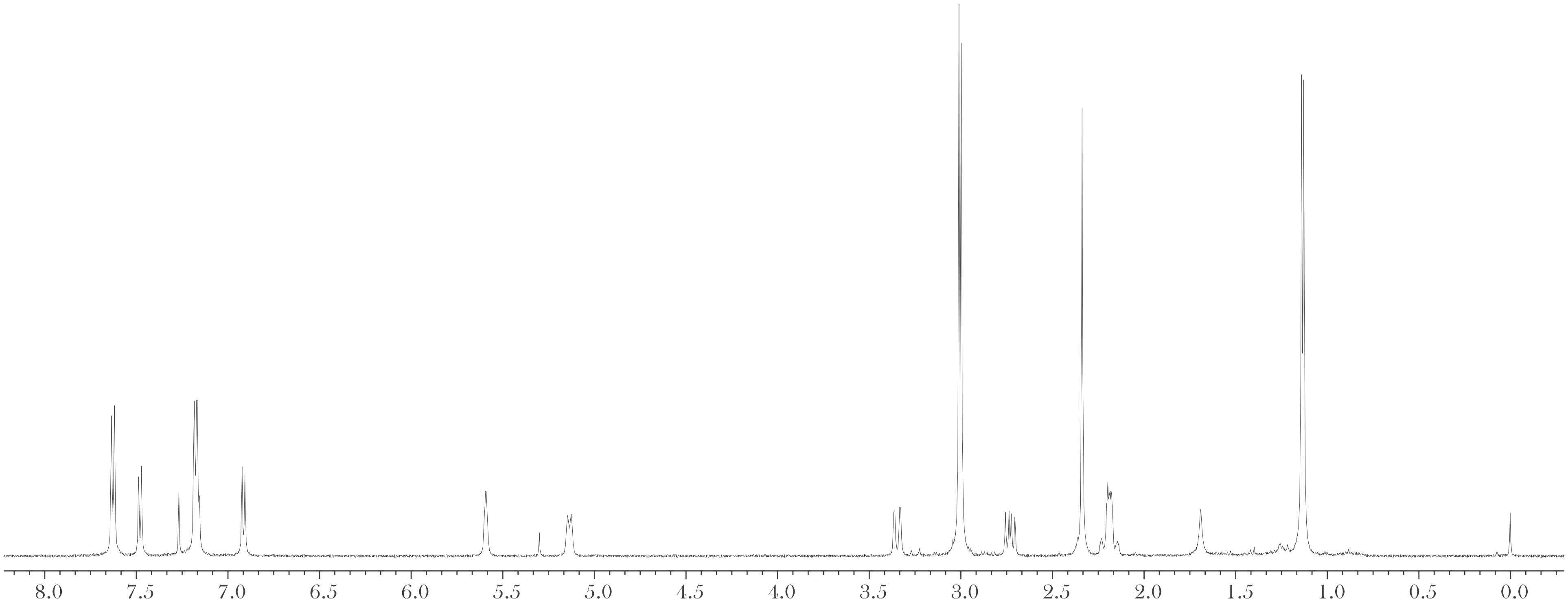

^{1}H NMR Spectrum of **13o** (500 MHz, $CDCl_3$, 25 °C)

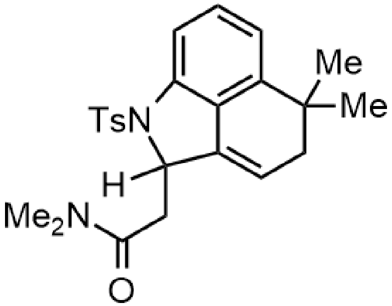

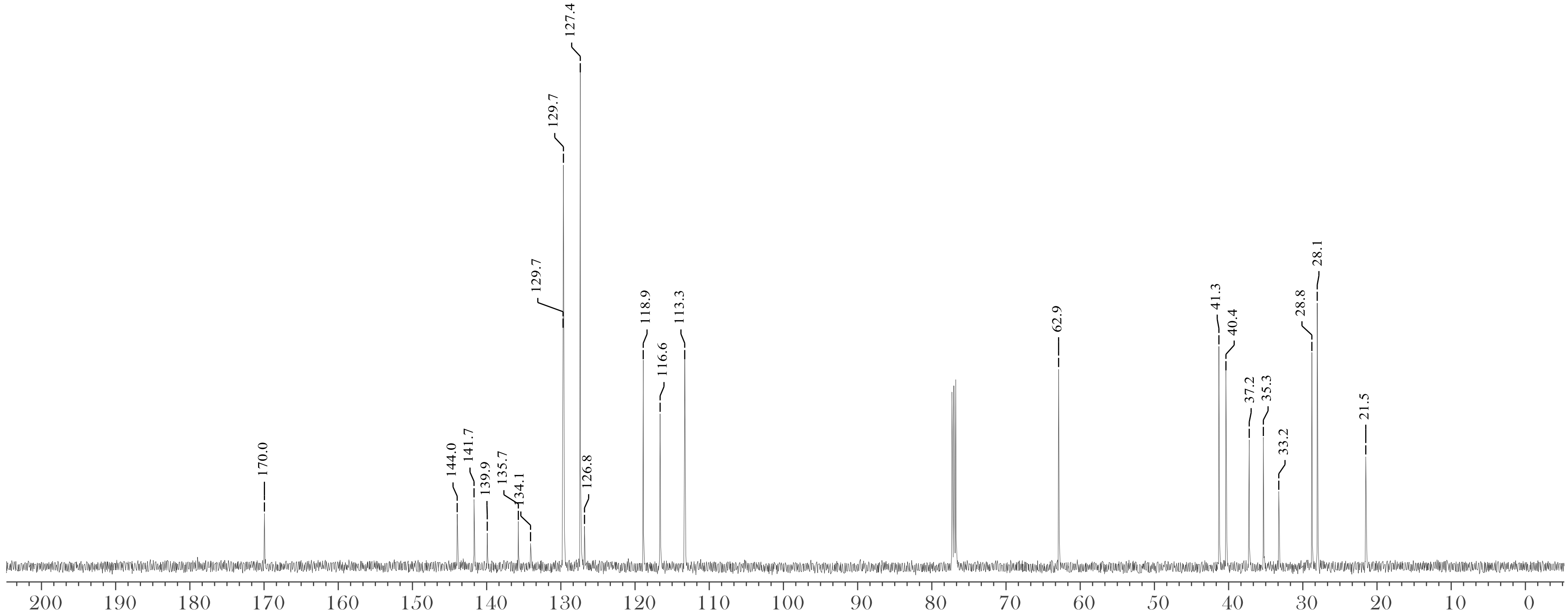

^{13}C NMR Spectrum of **13o** (125 MHz, $CDCl_3$, 25 °C)

J|A|C|S JOURNAL OF THE AMERICAN CHEMICAL SOCIETY

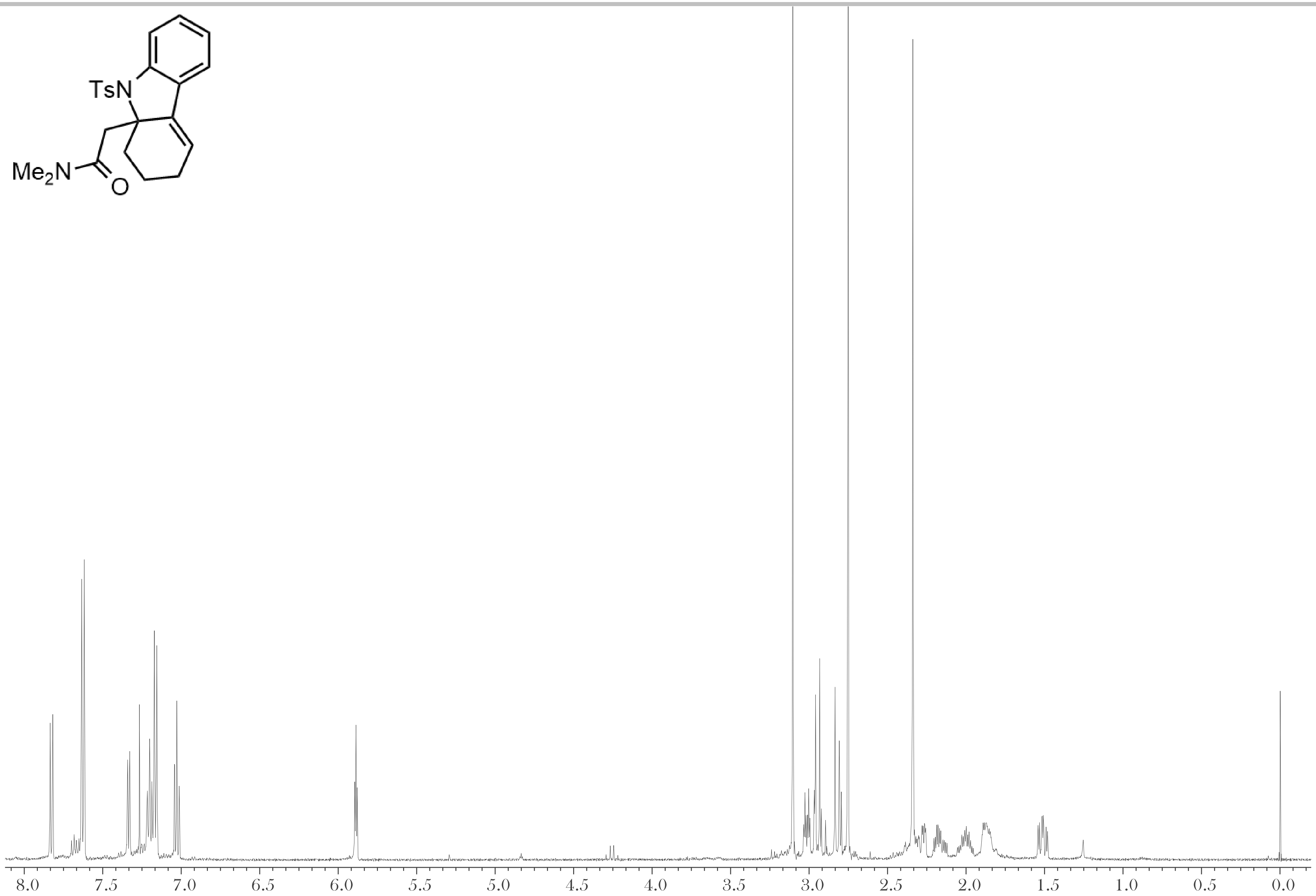

^{1}H NMR Spectrum of **13p** (500 MHz, $CDCl_3$, 25 °C)

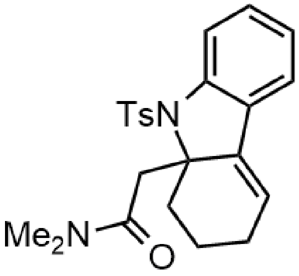

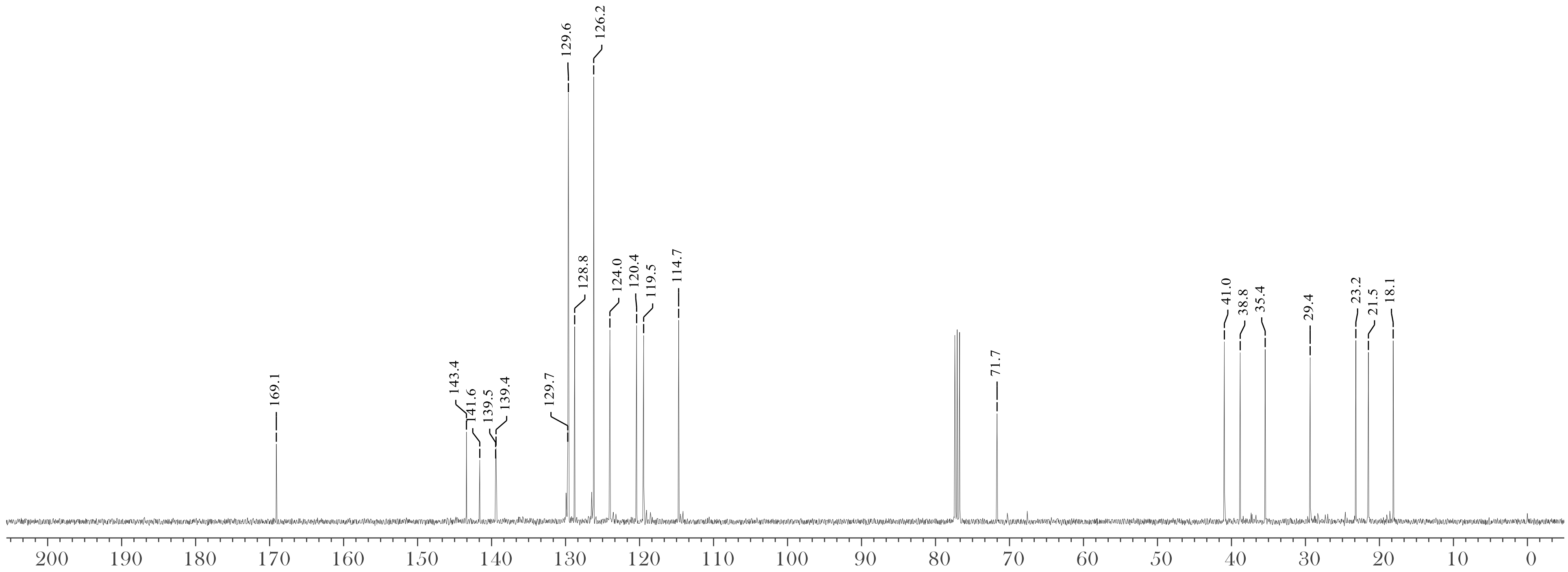

^{13}C NMR Spectrum of **13p** (125 MHz, $CDCl_3$, 25 °C)

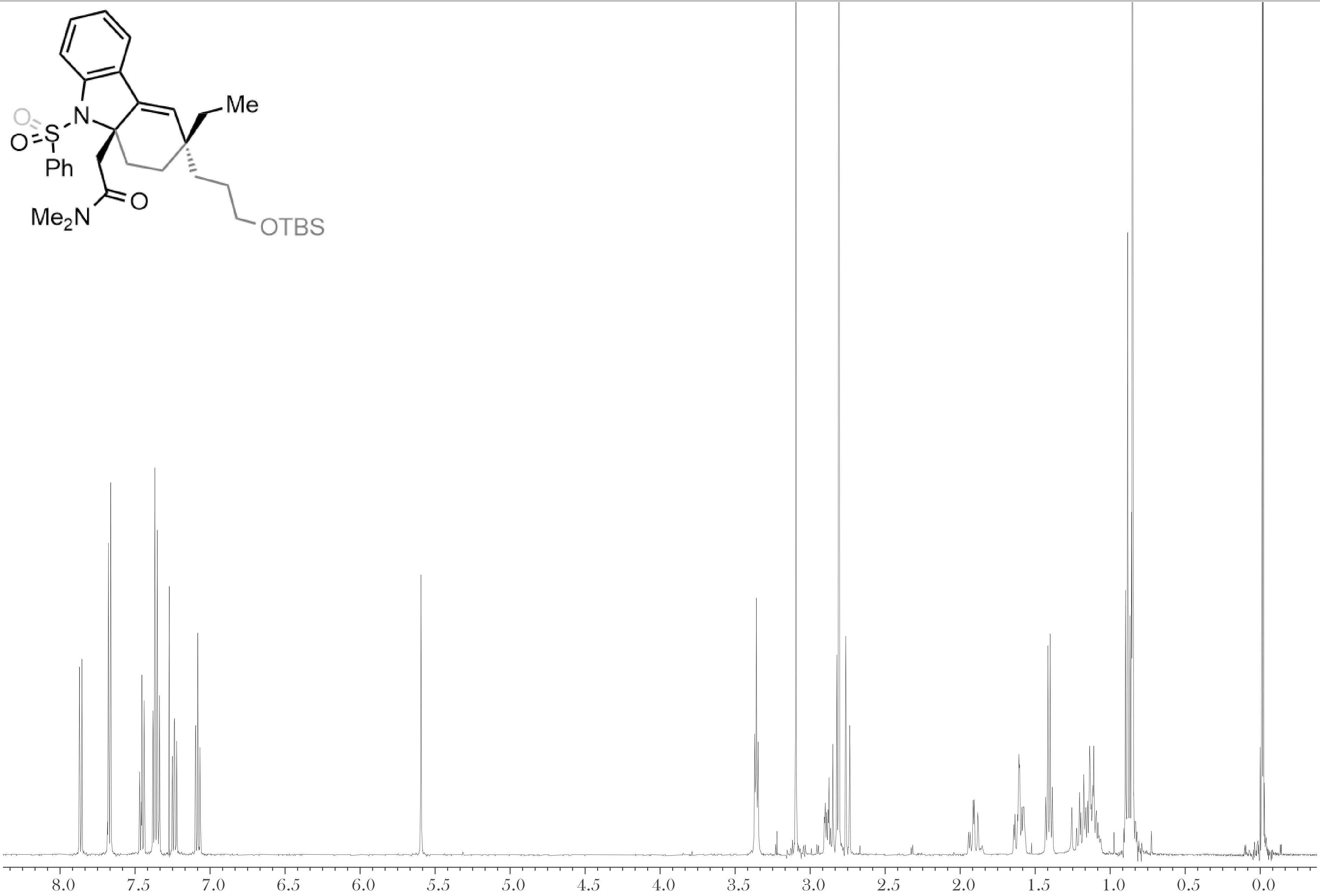

^{1}H NMR Spectrum of **13q** (500 MHz, $CDCl_3$, 25 °C)

JACS JOURNAL OF THE AMERICAN CHEMICAL SOCIETY

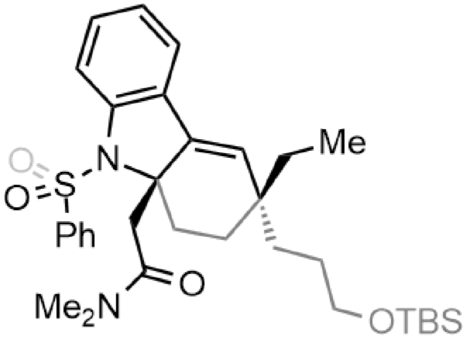

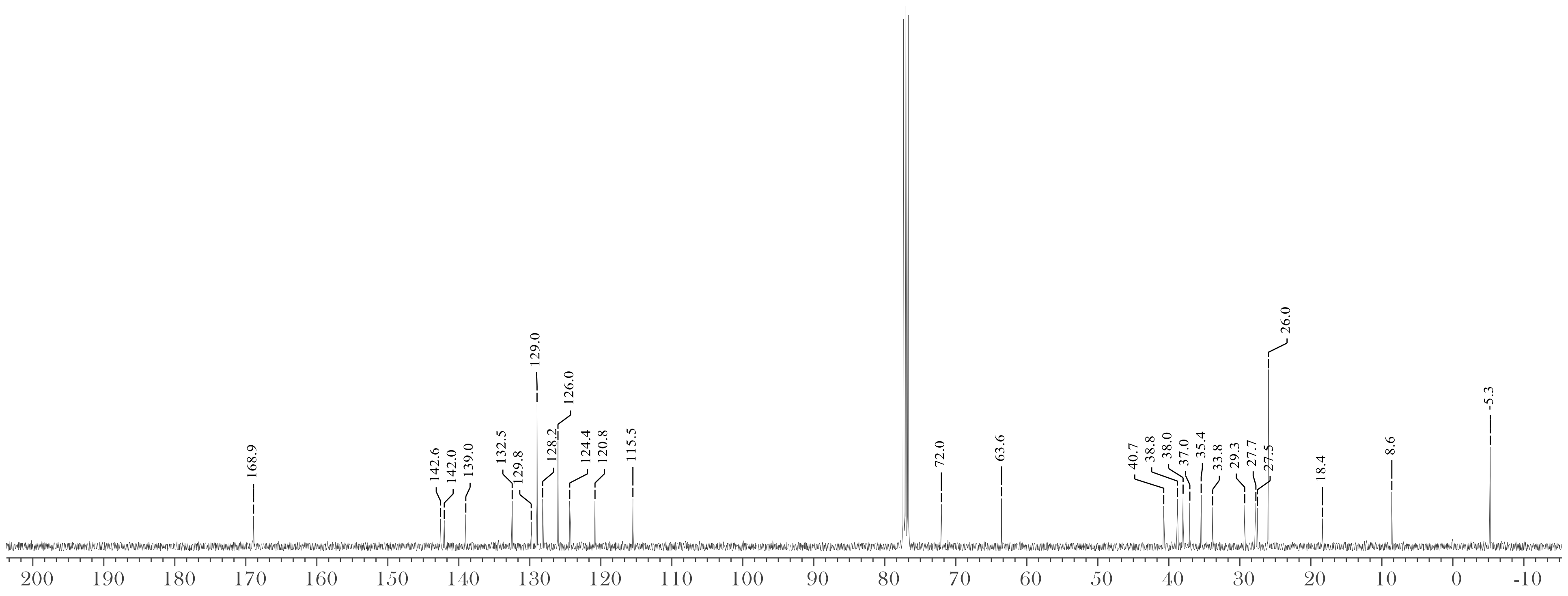

^{13}C NMR Spectrum of **13q** (100 MHz, $CDCl_3$, 25 °C)

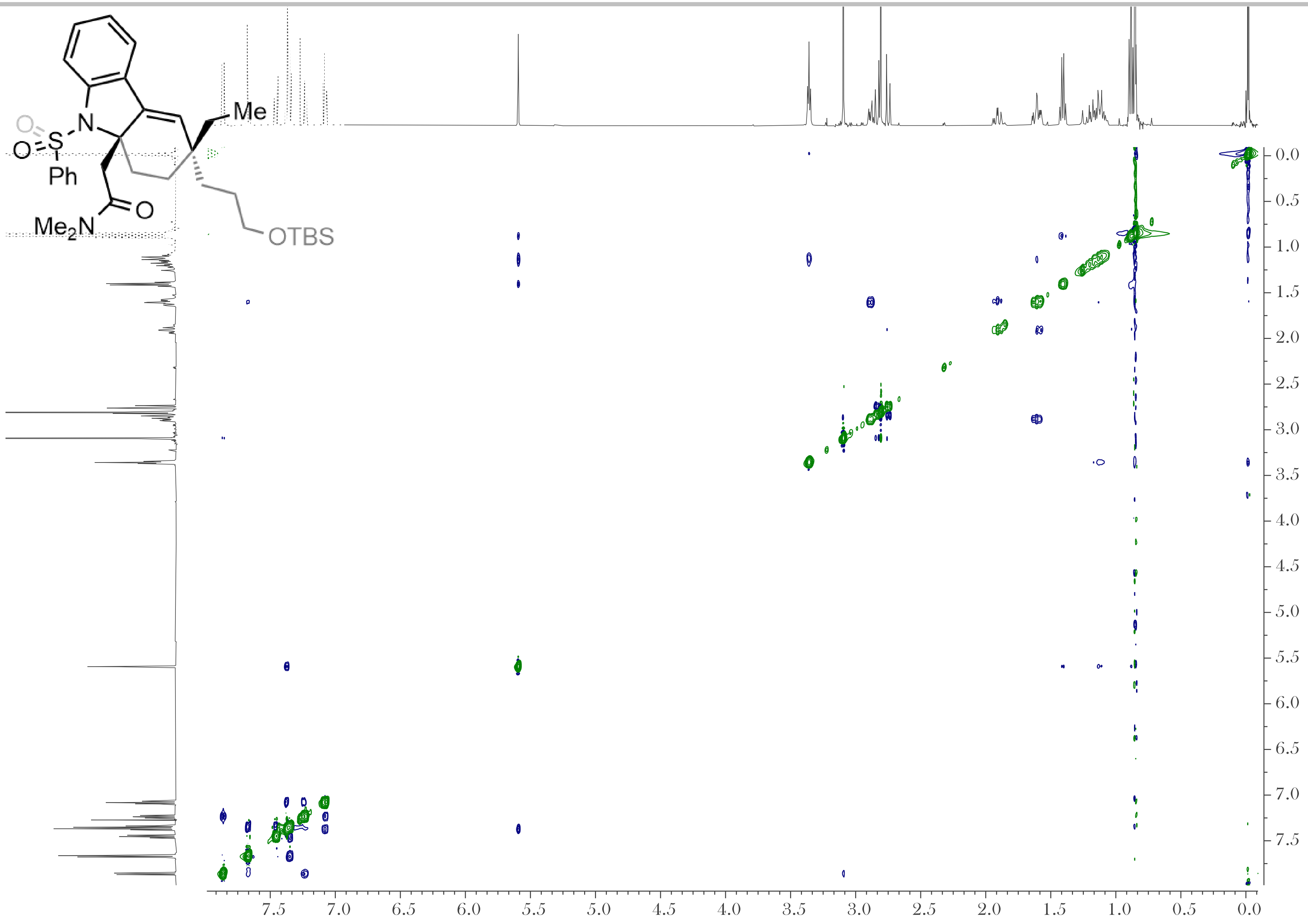

^{1}H–^{1}H NOESY Spectrum of **13q** (500 MHz, $CDCl_3$, 25 °C)

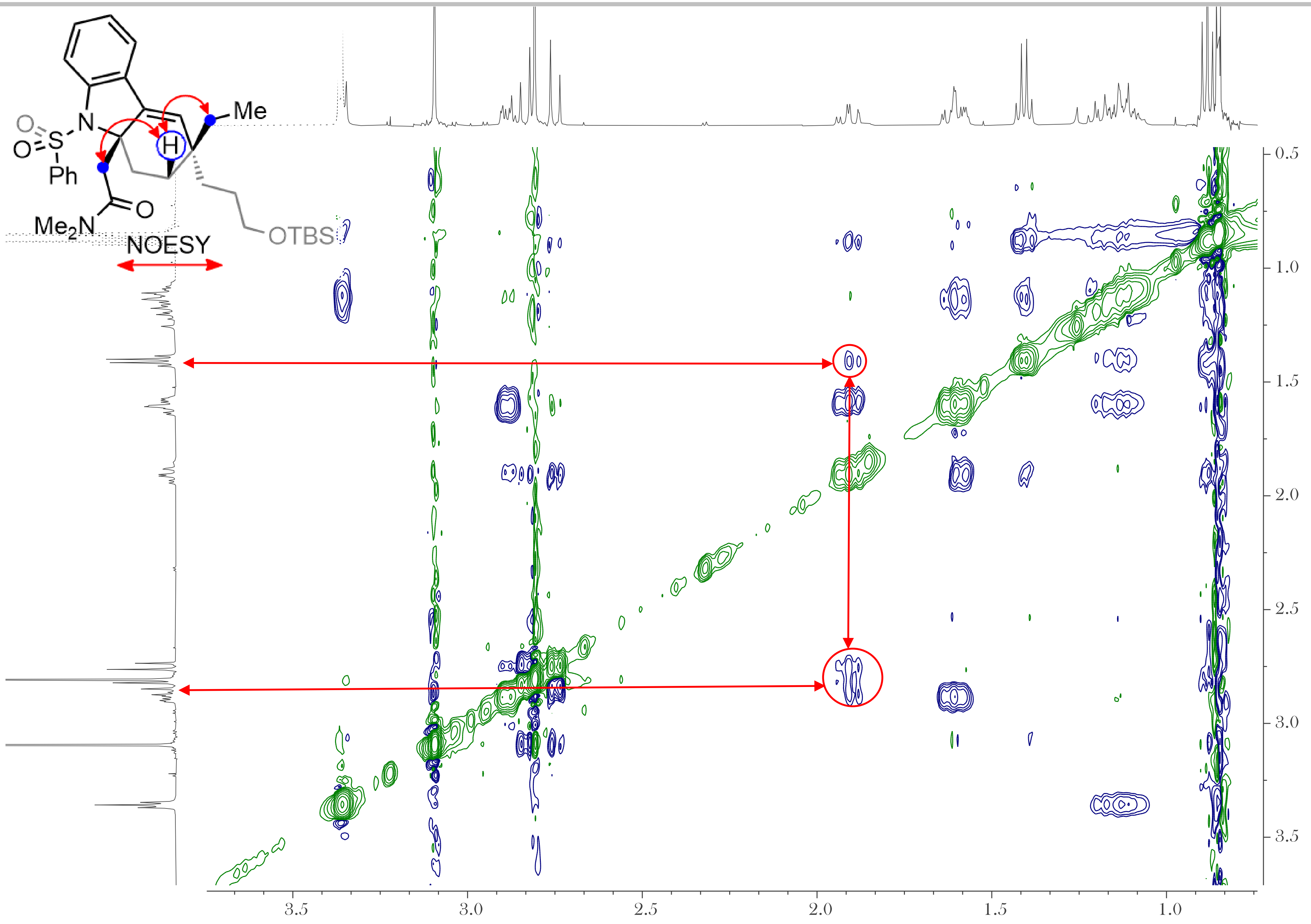

Expanded ^{1}H−^{1}H NOESY Spectrum of **13q** (500 MHz, $CDCl_3$, 25 °C)

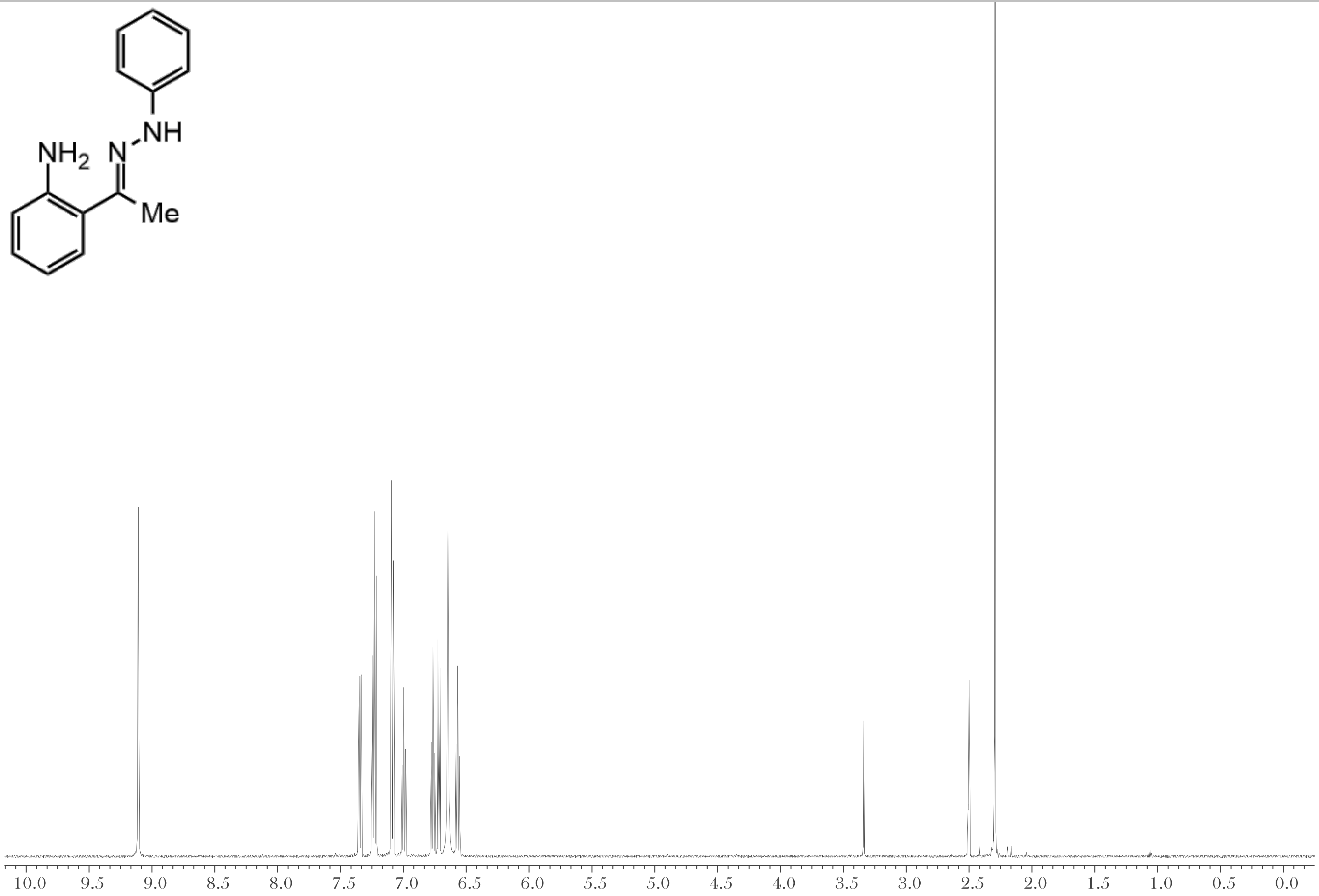

^{1}H NMR Spectrum of **SI-1** (500 MHz, DMSO-d_6, 25 °C)

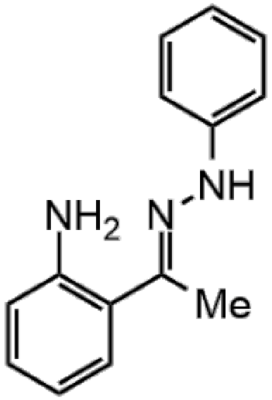

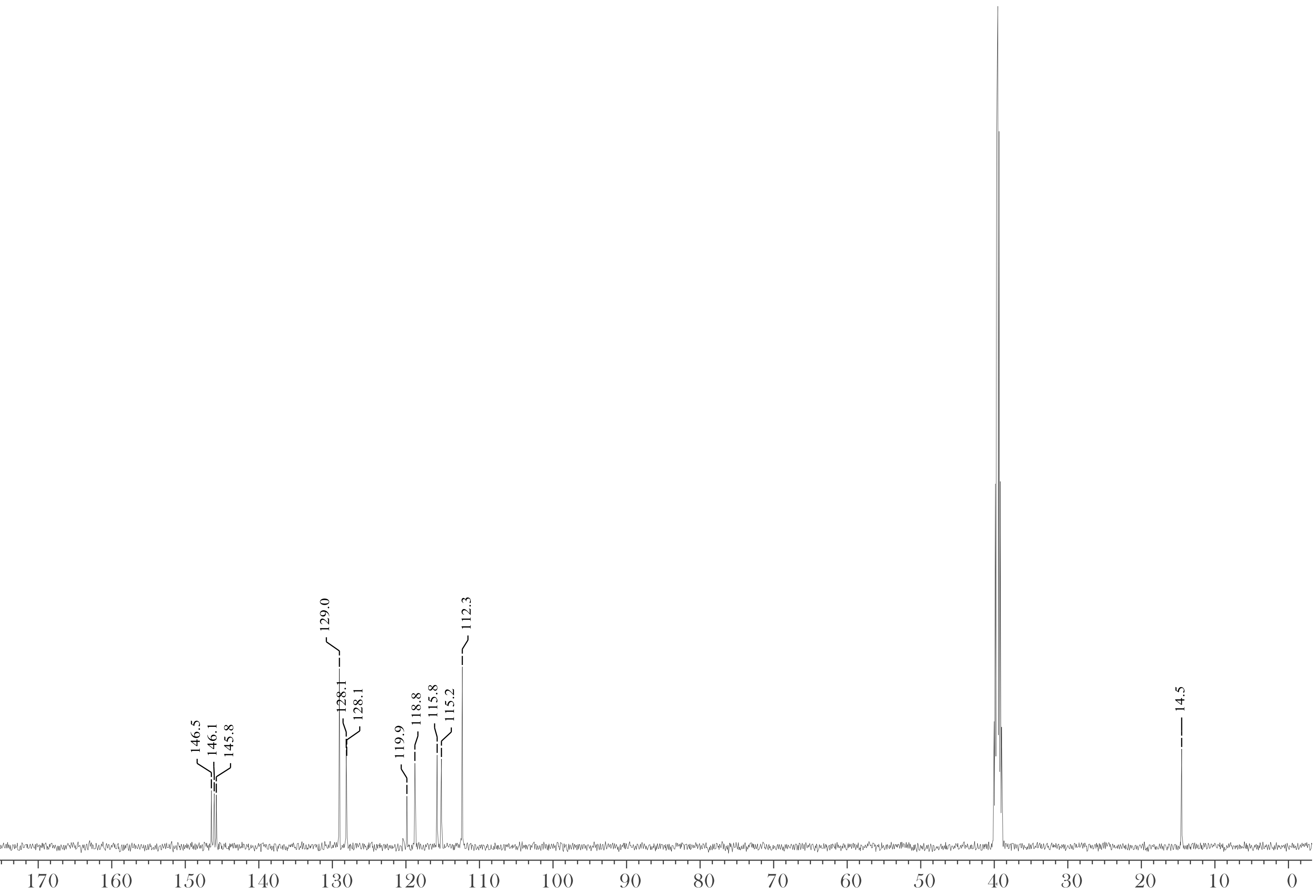

^{13}C NMR Spectrum of **SI-1** (125 MHz, DMSO-d_6, 25 °C)

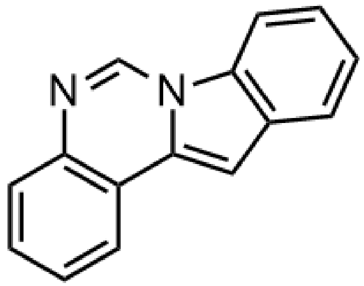

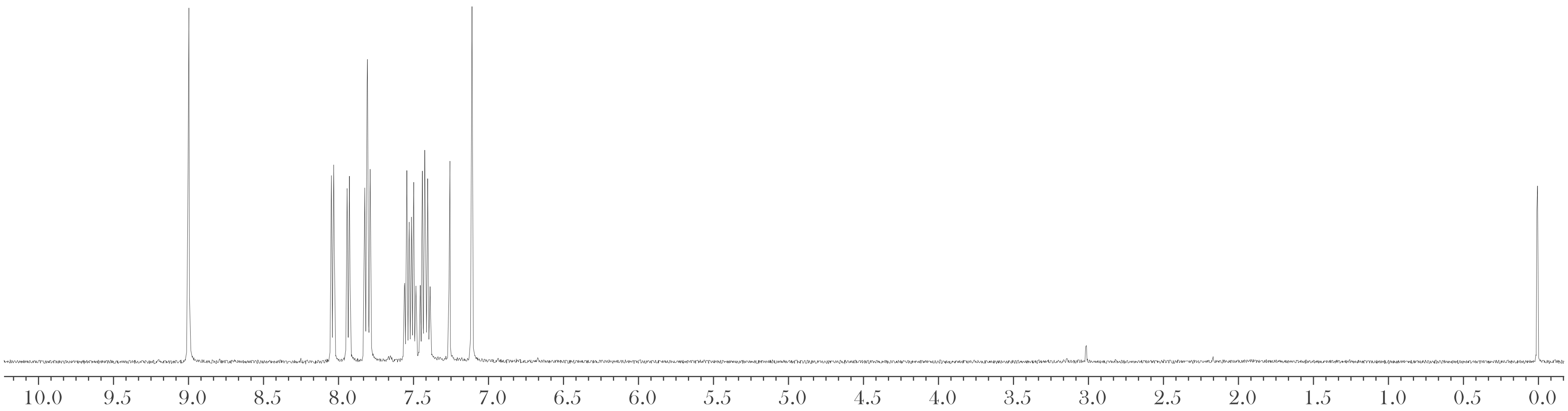

^{1}H NMR Spectrum of **16** (500 MHz, $CDCl_3$, 25 °C)

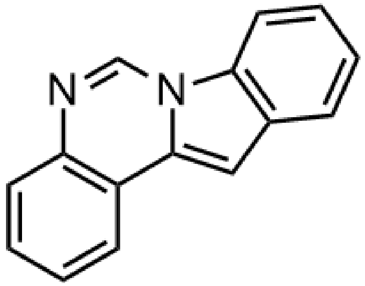

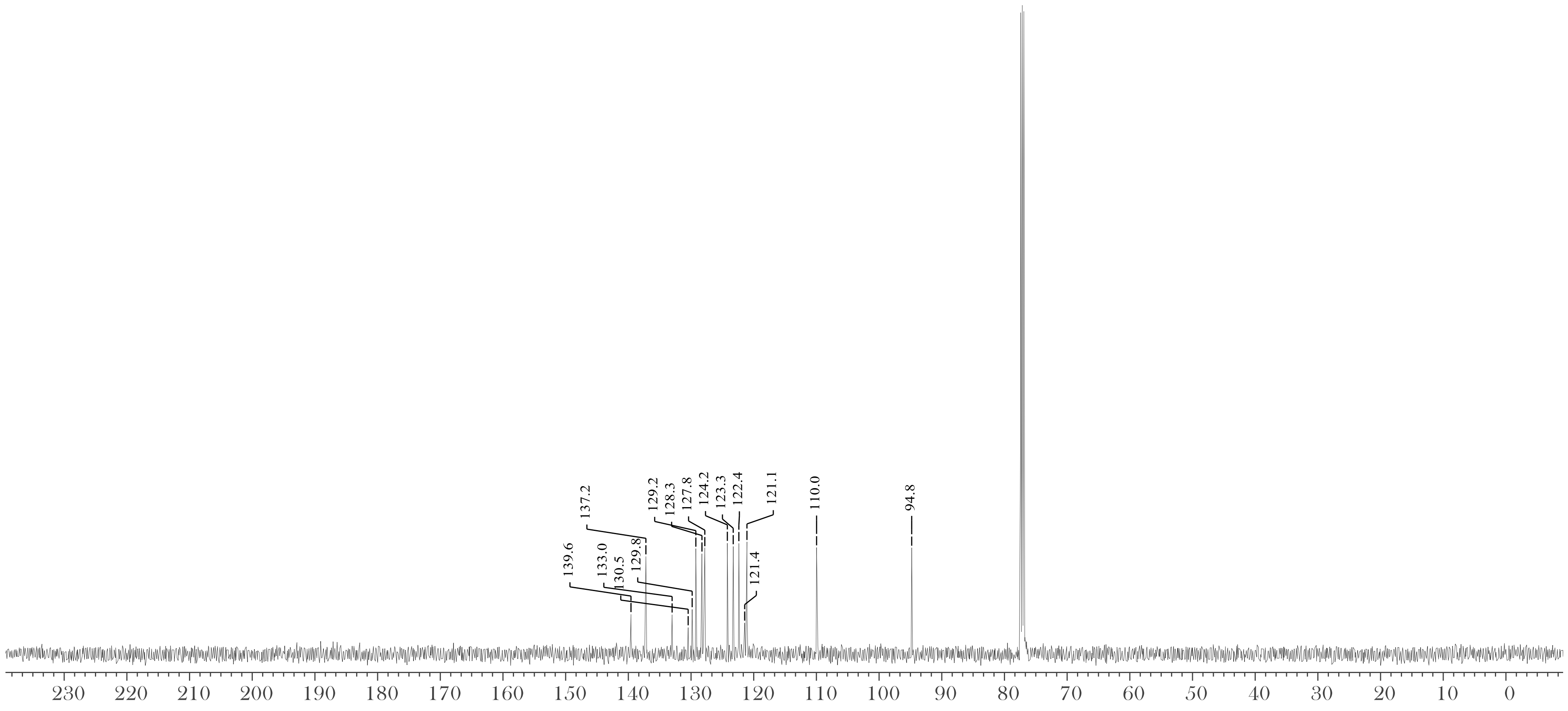

^{13}C NMR Spectrum of **16** (125 MHz, $CDCl_3$, 25 °C)

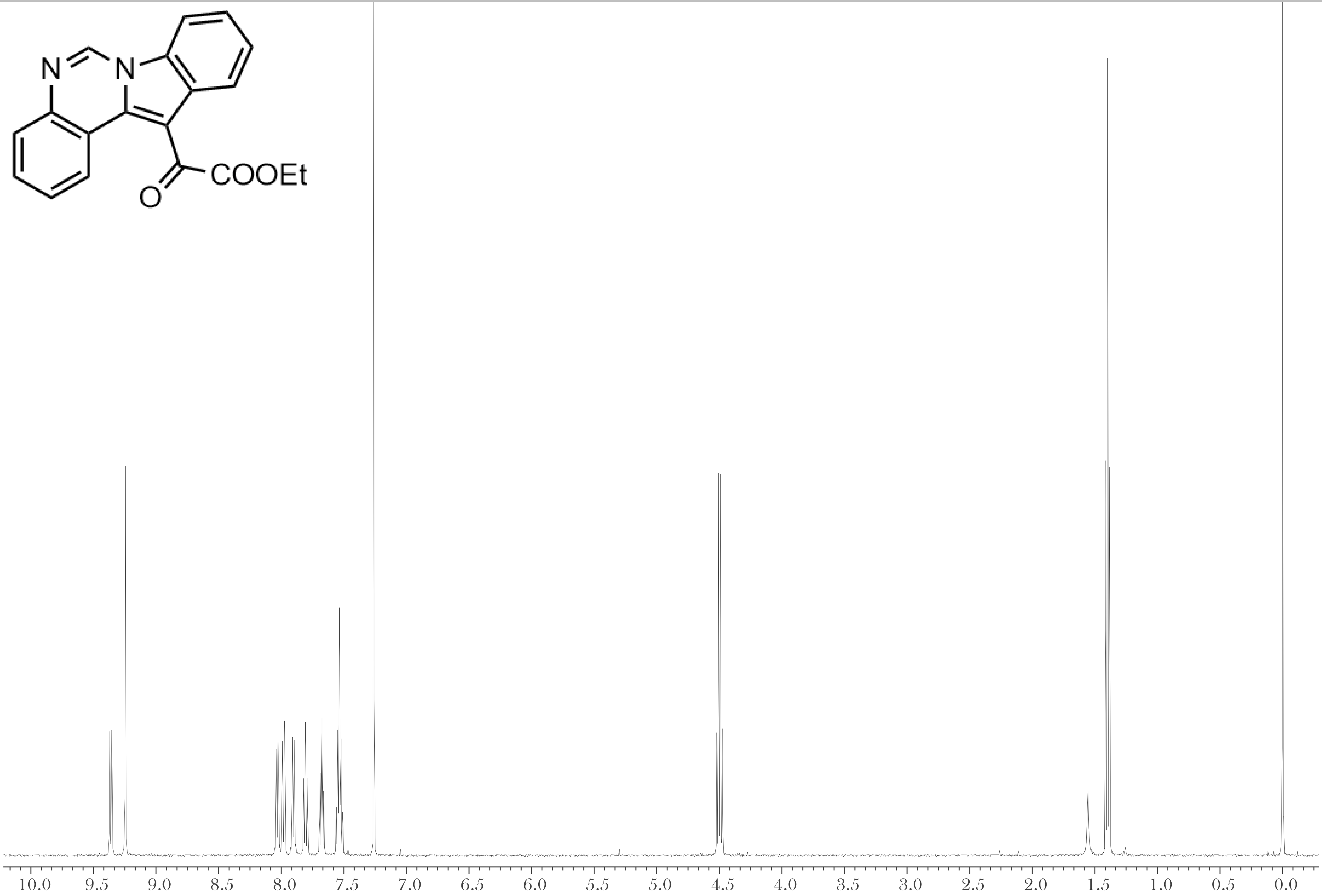

^{1}H NMR Spectrum of **17** (500 MHz, $CDCl_3$, 25 °C)

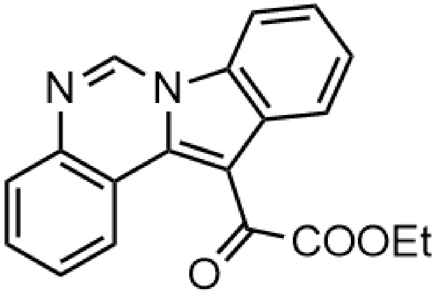

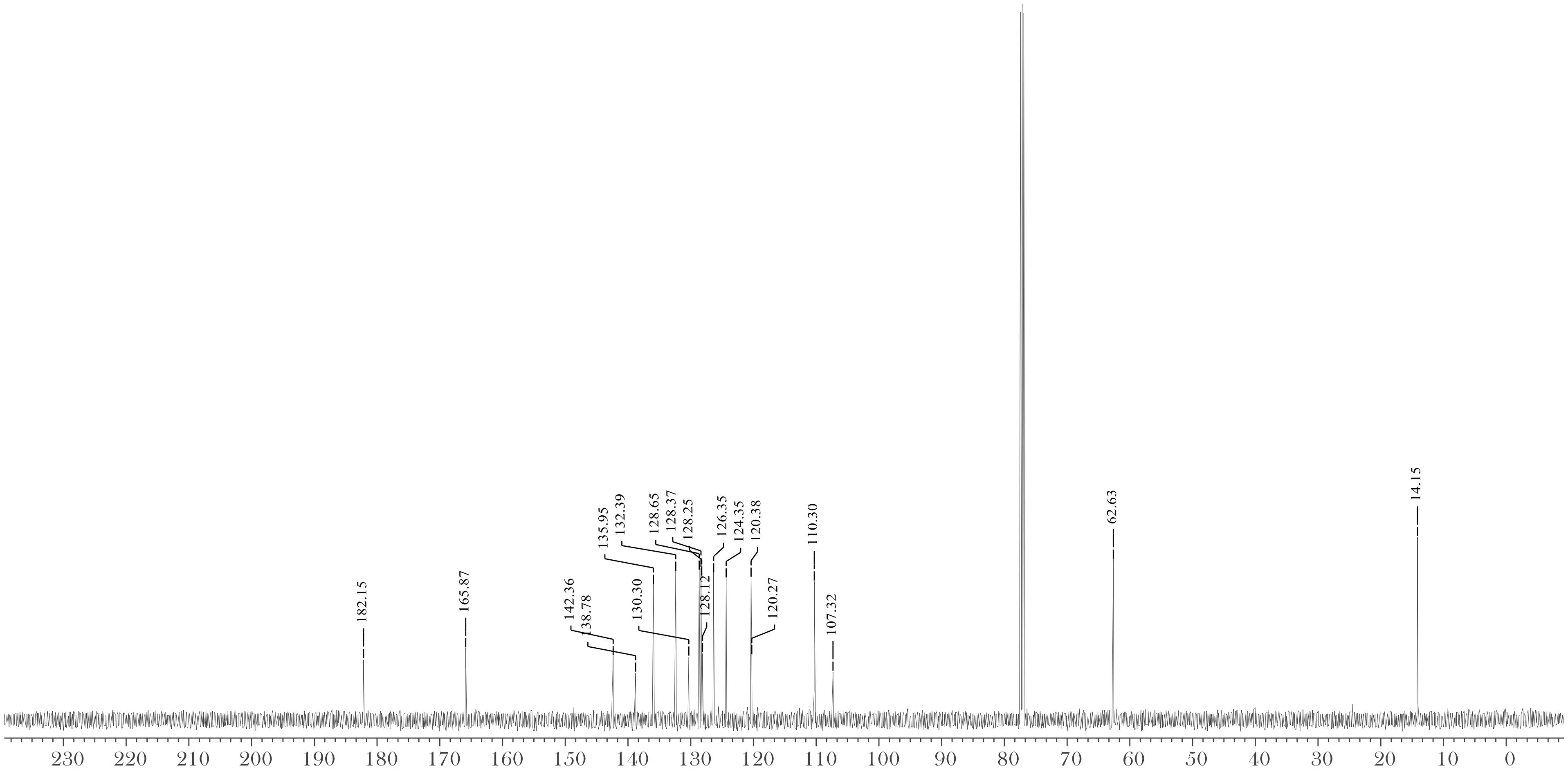

^{13}C NMR Spectrum of **17** (125 MHz, $CDCl_3$, 25 °C)

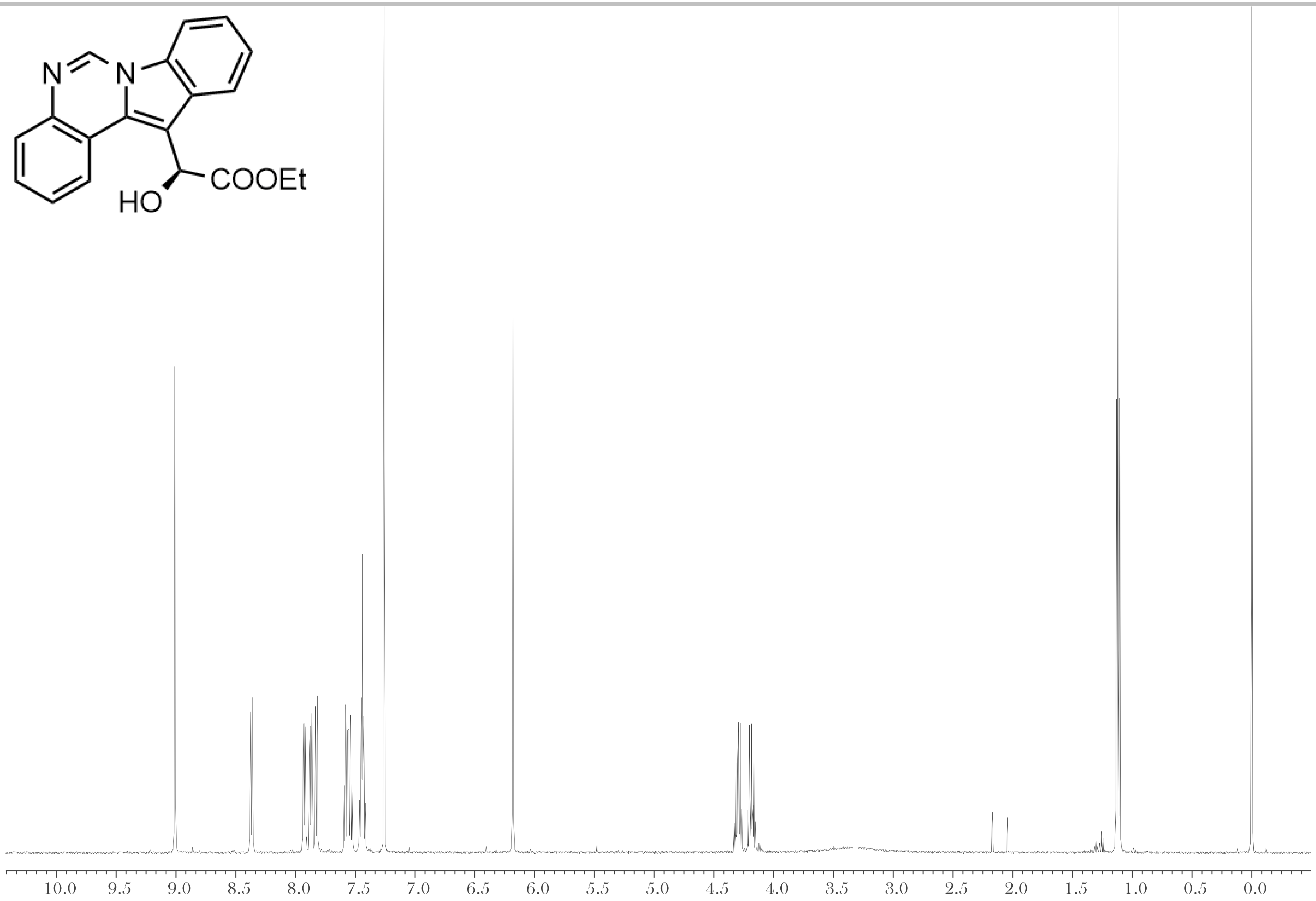

^{1}H NMR Spectrum of **18** (500 MHz, $CDCl_3$, 25 °C)

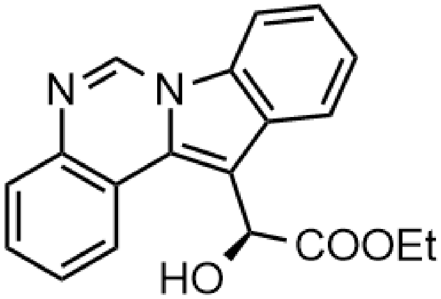

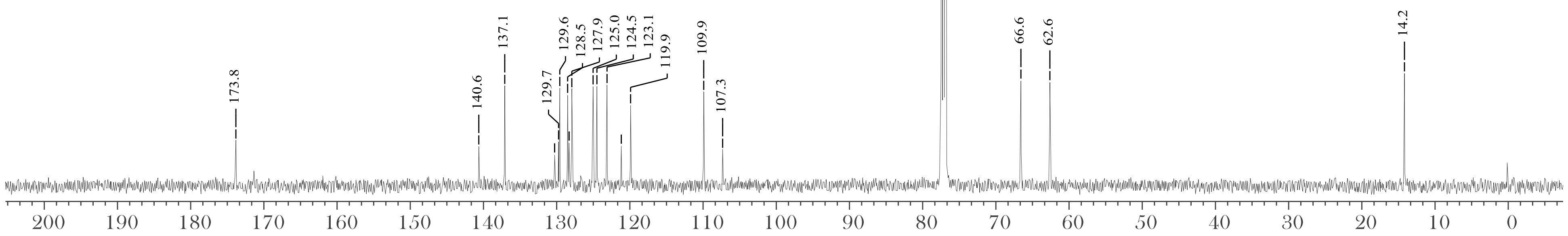

^{13}C NMR Spectrum of **18** (125 MHz, $CDCl_3$, 25 °C)

JACS JOURNAL OF THE AMERICAN CHEMICAL SOCIETY

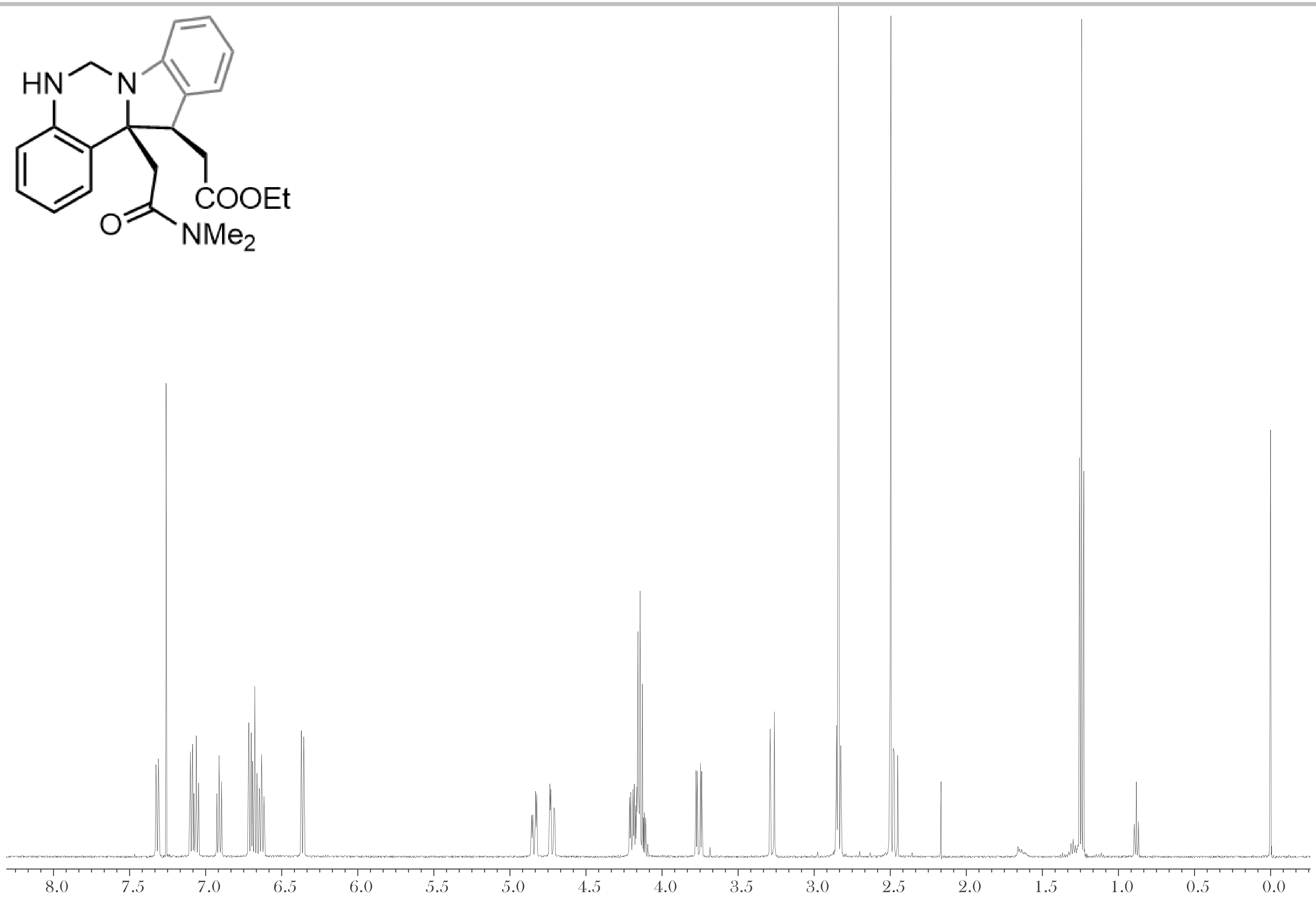

^{1}H NMR Spectrum of **19** (500 MHz, $CDCl_3$, 25 °C)

J|A|C|S JOURNAL OF THE AMERICAN CHEMICAL SOCIETY

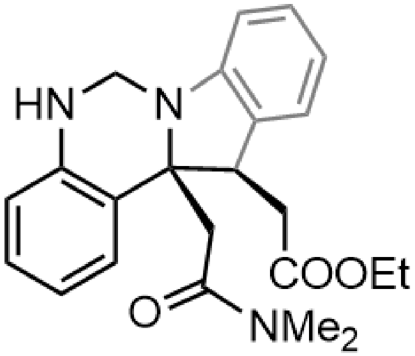

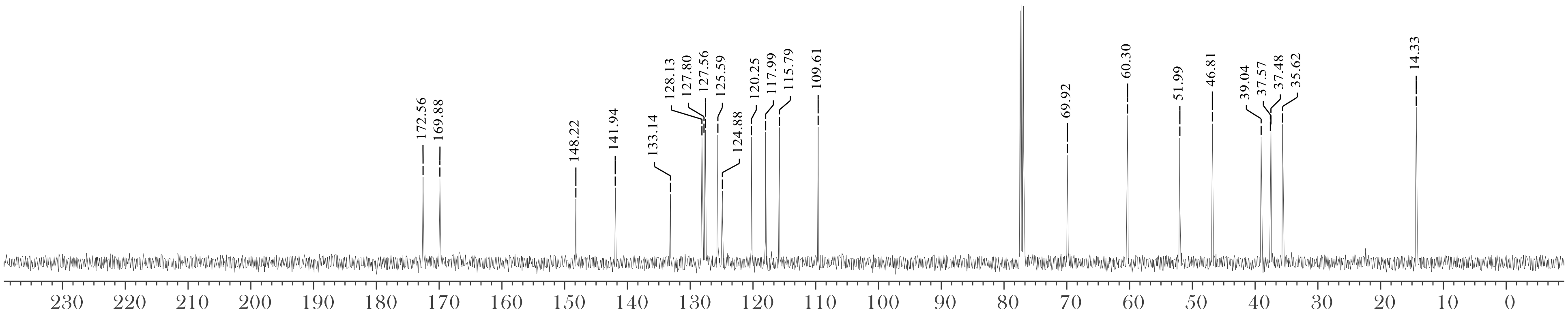

^{13}C NMR Spectrum of **19** (125 MHz, $CDCl_3$, 25 °C)

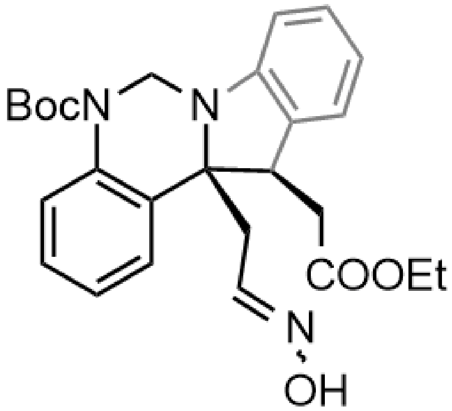

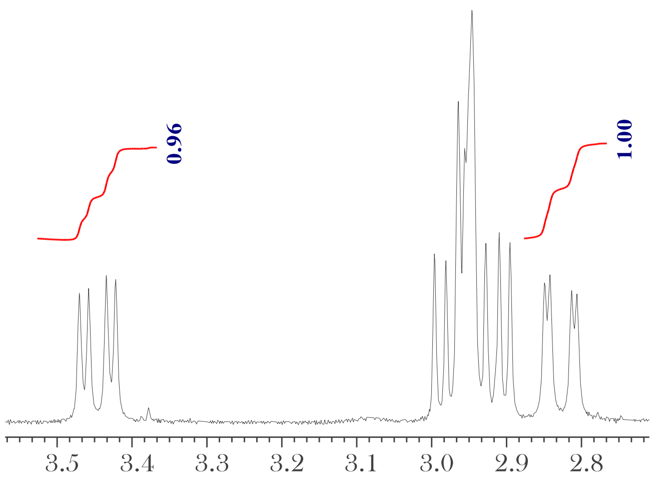

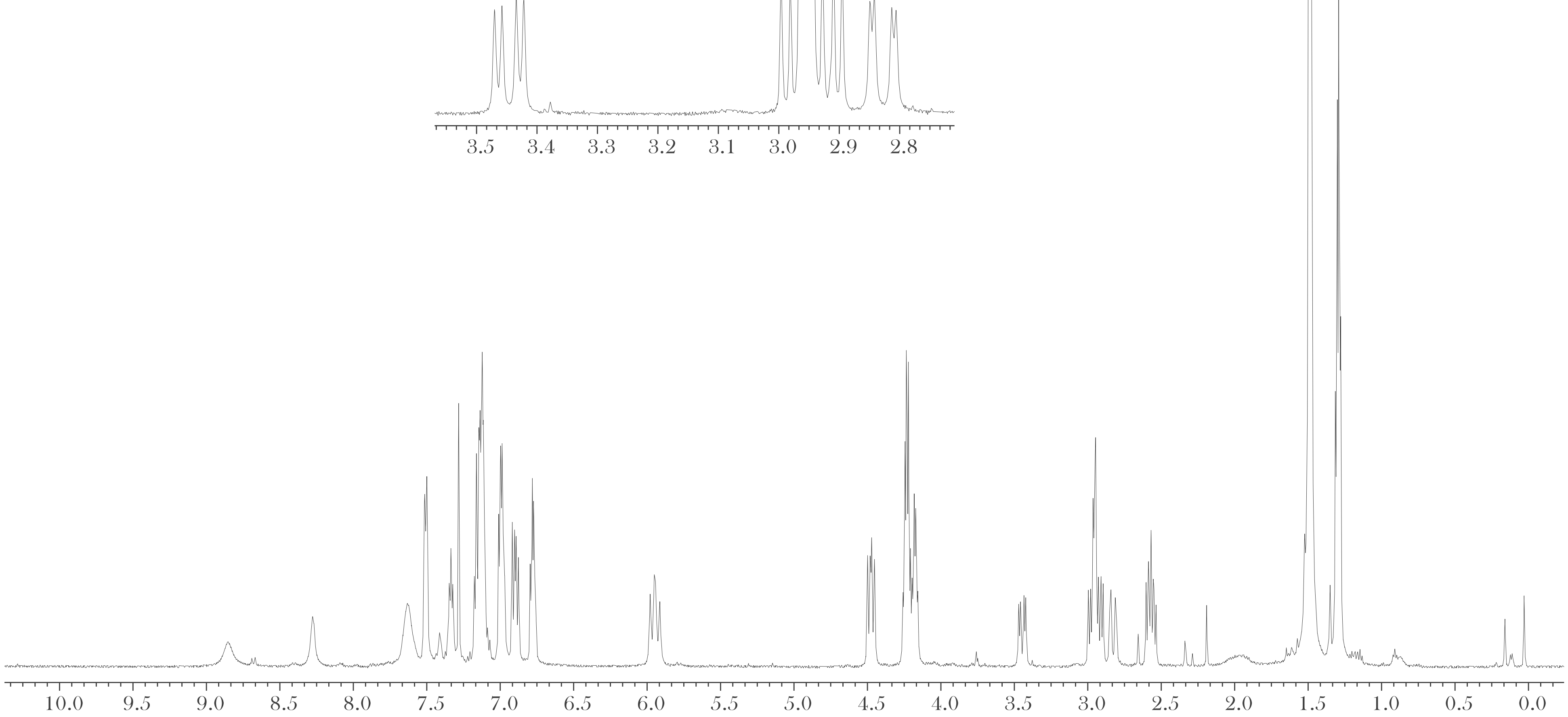

^{1}H NMR Spectrum of **20** (*cis*/*trans* = 1:1; 500 MHz, $CDCl_3$, 25 °C)

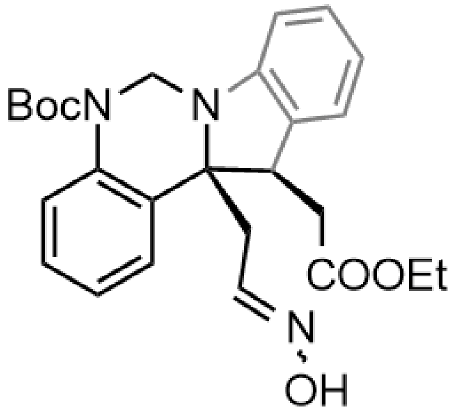

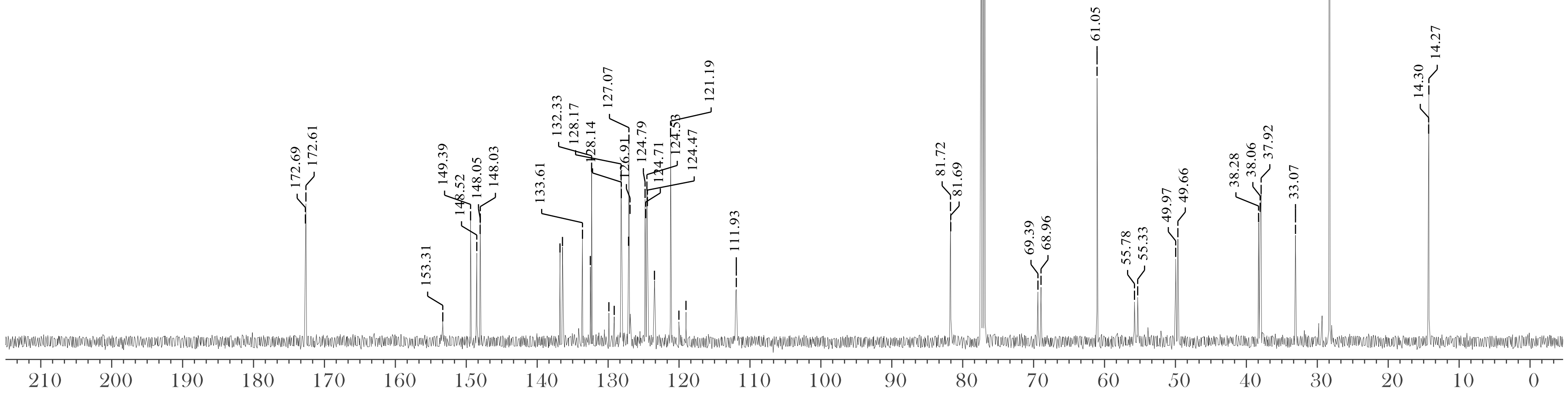

^{13}C NMR Spectrum of **20** (*cis*/*trans* = 1:1; 125 MHz, $CDCl_3$, 25 °C)

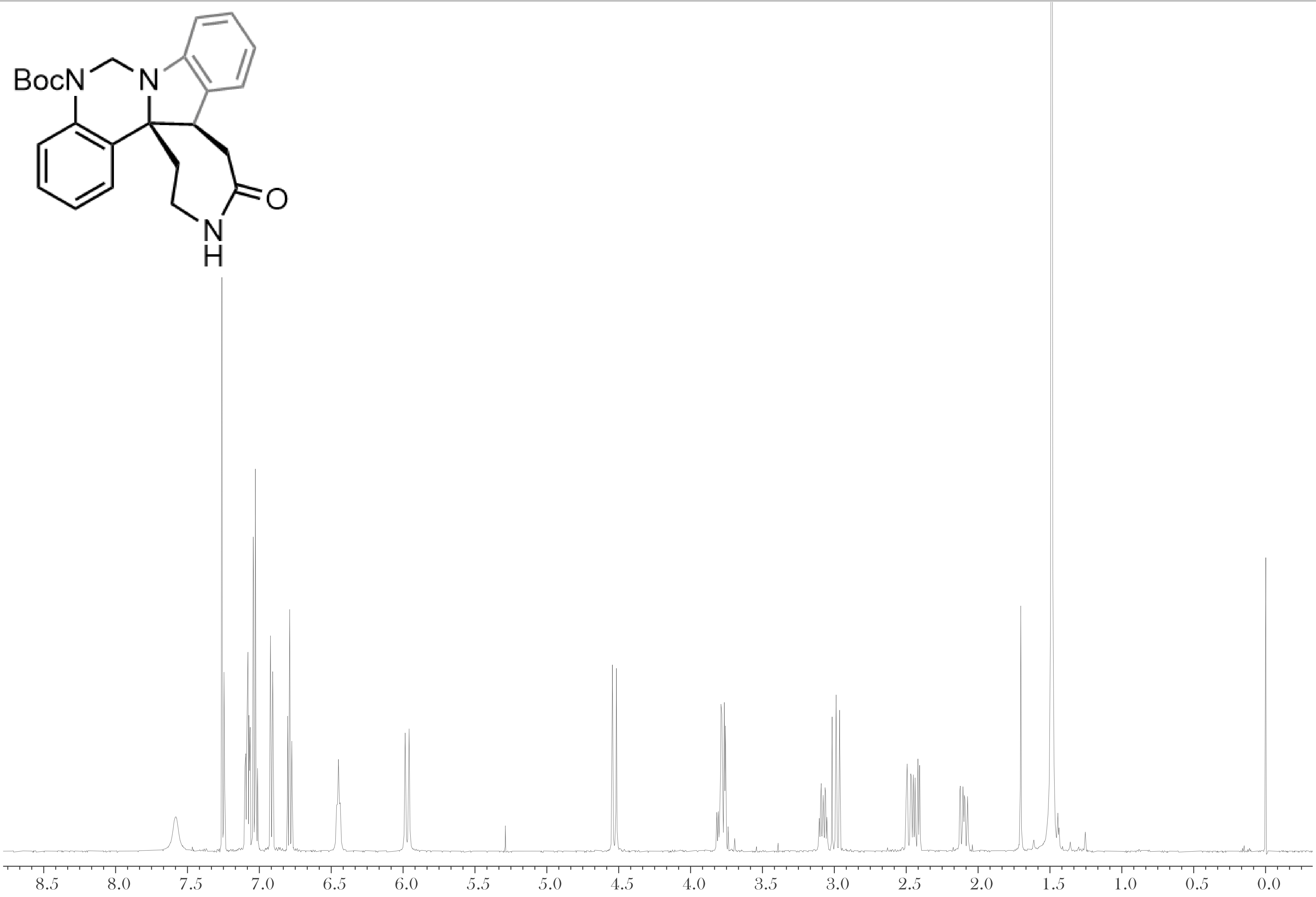

^{1}H NMR Spectrum of **21** (500 MHz, $CDCl_3$, 25 °C)

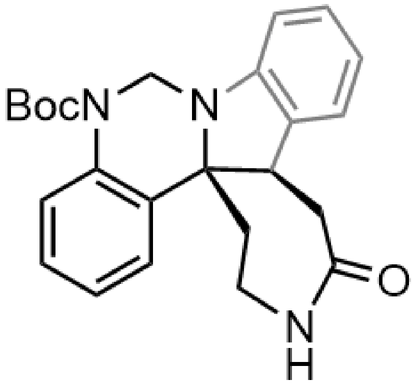

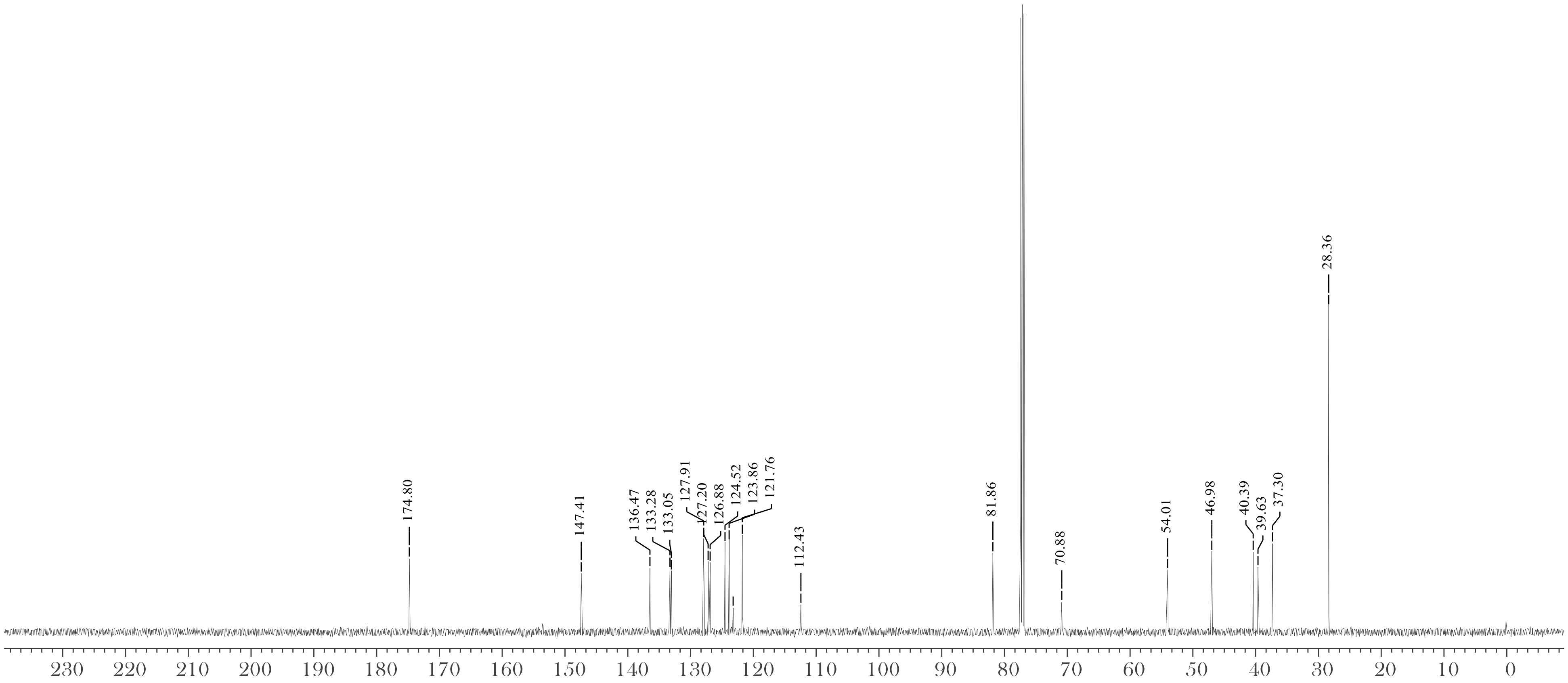

^{13}C NMR Spectrum of **21** (125 MHz, $CDCl_3$, 25 °C)

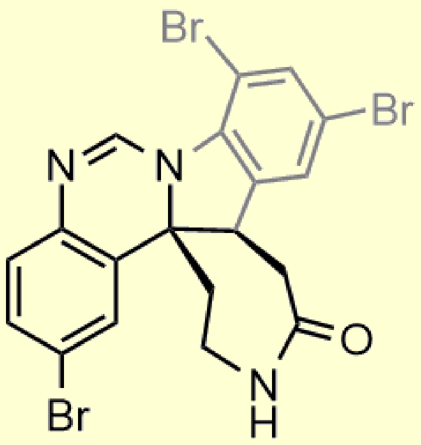

(+)-**1**

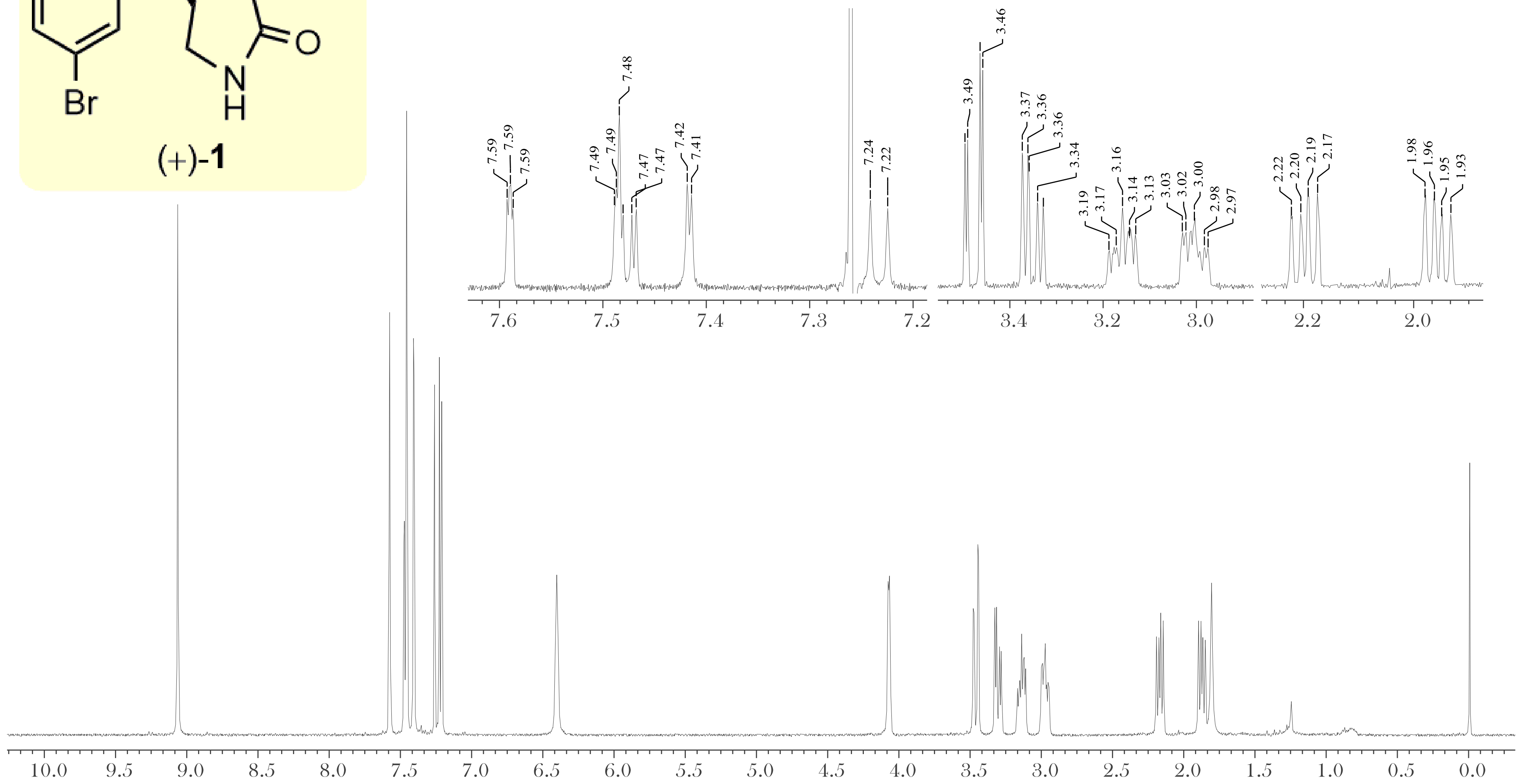

^{1}H NMR Spectrum of (+)-hinckdentine A (500 MHz, $CDCl_3$, 25 °C)

J|A|C|S
JOURNAL OF THE AMERICAN CHEMICAL SOCIETY

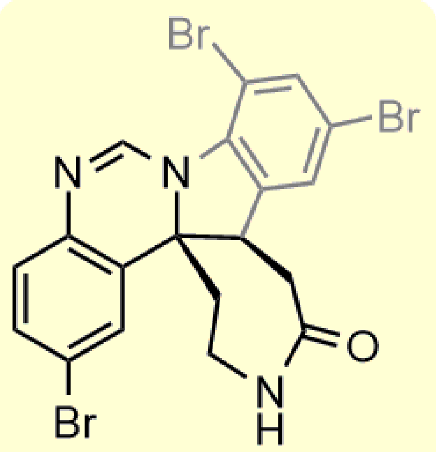

(+)-**1**

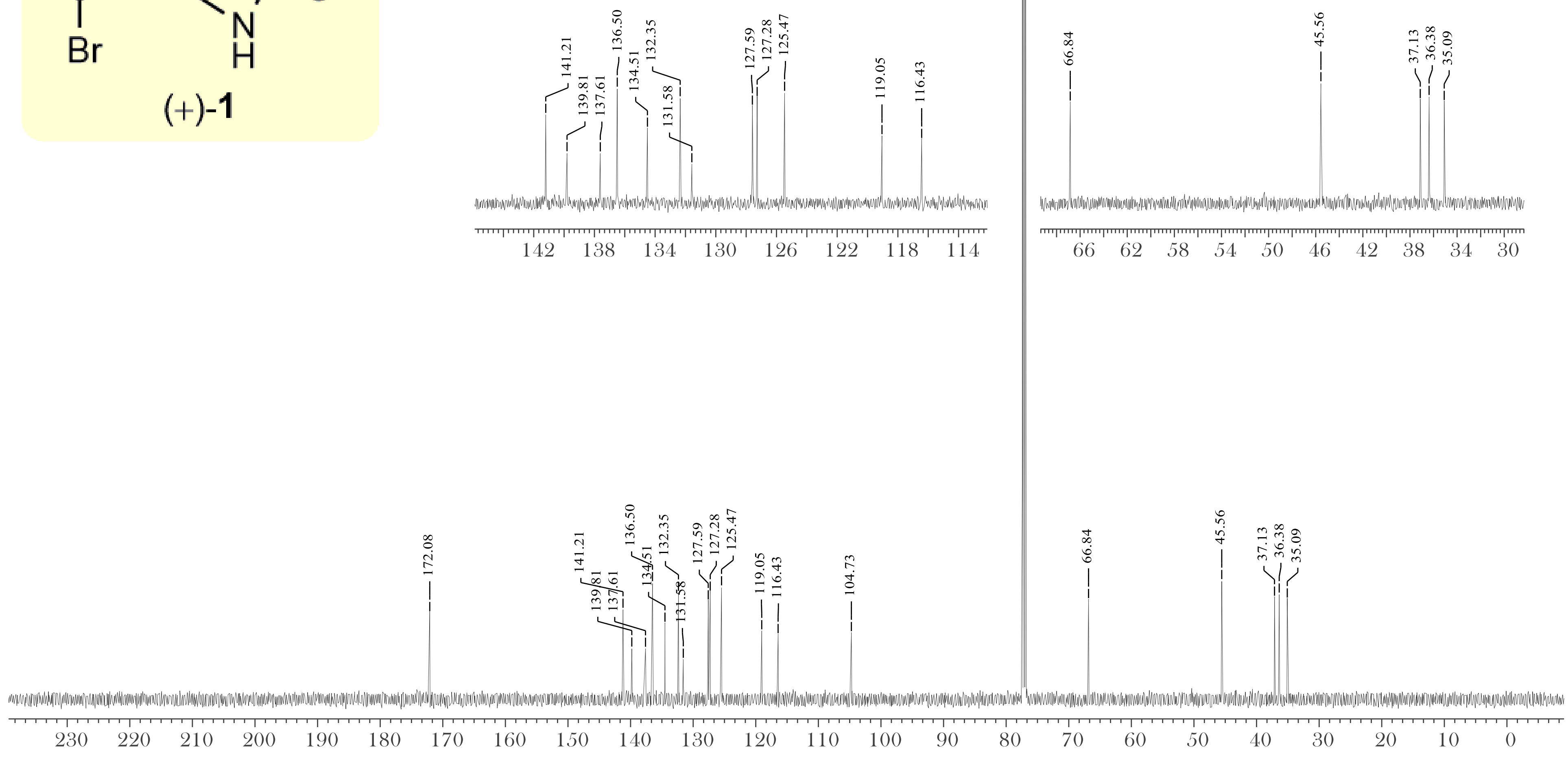

^{13}C NMR Spectrum of (+)-hinckdentine A (125 MHz, $CDCl_3$, 25 °C)

JACS JOURNAL OF THE AMERICAN CHEMICAL SOCIETY

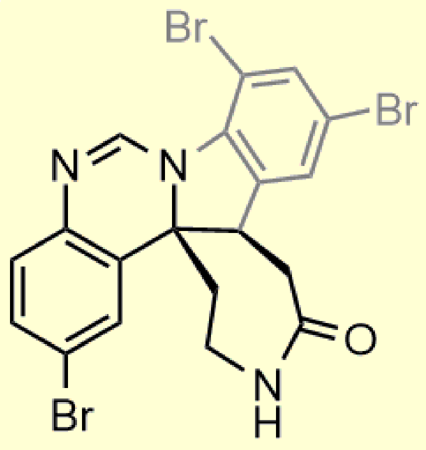

(+)-**1**

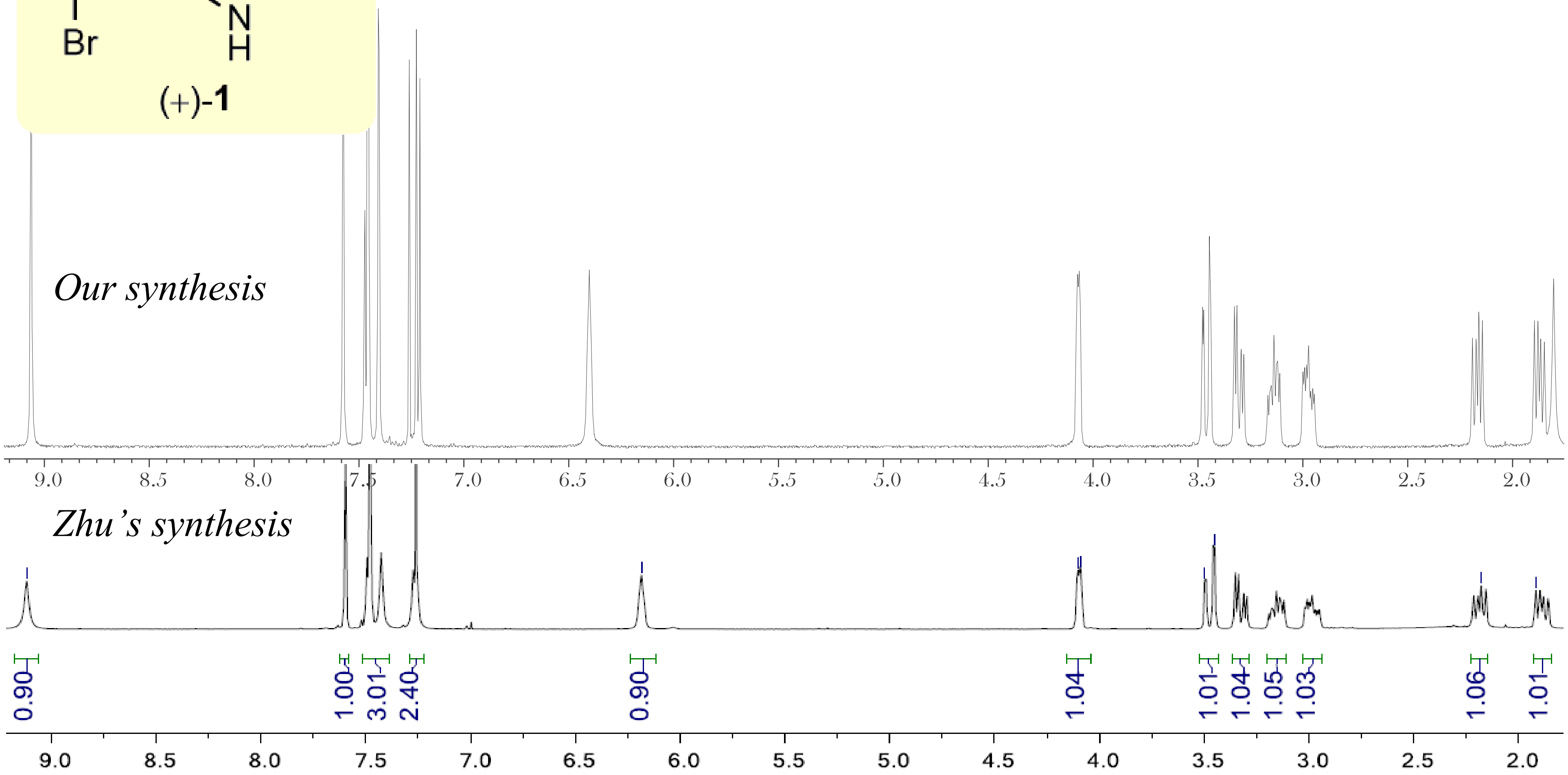

Stacked ^{1}H NMR spectra of (+)-hinckdentine A

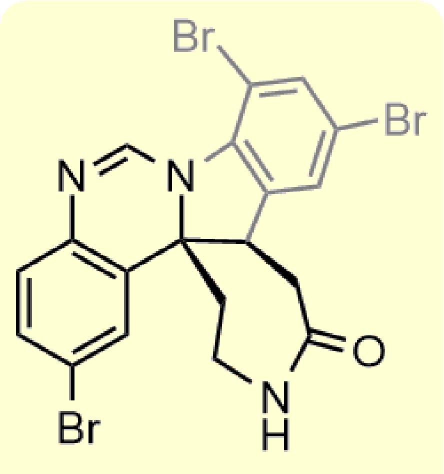

(+)-**1**

Our synthesis

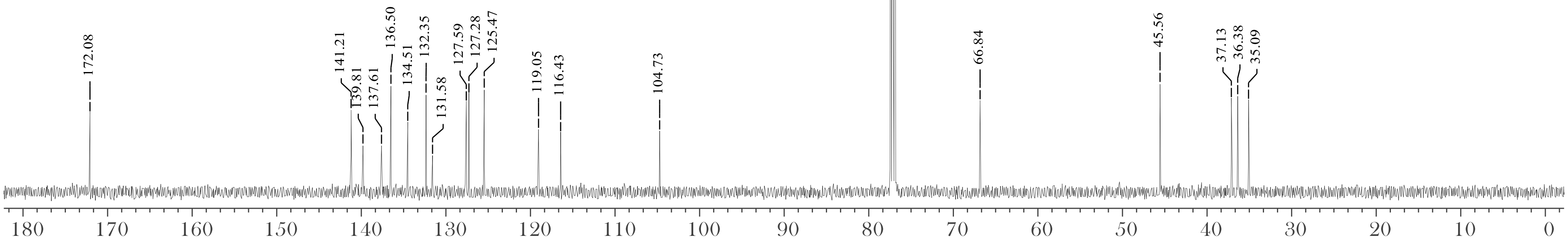

Zhu's synthesis

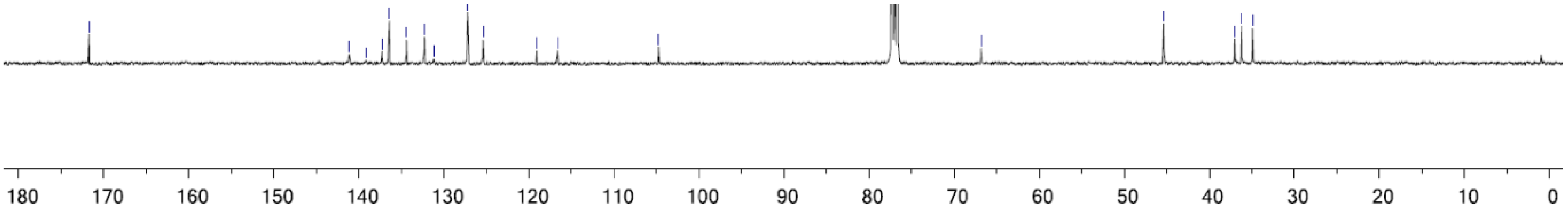

Stacked ^{13}C NMR spectra of (+)-hinckdentine A

Appendix

Kawasaki's synthesis of *rac*-hinckdentine A (2009):[10]

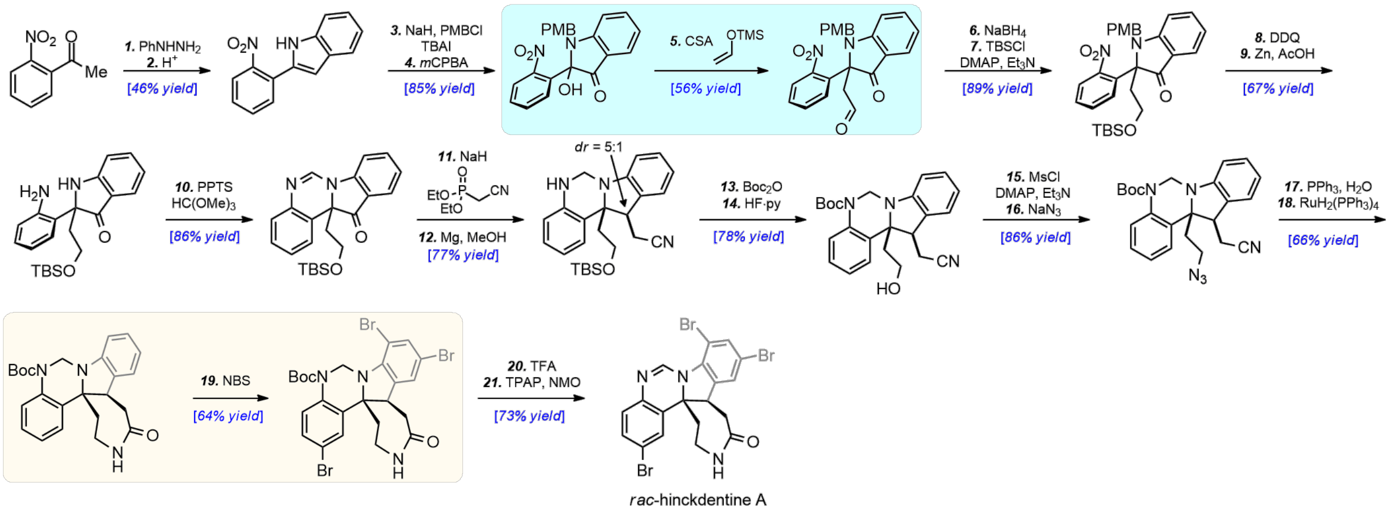

Fukuyama's synthesis of (+)-hinckdentine A (2016):[11]

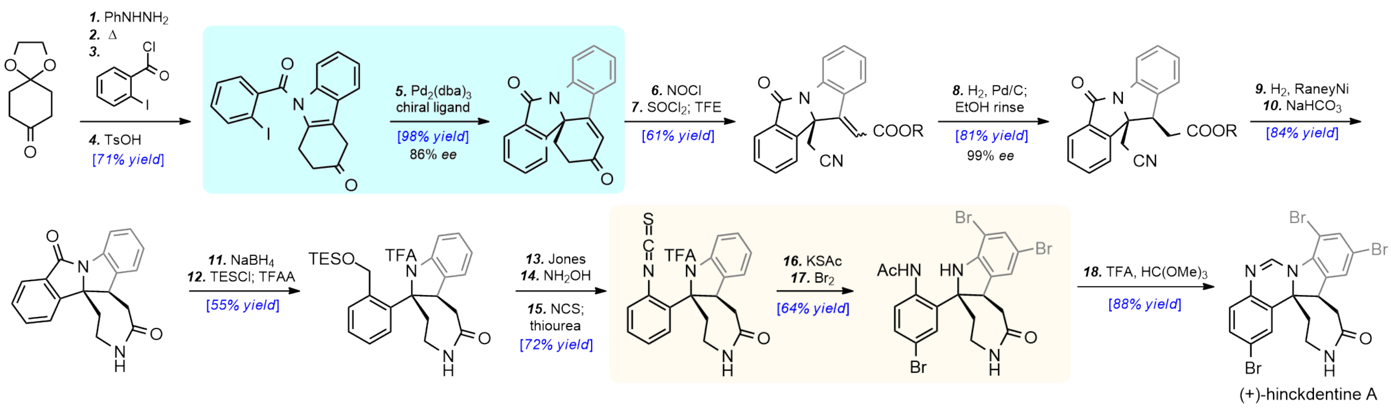

Zhu's synthesis of (+)-hinckdentine A (2018):[12]

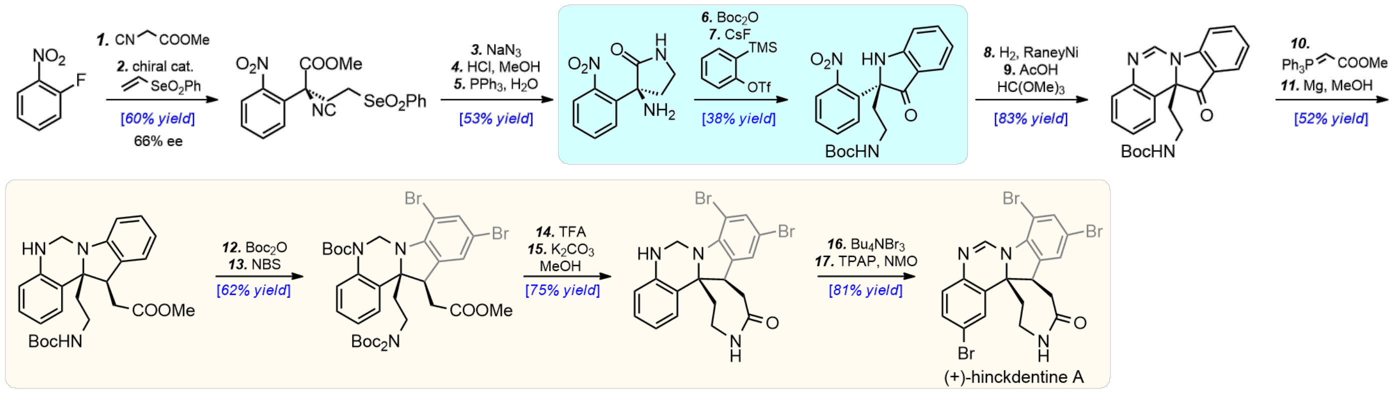

---

[10] Higuchi, K.; Sato, Y.; Tsuchimochi, M.; Sugiura, K.; Hatori, M.; Kawasaki, T. First total synthesis of hinckdentine A. *Org. Lett.* **2009**, *11*, 197−199

[11] Douki, K.; Ono, H.; Taniguchi, T.; Shimokawa, J.; Kitamura, M.; Fukuyama, T. Enantioselective total synthesis of (+)-hinckdentine A via a catalytic dearomatization approach. *J. Am. Chem. Soc.* **2016**, *138*, 14578−14581.

[12] Torres-Ochoa, R. O.; Buyck, T.; Wang, Q.; Zhu, J. Heteroannulation of arynes with α-amino imides: synthesis of 2,2-disubstituted indolin-3-ones and application to the enantioselective total synthesis of (+)-hinckdentine A. *Angew. Chem. Int. Ed.* **2018**, 57, 5679−5683.

Xu's synthesis of *rac*-hinckdentine A (2018):[13]

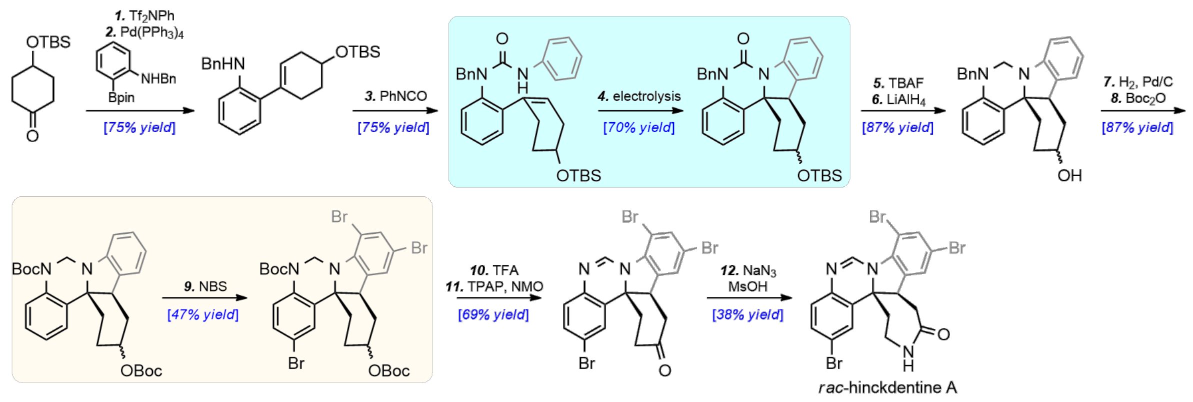

Cheon's synthesis of *rac*-hinckdentine A (2021):[14]

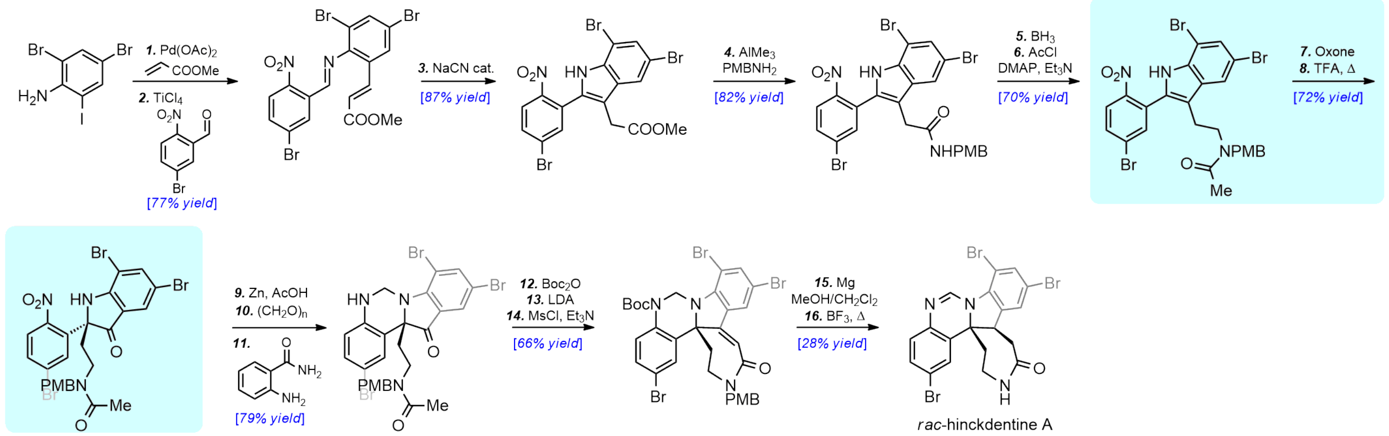

Hong's synthesis of (+)-hinckdentine A (2022):[15]

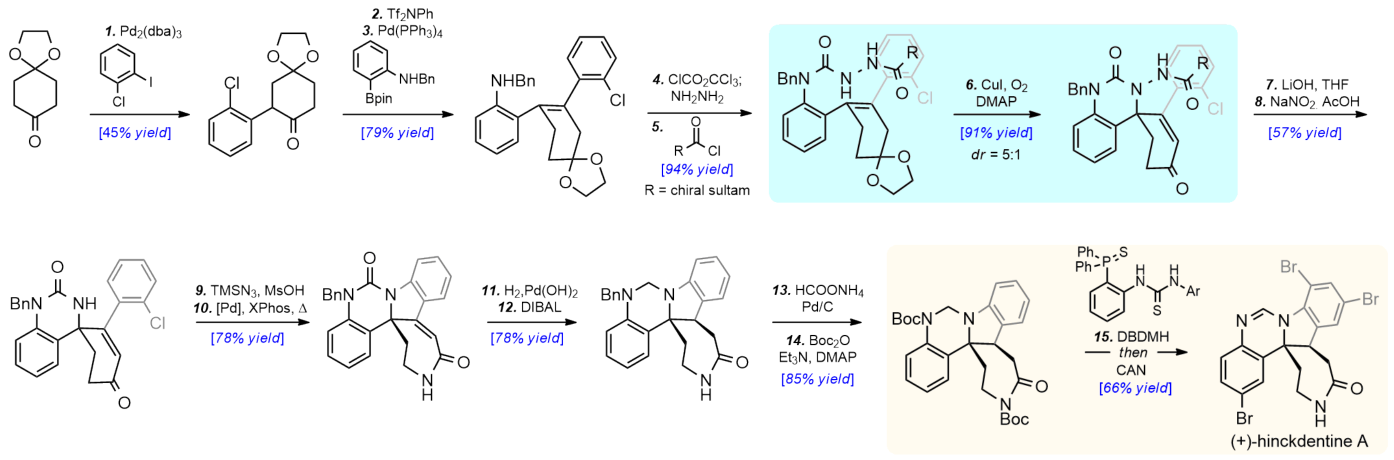

[13] Hou, Z.; Yan, H.; Song, J.; Xu, H. Electrochemical synthesis of (aza)indolines via dehydrogenative [3+2] annulation: application to total synthesis of (±)-hinckdentine A. *Chin. J. Chem.* **2018**, *36*, 909−915

[14] Jeon, J.; Lee, S. E.; Cheon, C.-H. Total synthesis of hinckdentine A. *Org. Lett.* **2021**, *23*, 2169−2173.

[15] Ruan, Z.; Wang, M.; Yang, C.; Zhu, L.; Su, Z.; Hong, R. Total synthesis of (+)-hinckdentine A: harnessing noncovalent interactions for organocatalytic bromination. *JACS Au* **2022**, *2*, 793-800.

Summary

chemist	series	*ee*	notes	# of steps[a]	overall yield, %
Kawasaki	racemic		difficult to render enantioselective	21	1.5
Fukuyama	+	>99%	the key step proceeded in 86% *ee*	18 (17)[b]	6.4
Zhu	+	66%	*ee* of the final product	17	3.2
Xu	racemic		difficult to render enantioselective	12	3
Cheon	racemic		difficult to render enantioselective	16	4
Hong	+	>99%	chiral auxiliary was used: *dr* = 5:1	15(12)[b]	5.6
this work	+	>99%	CBS reduction proceeded in 90% *ee*	9	32

[a] The steps for the above syntheses were counted by us in a manner consistent with generally accepted practices starting from commercially available materials. If procedures involved *sequential addition* of reagents to a reaction, then it was considered a single step. A work-up, solvent change or transfer of a reaction mixture to a different flask was considered the end of one step and the beginning of a new one.
[b] Reported step-count
